# Supplementary material for: Cholesterol-modified sphingomyelin chimeric lipid bilayer for improved therapeutic delivery
Source: Nat Commun. 2024 Mar 7;15:2073. doi: 10.1038/s41467-024-46331-7 (PMC10920917; doi:10.1038/s41467-024-46331-7)
Supplement: Supplementary file 1 — Supplementary Information [file 41467_2024_46331_MOESM1_ESM.pdf]

Supplementary Information

Cholesterol-Modified Sphingomyelin Chimeric Lipid Bilayer for Improved Therapeutic Delivery

Zhiren Wang[1#], Wenpan Li[1#], Yanhao Jiang[1], Jonghan Park[1], Karina Marie Gonzalez[1], Xiangmeng Wu[1], Qing-Yu Zhang[1,2], Jianqin Lu[1,2,3,4]*

[1]Skaggs Pharmaceutical Sciences Center, Department of Pharmacology & Toxicology, R. Ken Coit College of Pharmacy, The University of Arizona, Tucson, Arizona, 85721, United States

[2]Southwest Environmental Health Sciences Center, The University of Arizona, Tucson, 85721, United States

[3]Clinical and Translational Oncology Program (CTOP), The University of Arizona Cancer Center, Tucson, Arizona, 85721, United States

[4]BIO5 Institute, The University of Arizona, Tucson, Arizona, 85721, United States

[#]These authors contributed equally to this work
*Corresponding author: lu6@arizona.edu

Supplementary Methods

Materials

Doxorubicin hydrochloride (DOX, 98%), Di(1*H*-imidazol-1-yl)methanone (CDI, 98%), O-(7-Azabenzotriazol-1-yl)-N,N,N,N-tetramethyl uronium hexafluorophosphate (HATU, 98%), Irinotecan hydrochloride (IRI, 98%), Dexamethasone (DEX, 98%), Sodium 4-methyl-2-oxo-2*H*-chromen-7-yl phosphate (MU-P, 98%), 7-Hydroxy-4-methyl-2*H*-chromen-2-one (MU, 99%), 7-Hydroxy-2*H*-chromen-2-one (Internal standard, 98%), (2S,3S,4S,5R,6S)-3,4,5-Trihydroxy-6-((4-methyl-2-oxo-2H-chromen-7-yl)oxy)tetrahydro-2H-pyran-2-carboxylic acid (MU-G, 98%) and (tert-butoxycarbonyl)glycine (98%) were purchased from BLDpharm (Shanghai, China). Vincristine sulfate (VCR, 98%), (6Z,9Z,28Z,31Z)-Heptatriaconta-6,9,28,31-tetraen-19-yl 4-(dimethylamino)butanoate (DLin-MC3-DMA, 98%) and 2-(((3*S*,8*S*,9*S*,10*R*,13*R*,14*S*,17*R*)-10,13-dimethyl-17-((*R*)-6-methylheptan-2-yl)-2,3,4,7,8,9,10,11,12,13,14,15,16,17-tetradecahydro-1*H*-cyclopenta[a]phenanthren-3-yl)disulfaneyl)acetic acid (Chol-SS-COOH) were purchased from WuXi App Tec (Shanghai, China). Sphingomyelin (SM, egg, 99%), 1,2-distearoyl-sn-glycero-3-phosphoethanolamine-N-[methoxy(polyethylene glycol)-2000] ammonium salt ($DSPE\text{-}PEG_{2K}$, 99%), L-α-phosphatidylcholine, hydrogenated (Soy) (HSPC, 99%), L-α-phosphatidylcholine (Soy) (SPC, 99%), 1,2-distearoyl-sn-glycero-3-phosphocholine (DSPC, 99%), 1,2-dipalmitoyl-sn-glycero-3-phosphocholine (DPPC, 99%), 1,2-dioleoyl-sn-glycero-3-phosphocholine (DOPC, 99%), (*R*)-2-hydroxy-3-(palmitoyloxy)propyl (2-(trimethylammonio)ethyl) phosphate, 1-palmitoyl-2-cholesterylcarbonoyl-sn-glycero-3-phosphocholine (PChcPC, 99%), 1-palmitoyl-2-cholesterylhemisuccinoyl-sn-glycero-3-phosphocholine (PChemsPC, 99%), 1-oleoyl-2-cholesterylhemisuccinoyl-sn-glycero-3-phosphocholine (OChemsPC, 99%), 1,2-dicholesterylhemisuccinoyl-sn-glycero-3-phosphocholine (DChemsPC, 99%), 1,2-dioleoyl-sn-glycero-3-phospho-(1'-rac-glycerol) sodium salt (PG, 99%), 1-palmitoyl-2-oleoyl-glycero-

3-phosphocholine (POPC, 99%) and cholesterol (Chol, 99%) were purchased from Avanti (Alabama, USA). 1,2-dimyristoyl-rac-glycero-3-carbonylaminoethyl-ω-methoxypolyethylene glycol-2000 (PEG_{2K}-C-DMG, 97%) was purchased from BOC Sciences (NY, USA). Succinic anhydride (98%), N, N-diisopropylethylamine (98%), 4-Dimethylaminopyridine (DMAP, 98%), triphosgene (98%), 4-pyrrolidinopyridine (4-PPY, 98%), 1-ethyl-3-(3-dimethylaminopropyl)carbodiimide hydrochloride (EDCI, 98%), citric acid (99%), Dowex 50Wx8-200, triethylamine (TEA, 99%), 2-mercaptoethan-1-ol (98%), Decaethylene glycol mono-dodecyl ether ($C_{12}E_{10}$, 98%) and 2, 2'-dithiodiethanol (98%) were purchased from Sigma-Aldrich (MO, USA). 1,1'-Dioctadecyl-3,3,3',3'-Tetramethylindodicarbocyanine, 4-Chlorobenzenesulfonate Salt (DiD, 99%) was purchased from Invitrogen. Sucrose octasulfate (SOS) sodium salt was purchased from Toronto Research Chemicals, Inc., Canada. Lipopolysaccharide (LPS) was purchased from Santa Cruz Biotechnology (USA). Matrigel was purchased from Corning, Discovery labware Inc. (USA). Doxil and Onivyde were acquired from Pharmacy Department, Banner-University Medical Center Tucson. Trypsin-EDTA solution, Triton X-100, and Dulbecco's Modified Eagle's Medium (DMEM), RPMI-1640 medium, fetal bovine serum (FBS), and penicillin-streptomycin solution were all purchased from Gibco (MD, USA). All solvents used for chemical reactions were anhydrous, and the eluting solvents for compound purification were HPLC grade.

Chemical synthesis

The NMR spectra were acquired by Bruker topspin software (v. 2.1) using TMS (0 ppm) as the internal standard on a AVIII 400 MHz spectrometer and analysed by MestReNova (v. 6.0.2). ^{1}H NMR data were reported as follows: chemical shift, multiplicity (s = singlet, d = doublet, m = multiplet), coupling constant in Hertz (Hz) and hydrogen numbers based on integration intensities. ^{13}C NMR chemical shifts are reported in ppm relative to the central

peak of TMS (0 ppm) as internal standards. The high-resolution mass spectra (HRMS) were generated via a LTQ Orbitrap Velos mass spectrometer with an ESI source (Thermo Scientific). The low-resolution mass spectra were generated using a LCMS-2020 + DUIS-2020 (Shimadzu). The reactions were followed by thin-layer chromatography (TLC, Silica gel 60 F_{254}, Merck KGaA) on glass-packed precoated silica gel plates and visualized in an iodine chamber or with a UV lamp. Flash column chromatography was performed using silica gel (SiliaFlash® P60, 230–400 mesh) purchased from Silicycle Inc.

The synthesis of SM-C-Ester-Chol

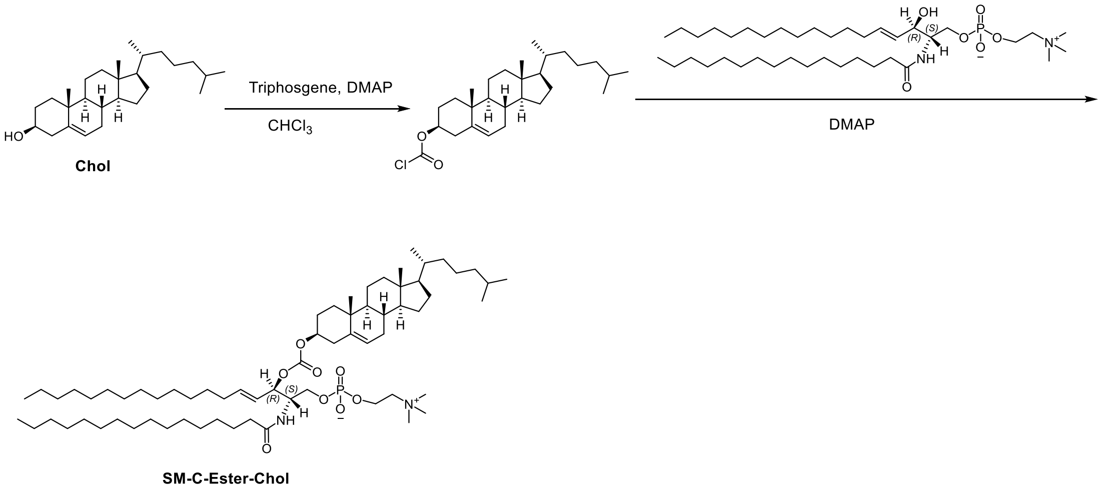

Supplementary Figure 1, Synthetic route for SM-derived Chol with carbonate ester bond (SM-C-Ester-Chol).

(2*S*,3*R*,*E*)-3-(((((3*S*,8*S*,9*S*,10*R*,13*R*,14*S*,17*R*)-10,13-dimethyl-17-((*R*)-6-methylheptan-2-yl)-2,3,4,7,8,9,10,11,12,13,14,15,16,17-tetradecahydro-1*H*-cyclopenta[a]phenanthren-3-yl)oxy)carbonyl)oxy)-2-palmitamidooctadec-4-en-1-yl (2-(trimethylammonio)ethyl) phosphate (SM-C-Ester-Chol)

Triphosgene (207.7 mg, 0.7 mmol) and DMAP (488.6 mg, 4 mmol) was added to a solution of Chol (773.0 mg, 2.0 mmol) in anhydrous $CHCl_3$, the solution was stirred at room temperature for 30 min. Sphingomyelin (703.0 mg, 1.0 mmol) was added into the mixture

solution, the reaction was stirred at room temperature for 48 h and monitored by TLC. The solvent was then removed under reduced pressure, the product was extracted by DCM (50 mL × 5). The organic phase was washed with saturated brine, dried with anhydrous Na_2SO_4, and the solvent was evaporated using rotary evaporator (RV 10 digital, IKA®) under vacuum followed by purification by silica gel flash chromatography with $CHCl_3$/EtOH/H_2O (v/v/v, 300/200/36) as the eluting solvent. White solid with 35% yield was obtained. R_f = 0.28 ($CHCl_3$/EtOH/H_2O = 300/200/36). ^{1}H NMR (400 MHz, $CDCl_3$) δ 7.47 (d, J = 7.8 Hz, 1H), 5.82 – 5.71 (m, 1H), 5.46 (dd, J = 15.3, 7.9 Hz, 1H), 5.39 – 5.35 (m, 1H), 5.15 (t, J = 7.6 Hz, 1H), 4.34 (dt, J = 8.1, 7.7 Hz, 4H), 4.04 – 3.93 (m, 2H), 3.85 (s, 2H), 3.37 (s, 9H), 2.37 (d, J = 6.7 Hz, 2H), 2.16 (t, J = 7.3 Hz, 2H), 2.09 – 1.95 (m, 5H), 1.87 (dd, J = 17.8, 7.7 Hz, 3H), 1.70 – 1.43 (m, 11H), 1.39 – 1.21 (m, 60H), 1.19 – 1.05 (m, 8H), 1.03 (d, J = 7.3 Hz, 5H), 0.96 – 0.85 (m, 18H), 0.69 (s, 3H). ^{13}C NMR (101 MHz, $CDCl_3$) δ 173.69, 153.60, 139.33, 137.89, 124.30, 123.02, 77.97, 66.42, 66.36, 63.82, 63.78, 59.36, 59.31, 56.71, 56.22, 54.47, 51.59, 51.54, 50.00, 42.33, 39.74, 39.53, 38.09, 36.86, 36.73, 36.56, 36.22, 35.83, 32.40, 31.98, 31.93, 31.86, 29.91, 29.87, 29.84, 29.82, 29.76, 29.66, 29.44, 29.36, 28.99, 28.25, 28.03, 27.77, 26.01, 24.31, 23.91, 22.82, 22.73, 22.57, 21.07, 19.28, 18.71, 14.14, 11.87. HRMS (ESI) m/z $[M + H]^+$ for $C_{67}H_{123}N_2O_8P$ calculated 1115.9053, found 1115.9089.

The synthesis of SM-Ester-Chol

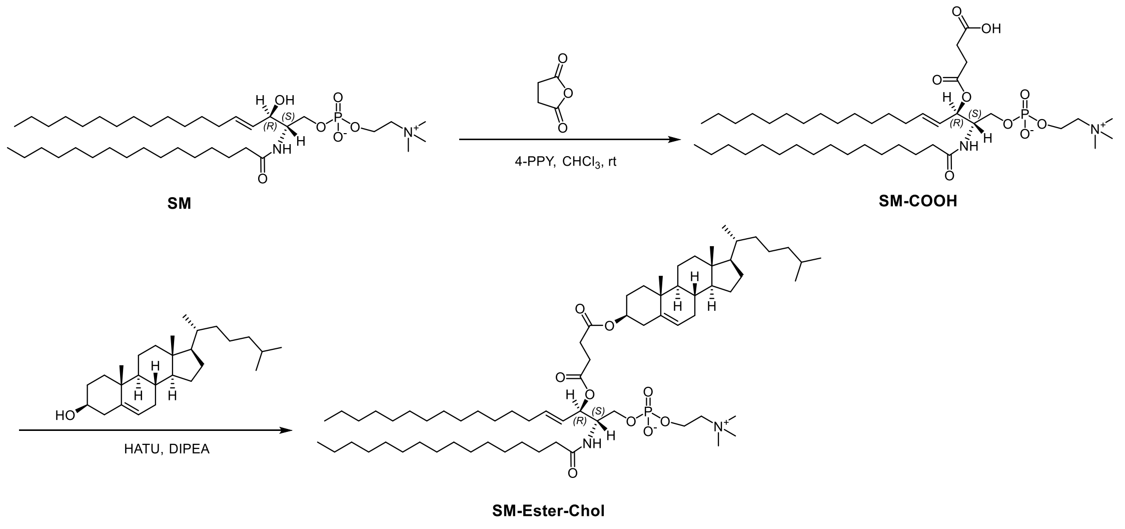

Supplementary Figure 2, Synthetic route for SM-derived Chol with ester bond (SM-Ester-Chol).

(2*S*,3*R*,*E*)-3-((3-carboxypropanoyl)oxy)-2-palmitamidooctadec-4-en-1-yl (2-(trimethylammonio)ethyl) phosphate (SM-COOH)

The synthesis of SM-COOH was according to our previous publication.[1] 4-pyrrolidinopyridine (4-PPY, 44.4 mg, 0.3 mmol) was added to a solution of sphingomyelin (2.1 g, 3.0 mmol) and succinic anhydride (3 g, 30 mmol) in anhydrous $CHCl_3$ (100 mL). The solution was stirred at room temperature for 12 h and monitored by TLC. After completion of the reaction, CH_3OH (30 mL) was added into the mixture solution, the reaction was further stirred at room temperature for 12 h. The solvent was evaporated using rotary evaporator under vacuum, and the residue was purified by silica gel flash chromatography with $CHCl_3$/EtOH/H_2O (v/v/v, 300/200/36) as the elution solvent. White solid with 93% yield was garnered. R_f = 0.23 ($CHCl_3$/EtOH/H_2O = 300/200/36). ^{1}H NMR (400 MHz, $CDCl_3$) δ 6.98 (d, *J* = 6.7 Hz, 1H), 5.75 – 5.68 (m, 1H), 5.39 (dd, *J* = 15.0, 8.3 Hz, 1H), 5.28 (t, *J* = 8.8 Hz, 1H), 4.29 (d, *J* = 4.4 Hz, 3H), 3.94 (s, 2H), 3.77 (s, 2H), 3.28 (s, 9H), 2.64 (dd, *J* = 13.1, 6.3 Hz, 2H), 2.39 (dd, *J* = 14.3, 6.6 Hz, 2H), 2.12 (q, *J* = 13.9 Hz, 2H), 1.97 (d, *J* = 6.6 Hz, 2H), 1.55 (s, 2H), 1.28 (d, *J* = 19.0 Hz, 46H), 0.88 (t, *J* = 6.7 Hz, 6H). ^{13}C NMR (101 MHz, $CDCl_3$) δ 174.74, 173.10, 172.06, 137.75, 125.20, 73.28, 65.76, 65.70, 64.26, 64.23, 59.20, 59.15, 54.32, 50.66, 50.61, 36.66, 32.25, 31.88, 29.74, 29.71, 29.70, 29.69, 29.63, 29.60, 29.56, 29.50, 29.46, 29.33, 28.92, 25.79, 22.64, 14.06. HRMS (ESI) m/z $[M + H]^+$ for $C_{43}H_{84}N_2O_9P$ calculated 803.5909, found 803.5928.

(2*S*,3*R*,*E*)-3-((4-(((3*S*,8*S*,9*S*,10*R*,13*R*,14*S*,17*R*)-10,13-dimethyl-17-((*R*)-6-methylheptan-2-yl)-2,3,4,7,8,9,10,11,12,13,14,15,16,17-tetradecahydro-1*H*-cyclopenta[*a*]phenanthren-3-yl)oxy)-4-oxobutanoyl)oxy)-2-palmitamidooctadec-4-en-1-yl (2-(trimethylammonio)ethyl) phosphate (SM-Ester-Chol)

DIPEA (2 mL) was added to a solution of SM-COOH (803.0 mg, 1.0 mmol) and HATU (456 mg, 1.5 mmol) in anhydrous DCM (50 mL). The reaction mixture was stirred at room temperature for 30 min. A solution of Chol (386 mg, 1.0 mmol) in 10 mL anhydrous DCM was added into the reaction and further stirred for 12 h. The reaction was monitored by TLC, after completion of the reaction, the reaction mixture was washed with 50 mM HCl aqueous solution, and then with saturated brine. The organic layer was dried with anhydrous Na_2SO_4, the solvent was removed using rotary evaporator under vacuum, and the residue was purified by silica gel flash chromatography with $CHCl_3$/EtOH/H_2O (v/v/v, 300/200/36) as the eluting solvent. White solid with 54% yield was achieved. R_f = 0.29 ($CHCl_3$/EtOH/H_2O = 300/200/36). ^{1}H NMR (400 MHz, $CDCl_3$) δ 7.36 (d, *J* = 8.9 Hz, 1H), 5.82 – 5.68 (m, 1H), 5.53 – 5.26 (m, 3H), 4.66 – 4.47 (m, 1H), 4.39 – 4.26 (m, 3H), 4.01 – 3.89 (m, 2H), 3.82 (s, 2H), 3.36 (s, 9H), 2.59 (dt, *J* = 14.1, 7.4 Hz, 4H), 2.33 (t, *J* = 11.7 Hz, 2H), 2.18 (dd, *J* = 14.8, 8.2 Hz, 2H), 2.07 – 1.95 (m, 4H), 1.92 – 1.75 (m, 3H), 1.70 – 1.43 (m, 9H), 1.39 – 1.21 (m, 57H), 1.20 – 0.98 (m, 11H), 0.94 (dd, *J* = 13.5, 7.0 Hz, 6H), 0.90 – 0.87 (m, 9H), 0.70 (s, 3H).^{13}C NMR (101 MHz, $CDCl_3$) δ 173.42, 171.71, 171.42, 139.49, 137.27, 124.53, 122.79, 77.23, 74.42, 74.10, 66.45, 63.95, 59.24, 56.68, 56.17, 54.56, 51.32, 50.02, 42.31, 39.71, 39.50, 38.08, 36.97, 36.80, 36.58, 36.18, 35.80, 32.40, 31.94, 31.90, 31.84, 29.79, 29.78, 29.71, 29.61, 29.45, 29.39, 29.04, 28.22, 28.00, 27.76, 25.93, 24.28, 23.87, 22.80, 22.70, 22.55, 21.03, 19.29, 18.70, 14.12, 11.84. HRMS (ESI) m/z $[M + H]^+$ for $C_{70}H_{127}N_2O_9P$ calculated 1171.9343, found 1171.9352.

The synthesis of SM-Glycine-Chol

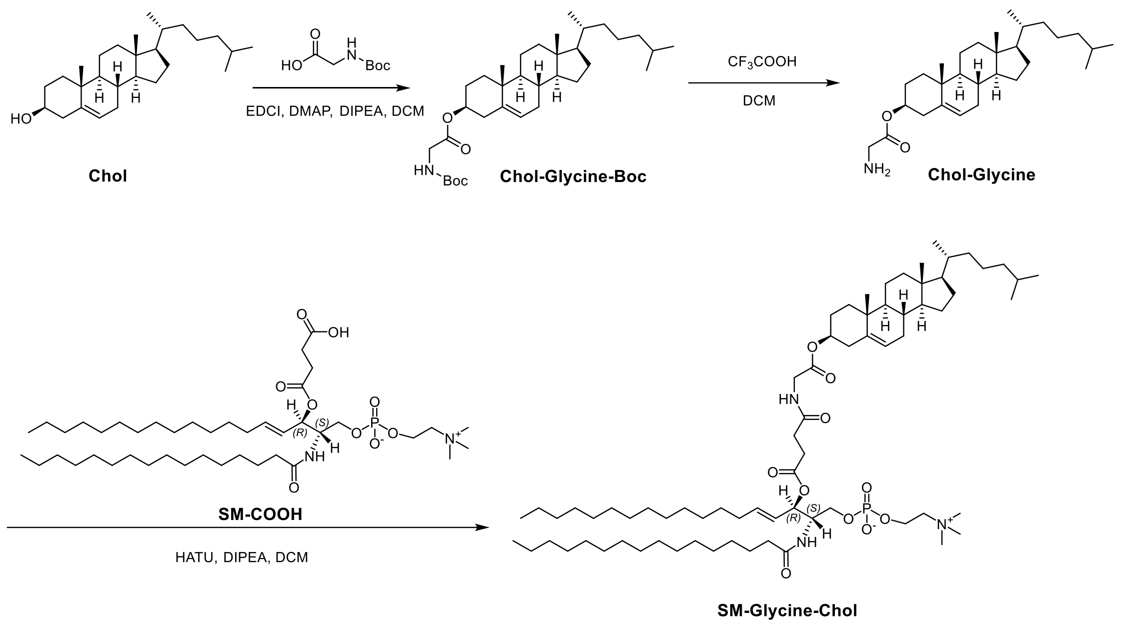

Supplementary Figure 3, Synthetic route for SM-derived Chol with glycine bond (SM-Glycine-Chol).

(3*S*,8*S*,9*S*,10*R*,13*R*,14*S*,17*R*)-10,13-dimethyl-17-((*R*)-6-methylheptan-2-yl)-2,3,4,7,8,9,10,11,12,13,14,15,16,17-tetradecahydro-1*H*-cyclopenta[*a*]phenanthren-3-yl (tert-butoxycarbonyl)glycinate (Chol-Glycine-Boc)

EDCI (2.10g, 11.0 mmol) and 2 mL DIPEA was added to a solution of (*tert*-butoxycarbonyl)glycine (1.75g, 10.0 mmol) in 100 mL anhydrous DCM followed by stirring at room temperature for 30 min. Chol (3.86 g, 10 mmol) and DMAP (122 mg, 1.0 mmol) was then added into the mixture solution. The reaction was further stirred at room temperature for 12 h and monitored by TLC. After completion of the reaction, the mixture solution was washed with 50 mM HCl aqueous solution to remove the DMAP, and then with saturated brine. The organic layer was dried with anhydrous Na_2SO_4, the solvent was evaporated using rotary evaporator under vacuum, and the residue was purified by silica gel flash chromatography. White solid with 82% yield was gained. R_f = 0.51 (petroleum/EtOAc = 2/1). ^{1}H NMR (400 MHz, $CDCl_3$) δ 5.40 (d, *J* = 3.9 Hz, 1H), 5.02 (s, 1H), 4.78 – 4.63 (m, 1H), 3.90 (d, *J* = 5.3 Hz, 2H), 2.36 (d, *J* = 7.6 Hz, 2H), 2.08 – 1.95 (m, 2H), 1.85 (ddd, *J* = 14.6,

13.3, 8.2 Hz, 3H), 1.67 – 1.49 (m, 6H), 1.48 (s, 9H), 1.47 – 1.24 (m, 5H), 1.23 – 1.07 (m, 7H), 1.04 (d, *J* = 5.6 Hz, 4H), 0.94 (d, *J* = 6.5 Hz, 3H), 0.88 (ddd, *J* = 14.4, 6.6, 3.3 Hz, 6H), 0.71 (s, 3H). ^{13}C NMR (101 MHz, $CDCl_3$) δ 169.74, 155.68, 139.37, 122.93, 79.89, 77.22, 75.17, 56.69, 56.14, 50.01, 42.68, 42.31, 39.72, 39.52, 38.02, 36.91, 36.56, 36.19, 35.79, 31.90, 31.85, 28.33, 28.22, 28.01, 27.71, 24.28, 23.83, 22.82, 22.56, 21.03, 19.29, 18.72, 11.86. LC/MS (ESI): 544.4 $[M + H]^+$.

(3*S*,8*S*,9*S*,10*R*,13*R*,14*S*,17*R*)-10,13-dimethyl-17-((*R*)-6-methylheptan-2-yl)-2,3,4,7,8,9,10,11,12,13,14,15,16,17-tetradecahydro-1*H*-cyclopenta[*a*]phenanthren-3-yl glycinate (Chol-Glycine)

CF_3COOH (1 mL) was added to a solution of Chol-Glycine-Boc (543 mg, 1.0 mmol) in anhydrous DCM (50 mL) in an ice bath. The reaction was stirred at room temperature for 0.5 h and monitored by TLC. After completion of the reaction, the solvent was evaporated using rotary evaporator under vacuum. This intermediate was used for the next step immediately without further purification.

(2*S*,3*R*,*E*)-3-((4-((2-(((3*S*,8*S*,9*S*,10*R*,13*R*,14*S*,17*R*)-10,13-dimethyl-17-((*R*)-6-methylheptan-2-yl)-2,3,4,7,8,9,10,11,12,13,14,15,16,17-tetradecahydro-1*H*-cyclopenta[*a*]phenanthren-3-yl)oxy)-2-oxoethyl)amino)-4-oxobutanoyl)oxy)-2-palmitamidooctadec-4-en-1-yl (2-(trimethylammonio)ethyl) phosphate (SM-Glycine-Chol)

DIPEA (2 mL) was added to a solution of SM-COOH (803.0 mg, 1.0 mmol) and HATU (380 mg, 1.0 mmol) in anhydrous DCM (50 mL). The reaction mixture was stirred at room temperature for 30 min. A solution of Chol-Glycine (1.0 mmol) in 10 mL anhydrous DCM was added into the reaction and further stirred for 12 h. After completion of the reaction, the reaction mixture was washed with 50 mM HCl aqueous solution, and then with saturated brine. The organic layer was dried with anhydrous Na_2SO_4, the solvent was

removed using rotary evaporator under vacuum, and the residue was purified by silica gel flash chromatography with $CHCl_3$/EtOH/H_2O (v/v/v, 300/200/36) as the eluting solvent. White solid with 83% yield was achieved. R_f = 0.35 ($CHCl_3$/EtOH/H_2O = 300/200/36). ^{1}H NMR (400 MHz, $CDCl_3$) δ 8.13 (s, 1H), 7.22 (s, 1H), 5.76 – 5.67 (m, 1H), 5.43 – 5.28 (m, 3H), 4.64 – 4.55 (m, 1H), 4.37 – 4.25 (m, 3H), 3.99 – 3.86 (m, 4H), 3.82 (s, 2H), 3.35 (s, 9H), 2.62 (dt, *J* = 18.8, 11.5 Hz, 4H), 2.32 (d, *J* = 6.1 Hz, 2H), 2.14 (s, 2H), 2.00 (dd, *J* = 18.2, 11.6 Hz, 4H), 1.90 – 1.80 (m, 3H), 1.66 – 1.43 (m, 10H), 1.42 – 1.19 (m, 60H), 1.12 (dd, *J* = 20.4, 13.5 Hz, 8H), 1.02 (s, 5H), 0.96 – 0.85 (m, 18H), 0.69 (s, 3H). ^{13}C NMR (101 MHz, $CDCl_3$) δ 173.52 (s), 172.17 (d, *J* = 3.7 Hz), 169.89 (s), 139.44 (s), 137.18 (s), 124.49 (s), 122.84 (s), 77.27 (s), 75.02 (s), 73.71 (s), 66.37 (d, *J* = 6.3 Hz), 64.33 (s), 59.31 (d, *J* = 1.6 Hz), 56.72 (s), 56.21 (s), 54.41 (s), 51.39 (d, *J* = 6.2 Hz), 50.02 (s), 42.33 (s), 41.57 (s), 39.63 (d, *J* = 21.9 Hz), 39.51 – 39.42 (m), 38.06 (s), 36.82 (d, *J* = 25.8 Hz), 36.58 (s), 36.21 (s), 35.82 (s), 32.45 (s), 32.06 – 31.61 (m), 30.38 (s), 29.99 – 29.13 (m), 29.07 (s), 28.13 (d, *J* = 21.9 Hz), 27.73 (s), 26.00 (s), 24.29 (s), 23.90 (s), 22.70 (t, *J* = 12.8 Hz), 21.04 (s), 19.29 (s), 18.72 (s), 14.14 (s), 11.85 (s). HRMS (ESI) m/z $[M + H]^+$ for $C_{72}H_{130}N_3O_{10}P$ calculated 1228.9519, found 1228.9566.

The synthesis of SM-CSS-Chol

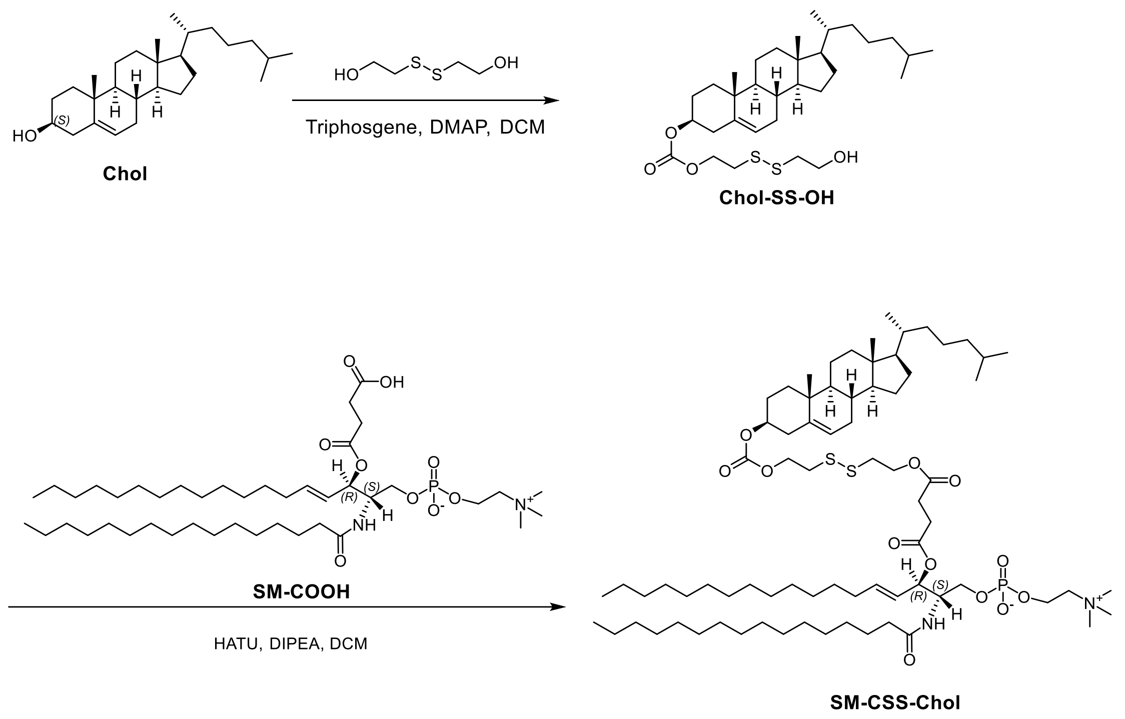

Supplementary Figure 4, Synthetic route for SM-derived Chol with disulfide bond and longer linker (SM-CSS-Chol).

(3*S*,8*S*,9*S*,10*R*,13*R*,14*S*,17*R*)-10,13-dimethyl-17-((*R*)-6-methylheptan-2-yl)-2,3,4,7,8,9,10,11,12,13,14,15,16,17-tetradecahydro-1*H*-cyclopenta[*a*]phenanthren-3-yl (2-((2-hydroxyethyl)disulfanyl)ethyl) carbonate (Chol-SS-OH)

DMAP (3.67 g, 30 mmol, in 15 mL anhydrous DCM) was added dropwise to a solution of Chol (3.86 g, 10 mmol) and triphosgene (1.03 g, 3.4 mmol) in anhydrous DCM (150 mL). The reaction was stirred at room temperature for 30 min, then a solution of 2, 2'-dithiodiethanol (9.25 g, 60 mmol) in anhydrous THF (25 mL) was added into the mixture solution. The reaction was further stirred at room temperature for 12 h and monitored by TLC. After completion of the reaction, the mixture solution was washed with 50 mM HCl aqueous solution to remove the DMAP, and then with saturated brine. The organic layer was dried with anhydrous Na_2SO_4, the solvent was evaporated using rotary evaporator under vacuum, and the residue was purified by silica gel flash chromatography. White solid with 92% yield was acquired. R_f = 0.42 (petroleum/EtOAc = 4/1). ^{1}H NMR (400 MHz, $CDCl_3$) δ 5.42 (d, *J* =

5.1 Hz, 1H), 4.57 – 4.45 (m, 1H), 4.41 (t, *J* = 6.7 Hz, 2H), 3.92 (q, *J* = 5.8 Hz, 2H), 2.99 (t, *J* = 6.7 Hz, 2H), 2.92 (t, *J* = 5.8 Hz, 2H), 2.50 – 2.33 (m, 2H), 2.10 (t, *J* = 6.1 Hz, 1H), 2.07 – 1.78 (m, 5H), 1.74 – 1.65 (m, 1H), 1.65 – 1.45 (m, 7H), 1.43 – 1.26 (m, 4H), 1.26 – 1.07 (m, 7H), 1.07 – 1.00 (m, 5H), 0.94 (d, *J* = 6.5 Hz, 4H), 0.89 (dd, *J* = 6.6, 1.7 Hz, 6H), 0.71 (s, 3H). ^{13}C NMR (101 MHz, $CDCl_3$) δ 154.35, 139.24, 123.08, 78.25, 77.22, 65.39, 60.20, 56.69, 56.14, 50.00, 42.32, 41.68, 39.72, 39.52, 38.00, 36.88, 36.84, 36.54, 36.19, 35.79, 31.90, 31.84, 28.22, 28.02, 27.68, 24.28, 23.83, 22.82, 22.57, 21.05, 19.27, 18.72, 11.86. LC/MS (ESI): 567.3 $[M + H]^+$.

(15*R*,16*S*)-1-(((3*S*,8*S*,9*S*,10*R*,13*R*,14*S*,17*R*)-10,13-dimethyl-17-((*R*)-6-methylheptan-2-yl)-2,3,4,7,8,9,10,11,12,13,14,15,16,17-tetradecahydro-1*H*-cyclopenta[*a*]phenanthren-3-yl)oxy)-1,10,13-trioxo-16-palmitamido-15-((*E*)-pentadec-1-en-1-yl)-2,9,14-trioxa-5,6-dithiaheptadecan-17-yl (2-(trimethylammonio)ethyl) phosphate (SM-CSS-Chol)

DIPEA (2 mL) was added to a solution of SM-COOH (803.0 mg, 1.0 mmol) and HATU (380 mg, 1.0 mmol) in anhydrous DCM (50 mL). The solution mixture was stirred at room temperature for 30 min. A solution of Chol-SS-OH (567 mg, 1.0 mmol) in 10 mL anhydrous DCM was then added into the reaction mixture and further stirred for 12 h. After completion of the reaction, the reaction mixture was washed with 50 mM HCl aqueous solution and then with saturated brine. The organic layer was dried with anhydrous Na_2SO_4, the solvent was evaporated using rotary evaporator under vacuum, and the residue was purified by silica gel flash chromatography with $CHCl_3$/EtOH/H_2O (v/v/v, 300/200/36) as the eluting solvent. White solid with 86% yield was attained. R_f = 0.35 ($CHCl_3$/EtOH/H_2O = 300/200/36). ^{1}H NMR (400 MHz, $CDCl_3$) δ 7.26 (d, *J* = 8.8 Hz, 1H), 5.77 – 5.68 (m, 1H), 5.41 (dd, *J* = 12.6, 6.3 Hz, 2H), 5.31 (t, *J* = 7.6 Hz, 1H), 4.48 (dt, *J* = 10.2, 4.9 Hz, 1H), 4.39 – 4.28 (m, 7H), 3.94 – 3.88 (m, 2H), 3.80 (s, 2H), 3.34 (s, 9H), 2.97 – 2.91 (m, 4H), 2.66 – 2.57 (m, 4H), 2.40 (q, *J* = 8.0 Hz, 2H), 2.15 (t, *J* = 7.3 Hz, 2H), 2.07 – 1.88 (m, 6H), 1.72 –

1.39 (m, 10H), 1.37 – 1.19 (m, 55H), 1.12 (ddd, J = 22.6, 14.3, 5.2 Hz, 7H), 1.05 – 0.95 (m, 6H), 0.89 (ddd, J = 8.8, 8.4, 4.2 Hz, 16H), 0.69 (s, 3H). ^{13}C NMR (101 MHz, $CDCl_3$) δ 173.45 (s), 172.18 (s), 171.24 (s), 154.24 (s), 139.22 (s), 137.39 (s), 124.49 (s), 123.06 (s), 78.18 (s), 77.34 (s), 74.15 (s), 66.37 (d, J = 5.8 Hz), 65.27 (s), 63.95 (d, J = 2.3 Hz), 62.55 (s), 59.30 (d, J = 4.5 Hz), 56.69 (s), 56.15 (s), 54.49 (s), 51.30 (d, J = 5.3 Hz), 50.00 (s), 42.31 (s), 39.71 (s), 39.51 (s), 38.01 (s), 36.91 (dd, J = 18.3, 5.5 Hz), 36.53 (s), 36.19 (s), 35.79 (s), 32.40 (s), 32.04 – 31.70 (m), 30.01 – 29.20 (m), 29.03 (s), 28.22 (s), 28.00 (s), 27.69 (s), 25.95 (s), 24.28 (s), 23.84 (s), 22.69 (t, J = 12.8 Hz), 21.05 (s), 19.26 (s), 18.71 (s), 14.13 (s), 11.85 (s). HRMS (ESI) m/z $[M + H]^+$ for $C_{75}H_{135}N_2O_{12}PS_2$ calculated 1351.9216, found 1351.9266.

The synthesis of SM-SCS-Chol

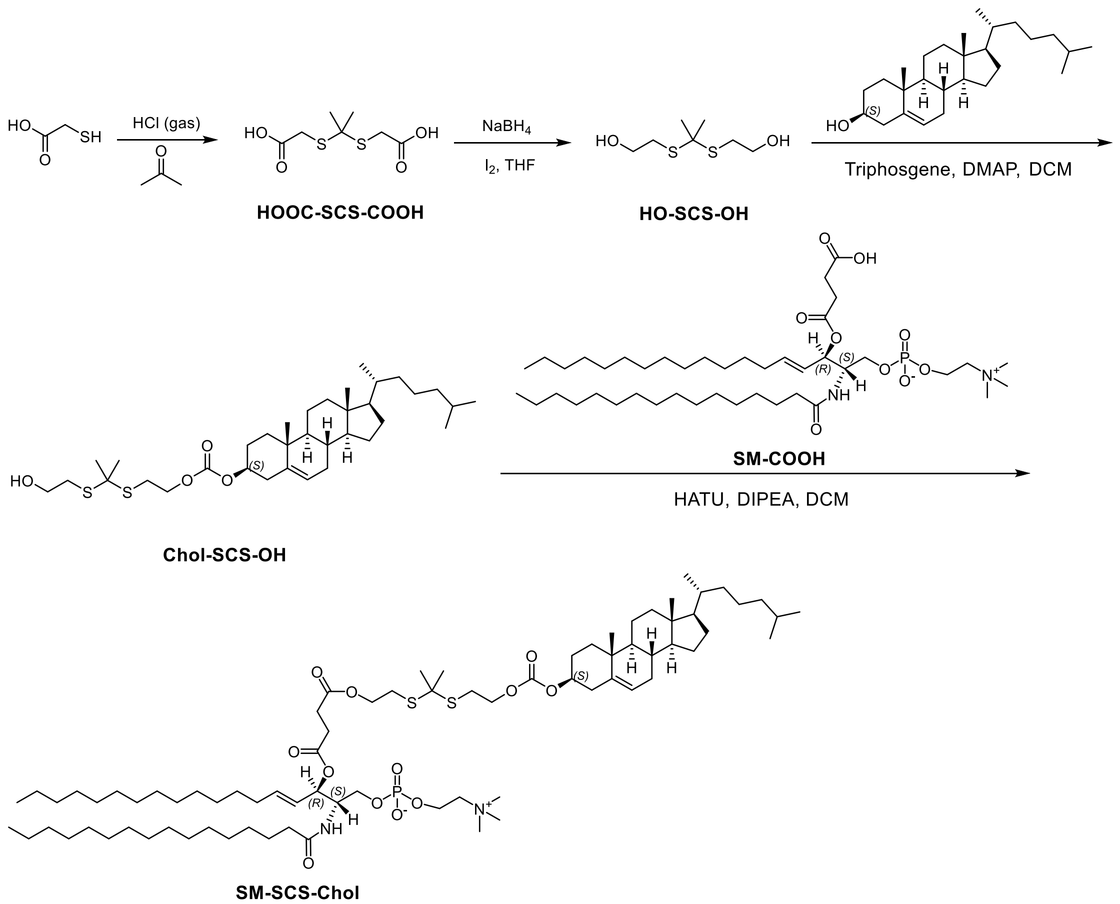

Supplementary Figure 5, Synthetic route for SM-derived Chol with thioketal bond and longer linker (SM-SCS-Chol).

2,2'-(propane-2,2-diylbis(sulfanediyl))diacetic acid (HOOC-SCS-COOH)

2-mercaptoacetic acid (92 g, 1 mol) was dissolved in 500 mL anhydrous acetone at room temperature, HCl gas was bubbled into the mixture solution for 24 h. After the completion of the reaction, the precipitation was filtered under reduced pressure, the solid was collected. White solid with 77% yield was attained. ^{1}H NMR (400 MHz, DMSO) δ 12.61 (s, 2H), 3.38 (s, 4H), 1.55 (s, 6H). ^{13}C NMR (101 MHz, DMSO) δ 171.83, 56.68, 33.26, 30.58. LC/MS (ESI): 223.0 $[M - H]^-$.

2,2'-(propane-2,2-diylbis(sulfanediyl))bis(ethan-1-ol) (HO-SCS-OH)[2]

Sodium borohydride (5.0 g, 0.132 mol), dry THF (100 mL), and HOOC-SCS-COOH (5.0 g, 0.022 mol) were charged into a 500 mL flame-dry three-necked flask fitted with magnetic stir bar and a reflux condenser. The flask was cooled in an ice bath. Subsequently, iodine (20.0 g, 0.057mol) in dry THF (100 mL) was added via an addition funnel slowly over 1 h. Then, the flask was heated to reflux for 24 h and cooled to room temperature. Methanol (50 mL) was then added cautiously until the mixture became clear. After stirring for 45 min, the solvent was removed under vacuum. The mixture was further dissolved in NaOH solution (25%, 200 mL). The resultant solution was stirred for 5 h and extracted with ethyl acetate (5 × 100 mL) and then with saturated brine. The organic layer was dried with anhydrous Na_2SO_4, the solvent was evaporated using rotary evaporator under vacuum, and the residue was purified by silica gel flash chromatography. Colorless oil with 52% yield was acquired. R_f = 0.34 (petroleum/EtOAc = 2/1). ^{1}H NMR (400 MHz, $CDCl_3$) δ 3.82 (t, J = 6.1 Hz, 4H), 2.90 (t, J = 6.1 Hz, 4H), 2.46 (s, 2H), 1.66 (s, 6H). ^{13}C NMR (101 MHz, $CDCl_3$) δ 61.29, 55.89, 33.69, 31.23. LC/MS (ESI): 197.0 $[M + H]^+$.

(3*S*,8*S*,9*S*,10*R*,13*R*,14*S*,17*R*)-10,13-dimethyl-17-((*R*)-6-methylheptan-2-yl)-2,3,4,7,8,9,10,11,12,13,14,15,16,17-tetradecahydro-1*H*-cyclopenta[*a*]phenanthren-3-yl (2-((2-((2-hydroxyethyl)thio)propan-2-yl)thio)ethyl) carbonate (Chol-SCS-OH)

DMAP (734 mg, 6 mmol, in 15 mL anhydrous DCM) was added dropwise to a solution of Chol (772 mg, 2 mmol) and triphosgene (206 mg, 0.68 mmol) in anhydrous DCM (50 mL). The reaction was stirred at room temperature for 30 min, then a solution of HO-SCS-OH (1.17 g, 6 mmol) in anhydrous THF (25 mL) was added into the mixture solution. The reaction was further stirred at room temperature for 12 h and monitored by TLC. After completion of the reaction, the mixture solution was washed with 50 mM HCl aqueous solution to remove the DMAP, and then with saturated brine. The organic layer was dried with anhydrous Na_2SO_4, the solvent was evaporated using rotary evaporator under vacuum, and the residue was purified by silica gel flash chromatography. White solid with 81% yield was acquired. R_f = 0.37 (petroleum/EtOAc = 4/1). ^{1}H NMR (400 MHz, $CDCl_3$) δ 5.42 (d, *J* = 5.1 Hz, 1H), 4.51 (tt, *J* = 10.5, 5.1 Hz, 1H), 4.30 (t, *J* = 7.1 Hz, 2H), 3.81 (t, *J* = 6.1 Hz, 2H), 2.95 (t, *J* = 7.1 Hz, 2H), 2.89 (t, *J* = 6.1 Hz, 2H), 2.47 – 2.37 (m, 2H), 2.09 – 1.81 (m, 6H), 1.76 – 1.67 (m, 1H), 1.64 (d, *J* = 8.3 Hz, 6H), 1.62 – 1.45 (m, 6H), 1.45 – 1.26 (m, 4H), 1.25 – 1.07 (m, 7H), 1.07 – 0.99 (m, 5H), 0.96 (dd, *J* = 11.5, 6.3 Hz, 4H), 0.89 (dd, *J* = 6.6, 1.7 Hz, 6H), 0.71 (s, 3H). ^{13}C NMR (101 MHz, $CDCl_3$) δ 154.34, 139.29, 123.02, 78.11, 77.22, 66.36, 61.48, 56.69, 56.29, 56.14, 50.00, 42.32, 39.72, 39.52, 38.01, 36.85, 36.54, 36.19, 35.79, 33.74, 31.90, 31.84, 31.12, 29.00, 28.23, 28.02, 27.69, 24.28, 23.83, 22.82, 22.57, 21.04, 19.27, 18.72, 11.86. LC/MS (ESI): 609.3 $[M + H]^+$.

(16*R*,17*S*)-1-(((3*S*,8*S*,9*S*,10*R*,13*R*,14*S*,17*R*)-10,13-dimethyl-17-((*R*)-6-methylheptan-2-yl)-2,3,4,7,8,9,10,11,12,13,14,15,16,17-tetradecahydro-1*H*-cyclopenta[*a*]phenanthren-3-yl)oxy)-6,6-dimethyl-1,11,14-trioxo-17-palmitamido-16-((*E*)-pentadec-1-en-1-yl)-2,10,15-trioxa-5,7-dithiaoctadecan-18-yl (2-(trimethylammonio)ethyl) phosphate (SM-SCS-Chol)

DIPEA (2 mL) was added to a solution of SM-COOH (803.0 mg, 1.0 mmol) and HATU (380 mg, 1.0 mmol) in anhydrous DCM (50 mL). The solution mixture was stirred at room temperature for 30 min. A solution of Chol-SCS-OH (609.9 mg, 1.0 mmol) in 10 mL anhydrous DCM was then added into the reaction mixture and further stirred for 12 h. After completion of the reaction, the reaction mixture was washed with 50 mM HCl aqueous solution and then with saturated brine. The organic layer was dried with anhydrous Na_2SO_4, the solvent was evaporated using rotary evaporator under vacuum, and the residue was purified by silica gel flash chromatography with $CHCl_3$/EtOH/H_2O (v/v/v, 300/200/36) as the eluting solvent. White solid with 82% yield was attained. R_f = 0.33 ($CHCl_3$/EtOH/H_2O = 300/200/36). ^{1}H NMR (400 MHz, $CDCl_3$) δ 7.33 (d, *J* = 8.1 Hz, 1H), 5.75 – 5.65 (m, 1H), 5.39 (dd, *J* = 14.4, 6.6 Hz, 2H), 5.28 (t, *J* = 7.5 Hz, 1H), 4.45 (td, *J* = 10.8, 5.0 Hz, 1H), 4.29 (s, 3H), 4.22 (dd, *J* = 15.3, 8.1 Hz, 4H), 3.88 (s, 2H), 3.78 (s, 2H), 3.32 (s, 9H), 2.89 – 2.81 (m, 4H), 2.60 (d, *J* = 15.7 Hz, 4H), 2.41 – 2.32 (m, 2H), 2.12 (s, 2H), 2.06 – 1.70 (m, 8H), 1.61 (d, *J* = 21.1 Hz, 8H), 1.56 – 1.40 (m, 8H), 1.26 (dd, *J* = 25.8, 7.7 Hz, 55H), 1.10 (ddd, *J* = 17.4, 14.5, 6.6 Hz, 8H), 0.99 (s, 6H), 0.86 (ddd, *J* = 8.9, 8.2, 4.1 Hz, 16H), 0.66 (s, 3H). ^{13}C NMR (101 MHz, $CDCl_3$) δ 173.42, 172.09, 171.23, 154.23, 139.22, 137.30, 124.43, 122.98, 78.01, 74.06, 66.43, 66.32, 63.97, 63.94, 63.71, 59.28, 59.25, 56.65, 56.48, 56.11, 54.46, 51.27, 51.23, 49.96, 42.27, 39.68, 39.48, 37.98, 36.82, 36.74, 36.49, 36.15, 35.75, 32.36, 31.91, 31.86, 31.80, 30.95, 29.74, 29.73, 29.69, 29.67, 29.64, 29.59, 29.55, 29.40, 29.36, 28.99, 28.94, 28.19, 27.97, 27.65, 25.92, 24.24, 23.80, 22.79, 22.67, 22.53, 21.01, 19.23, 18.68, 14.10, 11.82. HRMS (ESI) m/z $[M + Na]^+$ for $C_{78}H_{141}N_2O_{12}PS_2$ calculated 1415.9501, found 1415.9555.

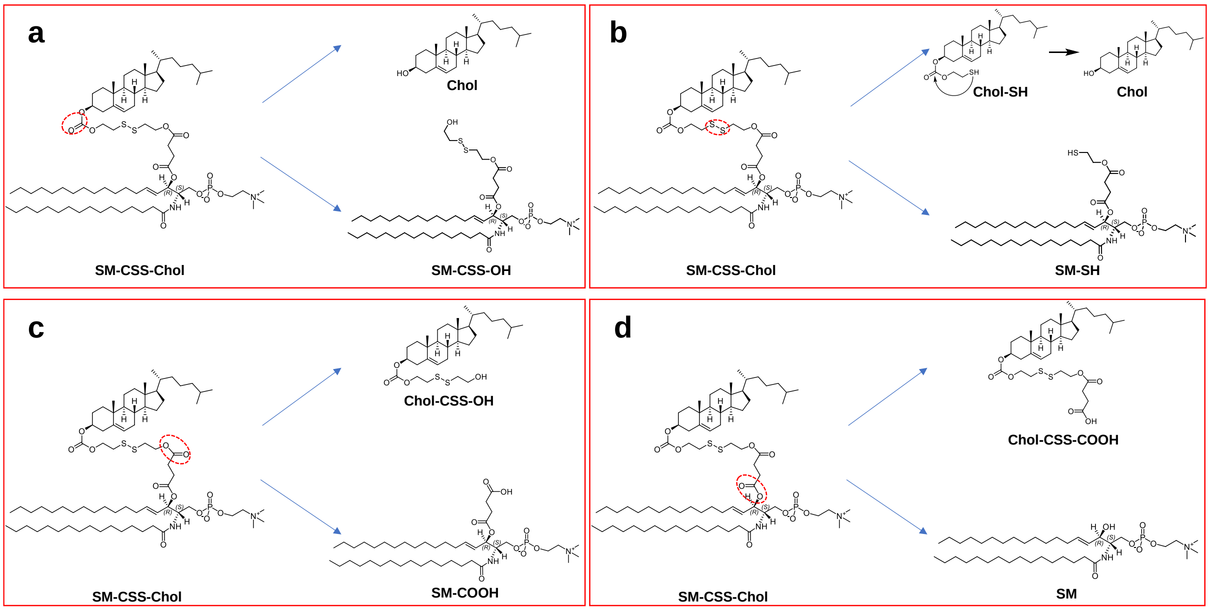

Supplementary Figure 6, The possible intermediates released from SM-CSS-Chol by breaking the carbonate ester (**a**), disulfide (**b**), the ester (**c**, in the middle of the linker) and the ester (**d**, connected to SM) bond.

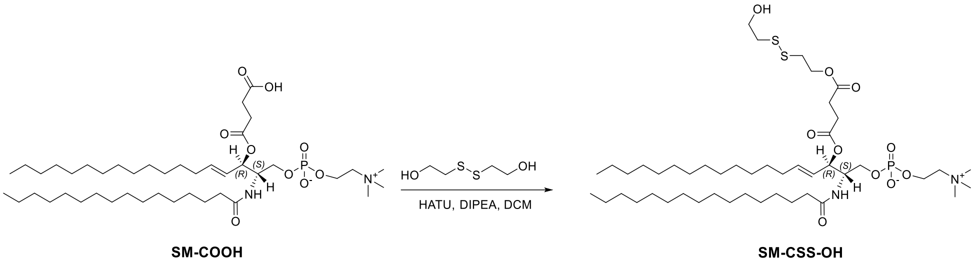

Supplementary Figure 7, Synthetic route for SM-CSS-OH

(2*S*,3*R*,*E*)-3-((4-(2-((2-hydroxyethyl)disulfaneyl)ethoxy)-4-oxobutanoyl)oxy)-2-palmitamidooctadec-4-en-1-yl (2-(trimethylammonio)ethyl) phosphate (SM-CSS-OH)

DIPEA (2 mL) was added to a solution of SM-COOH (803.0 mg, 1.0 mmol) and HATU (570 mg, 1.5 mmol) in anhydrous DCM (50 mL). The solution mixture was stirred at room temperature for 30 min. A solution of 2, 2'-dithiodiethanol (616 mg, 4.0 mmol) in 10 mL anhydrous THF was then added into the reaction mixture and further stirred for 12 h. After completion of the reaction, the reaction mixture was washed with 50 mM HCl aqueous solution and then with saturated brine. The organic layer was dried with anhydrous Na_2SO_4,

the solvent was evaporated using rotary evaporator under vacuum, and the residue was purified by silica gel flash chromatography with $CHCl_3$/EtOH/H_2O (v/v/v, 300/200/36) as the eluting solvent. White solid with 72% yield was attained. R_f = 0.29 ($CHCl_3$/EtOH/H_2O = 300/200/36). ^{1}H NMR (500 MHz, $CDCl_3$) δ 7.18 (d, *J* = 9.0 Hz, 1H), 5.79 – 5.67 (m, 1H), 5.39 (dd, *J* = 15.0, 7.7 Hz, 1H), 5.32 (d, *J* = 7.1 Hz, 1H), 4.33 (dd, *J* = 25.8, 14.6 Hz, 5H), 3.89 (s, 2H), 3.83 – 3.68 (m, 4H), 3.30 (s, 9H), 2.91 (dd, *J* = 13.4, 5.2 Hz, 4H), 2.71 – 2.53 (m, 4H), 2.14 (t, *J* = 7.4 Hz, 2H), 1.98 (dd, *J* = 13.5, 6.5 Hz, 2H), 1.54 (d, *J* = 5.2 Hz, 2H), 1.24 (s, 46H), 1.06 – 0.63 (m, 6H). ^{13}C NMR (126 MHz, $CDCl_3$) δ 173.46 (s), 172.48 (s), 171.18 (s), 137.61 (s), 124.60 (s), 74.01 (s), 66.42 (s), 64.00 (s), 62.73 (s), 60.11 (s), 59.35 (s), 54.49 (s), 51.20 (s), 42.30 (s), 37.12 (s), 36.76 (s), 32.36 (s), 31.92 (s), 30.12 – 29.27 (m), 29.00 (s), 25.92 (s), 22.67 (s), 14.08 (s). HRMS (ESI) m/z $[M + H]^+$ for $C_{47}H_{91}N_2O_{10}PS_2$ calculated 939.59255, found 939.59291.

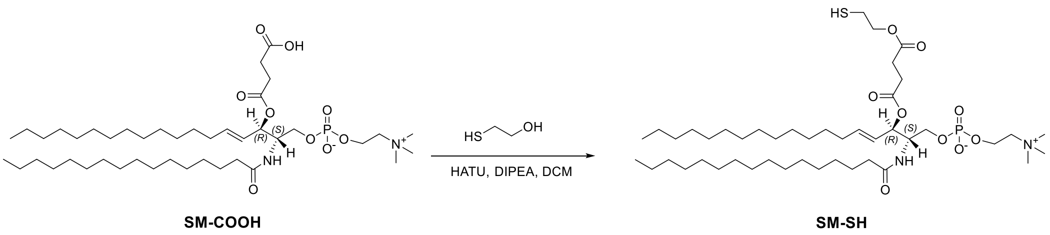

Supplementary Figure 8, The synthesis of SM-SH.

(2*S*,3*R*,*E*)-3-((4-(2-mercaptoethoxy)-4-oxobutanoyl)oxy)-2-palmitamidooctadec-4-en-1-yl (2-(trimethylammonio)ethyl) phosphate (SM-SH)

DIPEA (2 mL) was added to a solution of SM-COOH (803.0 mg, 1.0 mmol) and HATU (570 mg, 1.5 mmol) in anhydrous DCM (50 mL). The solution mixture was stirred at room temperature for 30 min. A solution of 2-mercaptoethan-1-ol (312 mg, 4.0 mmol) in 10 mL anhydrous THF was then added into the reaction mixture and further stirred for 12 h. After completion of the reaction, the reaction mixture was washed with 50 mM HCl aqueous

solution and then with saturated brine. The organic layer was dried with anhydrous Na_2SO_4, the solvent was evaporated using rotary evaporator under vacuum, and the residue was purified by silica gel flash chromatography with $CHCl_3/EtOH/H_2O$ (v/v/v, 300/200/36) as the eluting solvent. White solid with 41% yield was attained. R_f = 0.31 ($CHCl_3/EtOH/H_2O$ = 300/200/36). ^1H NMR (500 MHz, $CDCl_3$) δ 5.78 – 5.64 (m, 1H), 5.40 (dd, *J* = 14.6, 7.7 Hz, 1H), 5.37 – 5.25 (m, 1H), 4.47 – 4.14 (m, 5H), 3.97 – 3.87 (m, 2H), 3.80 (s, 2H), 3.34 (s, 9H), 2.92 (dd, *J* = 12.4, 6.2 Hz, 1H), 2.73 (ddd, *J* = 11.8, 9.6, 6.3 Hz, 1H), 2.67 – 2.56 (m, 4H), 2.15 (dd, *J* = 14.3, 6.8 Hz, 2H), 1.97 (d, *J* = 5.8 Hz, 2H), 1.54 (s, 2H), 1.27 (dd, *J* = 21.7, 6.4 Hz, 46H), 0.88 (t, *J* = 6.9 Hz, 6H). ^{13}C NMR (126 MHz, $CDCl_3$) δ 173.73, 172.49, 171.41, 137.44, 124.47, 73.77, 66.20, 66.08, 64.17, 62.47, 59.37, 54.27, 51.19, 37.36, 36.64, 32.44, 31.94, 29.89, 29.83, 29.77, 29.70, 29.62, 29.51, 29.46, 29.39, 29.03, 28.98, 26.07, 22.69, 14.10. HRMS (ESI) m/z $[M + H]^+$ for $C_{45}H_{87}N_2O_9PS$ calculated 863.59427, found 863.59424.

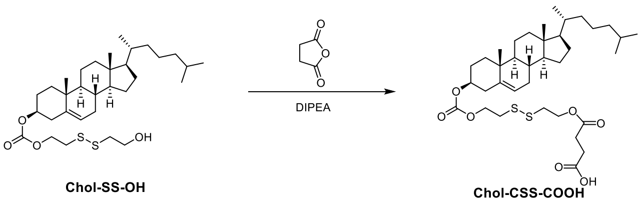

Supplementary Figure 9, Synthetic route for Chol-CSS-COOH.

4-(2-((2-(((((3*S*,8*S*,9*S*,10*R*,13*R*,14*S*,17*R*)-10,13-dimethyl-17-((*R*)-6-methylheptan-2-yl)-2,3,4,7,8,9,10,11,12,13,14,15,16,17-tetradecahydro-1*H*-cyclopenta[a]phenanthren-3-yl)oxy)carbonyl)oxy)ethyl)disulfaneyl)ethoxy)-4-oxobutanoic acid (Chol-SS-COOH)

DIPEA (2 mL) was added to a solution of Chol-SS-OH (0.566 g, 1.0 mmol) and succinic anhydride (0.12 g, 1.2 mmol) in anhydrous $CHCl_3$ (30 mL). The solution was stirred at room temperature for 12 h and monitored by TLC. After completion of the reaction, CH_3OH (30 mL) was added into the mixture solution, the reaction was further stirred at room

temperature for 12 h. The solvent was evaporated using rotary evaporator under vacuum, and the residue was purified by silica gel flash chromatography with CH_2Cl_2/MeOH (v/v, 20/1) as the elution solvent. White solid with 78% yield was garnered. R_f = 0.26 (CH_2Cl_2/MeOH = 20/1). ^{1}H NMR (500 MHz, $CDCl_3$) δ 5.38 (d, *J* = 4.3 Hz, 1H), 4.46 (tt, *J* = 10.7, 5.1 Hz, 1H), 4.35 (t, *J* = 6.5 Hz, 4H), 2.92 (q, *J* = 6.4 Hz, 4H), 2.66 (dd, *J* = 13.3, 5.4 Hz, 4H), 2.42 – 2.33 (m, 2H), 1.96 (dd, *J* = 33.0, 13.1 Hz, 3H), 1.86 (d, *J* = 13.4 Hz, 1H), 1.83 – 1.77 (m, 1H), 1.65 (dd, *J* = 18.5, 7.9 Hz, 1H), 1.60 – 1.37 (m, 7H), 1.32 (dt, *J* = 25.3, 12.5 Hz, 3H), 1.26 – 1.16 (m, 2H), 1.15 – 1.02 (m, 7H), 1.01 – 0.91 (m, 6H), 0.89 (d, *J* = 6.4 Hz, 3H), 0.87 – 0.81 (m, 6H), 0.66 (s, 3H). ^{13}C NMR (126 MHz, $CDCl_3$) δ 177.12, 171.88, 154.34, 139.27, 123.06, 78.24, 65.34, 62.58, 60.86, 56.70, 56.14, 50.00, 42.32, 39.72, 39.52, 37.99, 37.21, 37.06, 36.84, 36.54, 36.19, 35.79, 31.91, 31.84, 28.90, 28.82, 28.75, 28.23, 28.02, 27.67, 24.28, 23.83, 22.82, 22.57, 21.05, 19.27, 18.72, 14.15, 11.87. HRMS (ESI) m/z $[M + NH_4]^+$ for $C_{36}H_{58}O_7S_2$ calculated 684.39622, found 684.39603.

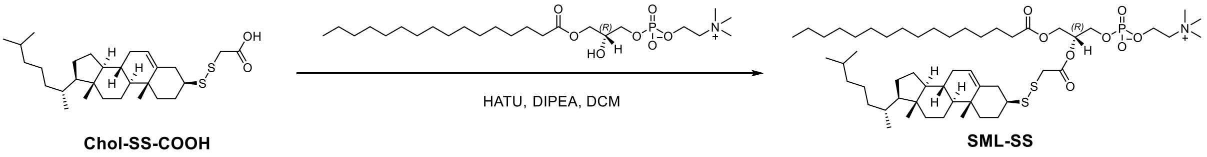

Supplementary Figure 10, Synthetic route for SML-SS.

(*R*)-2-(2-(((3*S*,8*S*,9*S*,10*R*,13*R*,14*S*,17*R*)-10,13-dimethyl-17-((*R*)-6-methylheptan-2-yl)-2,3,4,7,8,9,10,11,12,13,14,15,16,17-tetradecahydro-1*H*-cyclopenta[a]phenanthren-3-yl)disulfaneyl)acetoxy)-3-(palmitoyloxy)propyl (2-(trimethylammonio)ethyl) phosphate (SML-SS)

DIPEA (2 mL) was added to a solution of Chol-SS-COOH (492.8 mg, 1.0 mmol) and HATU (570 mg, 1.5 mmol) in anhydrous DCM (20 mL). The solution mixture was stirred at room temperature for 30 min. A solution of (*R*)-2-Hydroxy-3-(palmitoyloxy)propyl (2-(trimethylammonio)ethyl) phosphate (495.6 mg, 1.0 mmol) in 10 mL anhydrous DCM was

then added into the reaction mixture and further stirred for 12 h. After completion of the reaction, the reaction mixture was washed with 50 mM HCl aqueous solution and then with saturated brine. The organic layer was dried with anhydrous Na_2SO_4, the solvent was evaporated using rotary evaporator under vacuum, and the residue was purified by silica gel flash chromatography with $CHCl_3$/EtOH/H_2O (v/v/v, 300/200/36) as the eluting solvent. White solid with 71% yield was attained. R_f = 0.31 ($CHCl_3$/EtOH/H_2O = 300/200/36). ^{1}H NMR (400 MHz, $CDCl_3$) δ 5.28 (s, 1H), 5.28 (s, 1H), 4.37 (s, 2H), 4.19 (s, 1H), 4.01 (s, 2H), 3.85 (s, 2H), 3.72 (d, J = 6.8 Hz, 1H), 3.58 (s, 1H), 3.37 (s, 9H), 2.30 (s, 2H), 2.17 (d, J = 13.1 Hz, 1H), 1.98 (d, J = 9.8 Hz, 2H), 1.85 (d, J = 20.4 Hz, 2H), 1.70 (d, J = 8.3 Hz, 1H), 1.53 (d, J = 29.6 Hz, 7H), 1.26 (s, 28H), 1.18 – 1.06 (m, 6H), 1.00 (s, 5H), 0.88 (d, J = 9.2 Hz, 14H), 0.68 (s, 3H). ^{13}C NMR (101 MHz, $CDCl_3$) δ 173.60, 169.35, 151.90, 116.63, 77.23, 56.27, 55.95, 54.56, 54.40, 48.13, 42.51, 42.00, 39.83, 39.50, 37.73, 36.16, 35.84, 34.16, 32.95, 32.68, 32.12, 31.96, 29.81, 29.72, 29.47, 29.42, 29.33, 28.23, 28.01, 24.90, 24.26, 24.13, 23.96, 22.83, 22.72, 22.57, 21.51, 18.99, 18.66, 14.15, 11.96. HRMS (ESI) m/z [M + H]$^+$ for $C_{53}H_{97}NO_8PS_2$ calculated 970.63877, found 970.63883.

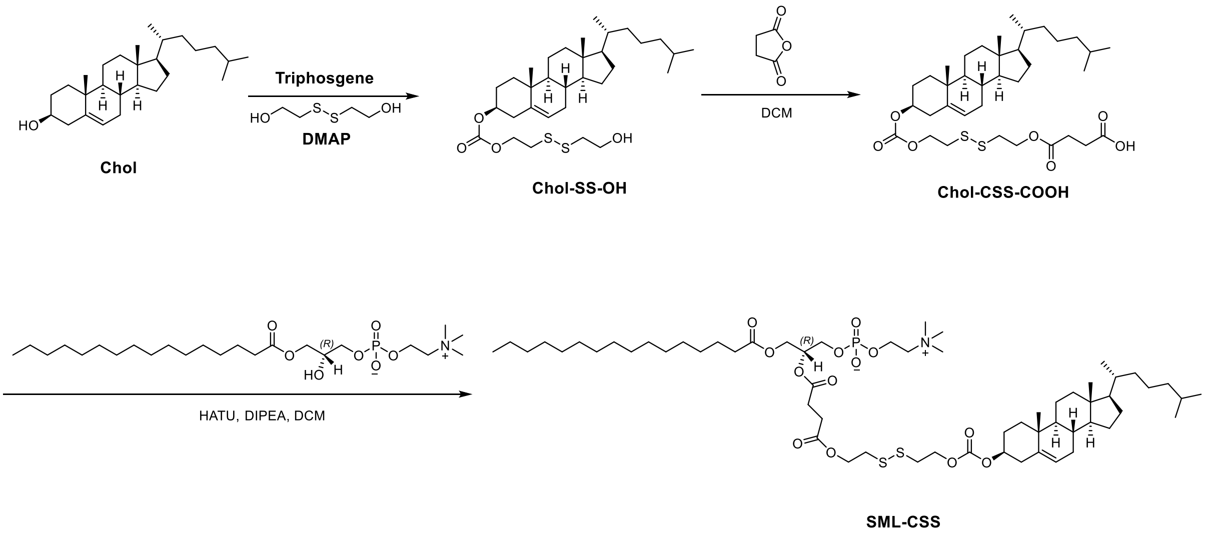

Supplementary Figure 11, Synthetic route for SML-CSS.

(*R*)-1-(((3*S*,8*S*,9*S*,10*R*,13*R*,14*S*,17*R*)-10,13-dimethyl-17-((*R*)-6-methylheptan-2-yl)-2,3,4,7,8,9,10,11,12,13,14,15,16,17-tetradecahydro-1*H*-cyclopenta[a]phenanthren-3-yl)oxy)-1,10,13-trioxo-15-((palmitoyloxy)methyl)-2,9,14-trioxa-5,6-dithiahexadecan-16-yl (2-(trimethylammonio)ethyl) phosphate (SML-CSS)

DIPEA (2 mL) was added to a solution of Chol-CSS-COOH (666.9 mg, 1.0 mmol) and HATU (570 mg, 1.5 mmol) in anhydrous DCM (20 mL). The solution mixture was stirred at room temperature for 30 min. A solution of (*R*)-2-Hydroxy-3-(palmitoyloxy)propyl (2-(trimethylammonio)ethyl) phosphate (495.6 mg, 1.0 mmol) in 10 mL anhydrous DCM was then added into the reaction mixture and further stirred for 12 h. After completion of the reaction, the reaction mixture was washed with 50 mM HCl aqueous solution and then with saturated brine. The organic layer was dried with anhydrous Na_2SO_4, the solvent was evaporated using rotary evaporator under vacuum, and the residue was purified by silica gel flash chromatography with $CHCl_3$/EtOH/H_2O (v/v/v, 300/200/36) as the eluting solvent. White solid with 67% yield was attained. R_f = 0.32 ($CHCl_3$/EtOH/H_2O = 300/200/36). ^{1}H NMR (400 MHz, $CDCl_3$) δ 5.40 (s, 1H), 5.21 (s, 1H), 4.47 (td, *J* = 10.9, 5.7 Hz, 1H), 4.42 – 4.22 (m, 7H), 4.15 (dd, *J* = 11.5, 6.9 Hz, 1H), 3.97 (s, 2H), 3.83 (s, 2H), 3.38 (s, 9H), 2.98 – 2.89 (m, 4H), 2.64 (s, 4H), 2.38 (d, *J* = 10.4 Hz, 2H), 2.29 (t, *J* = 7.1 Hz, 2H), 2.06 – 1.75 (m, 4H), 1.71 – 1.40 (m, 8H), 1.38 – 1.31 (m, 3H), 1.31 – 1.19 (m, 28H), 1.12 (dd, *J* = 19.8, 10.4 Hz, 6H), 1.00 (d, *J* = 11.5 Hz, 5H), 0.96 – 0.83 (m, 12H), 0.68 (s, 3H). ^{13}C NMR (101 MHz, $CDCl_3$). NMR (101 MHz,) δ 174.58, 173.08, 172.72, 155.24, 140.23, 124.05, 79.17, 78.26, 66.27, 63.70, 63.53, 57.67, 57.13, 55.41, 50.97, 43.30, 40.70, 40.51, 39.00, 37.99, 37.93, 37.83, 37.53, 37.18, 36.79, 35.06, 32.94, 32.90, 32.83, 30.74, 30.69, 30.57, 30.38, 30.21, 30.02, 29.84, 29.23, 29.01, 28.67, 25.86, 25.28, 24.83, 23.82, 23.70, 23.56, 22.04, 20.27, 19.71, 15.15, 12.86. HRMS (ESI) m/z $[M + H]^+$ for $C_{60}H_{107}NO_{13}PS_2$ calculated 1144.69160, found 1144.69183.

Calcein release kinetics of Calcein/Lipo-CSS-Chol

Calcein/Lipo-CSS-Chol was prepared as described above. PBS (pH = 7.4, GSH = 10 mM) containing 0.5% (w/v) Tween 80 was used as the release medium for the drug release kinetics study of Calcein/Lipo-CSS-Chol in vitro. Briefly, two mL of Calcein/Lipo-CSS-Chol were transferred into dialysis tubes (MWCO = 12 kDa, Spectrum Laboratories), and then suspended in 200 mL release medium inside a beaker shielded with aluminum foil. The temperature of the release medium was kept at 37 °C and stirred at 100 rpm. Before incubation (0 h) and at indicated time points, 5 and 100 μL of solution were taken from the dialysis tubes and dialysate, respectively. The concentration of Calcein remaining in the dialysis tubes and dialysate were measured by fluorescence signal (excitation: 494 nm; emission: 517 nm) via a SpectraMax M3 reader (SoftMax Pro (v. 7.1.0), Molecular Devices).

The release intermediates from Lipo-SM-CSS-Chol

Lipo-SM-CSS-Chol (500 μg SM-CSS-Chol/mL, n = 3) were incubated with or without GSH (10 mM) in PBS at 37 °C for indicated time. Incubation mixtures were diluted by 50 μL methanol per 100 μL samples. A 200 μL of methyl-tert-butyl ether (MTBE) was added and the mixture was vortexed for 10 min. After separation, the organic phase was analyzed by TLC with the possible intermediates as reference compounds (**Supplementary Fig. 23**). The newly generated spot was isolated and further analyzed by HRMS via a LTQ Orbitrap Velos mass spectrometer with an ESI source (Thermo Scientific).

The stability study of Lipo-SM-CSS-Chol by LC-MS/MS

Lipo-SM-CSS-Chol (500 µg SM-CSS-Chol/mL, n = 3) were incubated with or without GSH (10 mM) in PBS at 37 °C for indicated time. Incubation mixtures were extracted with a one-step liquid-liquid extraction (LLE) method optimized from a protocol for lipidomics[3]. Briefly, each 10 µL incubation mixture was mixed with 10 µL internal standard (IS; SM-Glycine-Chol, 100 µg/mL) and 100 µL methanol, and the mixture was vortexed for 10 sec., A 3 mL of methyl-tert-butyl ether (MTBE) was added and the mixture was vortexed for 30 sec. Then, 150 µL water was added and the mixture was vortexed for another 3 min, followed by centrifugation at 1800 g for 10min. The organic layer was collected and evaporated under nitrogen stream at 40 ℃. The residue was reconstituted into 1 mL methanol and the solution was centrifuged again at 20000 g for 5 min. A 2 µL of the supernatant was used for /LC-MS/MS analysis. SM-CSS-Chol was detected using LC-MS/MS.

The LC-MS/MS system consisted of an Agilent 1290 UPLC system (Agilent Technologies, Santa Clara, CA) and a Sciex Qtrap6500$^+$ Mass Spectrometer (AB SCIEX, Framingham, MA). Analytes were separated on an EclipsePlus C18 column (2.1 × 50 mm, 1.8 µm, Agilent) at temperature of 55 ℃, with mobile phase A containing 0.1% formic acid (v/v) and 5 mM ammonium formate in water: acetonitrile (50:50) and mobile phase B containing 0.1% formic acid (v/v) and 5 mM ammonium formate in isopropanol: acetonitrile (90:10). Elution was at a flow rate of 0.3 mL/min as follows: 10% B (0-0.5 min), 10% B→95% B (0.5-2 min), 95% B (2-7.5 min), 95% B→1% B (7.5-7.6 min), 10% B (7.6-10 min). The MS was operated in the positive ion mode, using electrospray ionization. The ion spray voltage and temperature were set at 5500 V and 550 °C, respectively. Curtain gas, ion source gas1 and ion source gas2 were set at 25, 40, 10 psi, respectively. SM-CSS-Chol and IS were detected using Multiple Reaction Monitoring (MRM), with a dwell time of 200 msec per transition, at m/z 1351.5/685.4 and 1228.5/685.3, respectively. Retention times for SAH and IS were 3.8 and 3.6 min, respectively. For quantitative analysis of SM-CSS-Chol, standards

(1 to 500 µg/mL in 10 µL water), along with 10 µL IS (at 100 µg/mL in methanol), were added to 10 µL of blank matrix to construct the calibration curve.

Maximum tolerated dose (MTD)

Groups of C57BL/6 mice (n = 6 mice) were administered intravenously with free vincristine (2 and 3 mg VCR/kg, formulated in 5% dextrose), VCR/Lipo-PChcPC, VCR/Lipo-SM/Chol and VCR/Lipo-SM-CSS-Chol (3, 4 and 5 mg VCR/kg). Changes in body weight and survival of mice were monitored for two weeks. The MTD was defined as the dose that causes neither mouse death due to the toxicity nor greater than 15% of body weight loss or other remarkable changes in the general appearance within the entire period of the experiments. On day 14 post drug injection, blood was withdrawn by cardiac puncture. Blood was collected in lithium heparin tubes (BD Microtainer™) followed by centrifuging at 2,000 g for 10 minutes in a refrigerated centrifuge. The supernatant (serum) was sent to University Animal Care Pathology Services Core at UArizona for serum chemistry analysis (Liasys 330). The whole blood in dipotassium EDTA tube (BD Microtainer™) were used for leukocytes, erythrocytes, and thrombocytes analysis (Hemavet 950FS).

Chol exchange

The unilamellar Lipo were prepared by extrusion and were confirmed by cryo-EM. The donor Lipo consisted of eq. 40 mol % Chol, 50 mol % SM and 10 mol % negatively charged corresponding 1,2-dioleoyl-sn-glycero-3-phospho-(1'-rac-glycerol) (sodium salt) (PG). SMLs (PChcPC, PChemsPC, OChemsPC, DChemsPC) liposomes consisted of eq. 40 mol % Chol, 50 mol % 1,2-dipalmitoyl-sn-glycero-3-phosphocholine (DPPC) and 10 mol % negatively charged corresponding 1,2-dioleoyl-sn-glycero-3-phospho-(1'-rac-glycerol) (sodium salt) (PG))[4]. For acceptor Lipo, we used the neutral POPC Lipo at 10-fold molar excess (without

Chol). To examine the Chol exchange, 1 mL of 10 mM donor Lipo and 1 mL of 100 mM acceptor Lipo (10-fold) were first warmed at 37 °C, which later were mixed together and continued to be incubated at 37 °C. An aliquot of 250 µL sample solutions were collected at the indicated time points and loaded into an anion exchange column (ca. 2 cm in length, Q-Sepharose XL). In order to avoid the nonspecific binding of the neutral Lipo, the column was treated with 0.1 mL of POPC (10 mM) prior to loading the samples and was eluted with 1 mL buffer (10 mM NaCl, 10 mM HEPES buffer, pH = 7.4). To determine the amount of Chol exchanged, the eluate was collected, lyophilized and then quantified using the reported Chol assay[5]. In brief, the lyophilizate was dissolved in DI water (100 µL) and 50 µL was then transferred to a glass tube, to which 5 mL of Chol assay reagent comprised of 339 mg ferric perchlorate hexahydrate in 300 mL of ethyl acetate mixed with 200 mL concentrated sulfuric acid at 4 °C, was then added. the resultant mixture was heated for 90 s at 100 °C and subsequently immersed into ice-water immediately. The Chol concentration was determined based on the established standard curve by measuring the absorbance at 610 nm (**Supplementary Fig. 26**).

Pharmacokinetics and biodistribution of VCR/Lipo

Free VCR (2 mg/kg), or various VCR/Lipo (2 mg VCR/kg) were intravenously injected to orthotopic MC38 tumour bearing mice (n = 3 mice, tumour weight: ~400 mg) via tail vein. At 0.083, 0.5, 2, 8, 24 and 48 h post drug administration, blood was withdrawn; and plasma was obtained using a plasma tube (BD Microtainer) and then digested in methanol prior to HPLC measurement for the VCR. At 48 h, tumour tissues and major organs (heart, liver, spleen, lung, kidney) were collected and homogenized in acidified methanol (0.075 M HCl, 900 µL/100 mg tissue) before HPLC drug content analysis. The various pharmacokinetic parameters were assessed using PKSolver software (version 2.0)[6].

Preparation of MU-P/DiD/Lipo[7]

Phospholipid, DiD, Chol and DSPE-PEG$_{2K}$ at the indicated molar ratio in **Supplementary Figure 37a** were dissolved in ethanol by a 100 mL round bottom glass flask. The organic solvent was evaporated under reduced pressure by using a rotatory evaporator (RV 10 digital, IKA®) to generate a thin film, and then further dried under ultra-high vacuum (MaximaDry, Fisherbrand) for 0.5 h. The lipid film was hydrated by 300 mM MU-P in HBS buffer (10 mM HEPES, 140 mM NaCl, pH 7.4) at 60 °C for 30 min, and then sonicated under an ice bath for 12 min by using a pulse 3/2 s on/off at a power output of 60 W (VCX130, Sonics & Materials Inc). The nanovesicles were further purified by running through a PD-10 column to remove the unencapsulated MU-P with PBS as the eluent. The size, zeta potential, PDI and MU-P content in the liposomes were determined by DLS, HPLC respectively. The MU-P drug loading capacity [DLC, equation (4)] and drug loading efficiency [DLE, equation (5)] were calculated as below:

$$\text{equation (4)} = \frac{\text{weight of encapsulated drug}}{\text{weight of (total lipids + encapsulated drug)}} \times 100\%$$

$$\text{equation (5)} = \frac{\text{weight of encapsulated drug}}{\text{weight of input drug}} \times 100\%$$

Pharmacokinetics, biodistribution, and release rate in tumours of MU-P/DiD/Lipo[7]

Free MU-P (20 mg/kg), DiD (0.25 mg/kg) or various MU-P/DiD/Lipo (20 mg MU-P/kg, 0.25 mg DiD/kg) were intravenously injected to orthotopic KPC-Luc tumour bearing mice (n = 3 mice, tumour weight: ~400 mg) via tail vein. At 0.083, 0.5, 2, 8, 24 and 48 h post injection, blood was withdrawn; and plasma was obtained using a plasma tube (BD Microtainer) and then diluted with a 1:100 of phosphatase inhibitor cocktail (#78420, Thermo

Scientific). 5 μL of plasma was added to 1 mL of HBS buffer (10 mM HEPES, 140 mM NaCl, pH 7.4) along with 5 μL of a 15% $C_{12}E_{10}$ (#P9769, Sigma) solution. The fluorescence signal of MU-P (excitation: 320 nm; emission: 385 nm), MU (excitation: 360 nm; emission: 449 nm) and DiD (excitation 644 nm, emission 664 nm) were detected using a SpectraMax M3 reader (SoftMax Pro (v. 7.1.0), Molecular Devices, **Supplementary Fig. 43**). The various pharmacokinetic parameters were assessed using PKSolver software (version 2.0) through fitting to a one-compartment model[6]. At 48 h, tumour tissues and major organs (liver, spleen, kidney) were collected and homogenized in acidified methanol (50% Methanol, 0.1%TFA, 1:100 dilution of Phosphatase Inhibitor Cocktail 2, 1 μg/mL 7-hydroxycoumarin as an internal standard, 900 μL/100 mg tissue) before HPLC drug content analysis. The percent injected dose in biodistribution was calculated by fitting peak areas to standard curves of MU, MU-P, and MU-G (**Supplementary Fig. 44**), and the the release ratio [equation (6)] was calculated as below:

$$\text{equation (6)} = \frac{\mathrm{MU} + \mathrm{MU} - \mathrm{G}}{\mathrm{MU} + \mathrm{MU} - \mathrm{G} + \mathrm{MU} - \mathrm{P}} \times 100\%$$

Orthotopic metastatic KPC-Luc (luciferase-expressing) PDAC tumour model

To establish the orthotopic KPC-Luc PDAC model B6129SF1/J mice were anesthetized by isoflurane[8]. The hair/fur around abdominal area of mice was removed by a shaver. Then three alternating scrubs of betadine/povidone iodine followed by 70% ethanol were applied to the surgical area. A s.c. injection of buprenorphine SR (1.0 mg/kg) was administered to mice prior to surgery. Afterwards, an abdominal incision (~0.5-0.7 cm) was created using a sterile disposable scalpel; and the pancreas was exteriorized. 2 × 10^6 of KPC-Luc cells in 50 μL of DMEM medium with Matrigel (Corning, Discovery labware Inc.) (3/1, v/v) were inoculated into the pancreatic tail using a 26-gauge needle (BD precisionGlide™). Afterwards, pancreas

was placed back into the peritoneal cavity after sterilizing the injection site with 70% ethanol to kill any cancer cells that may have leaked out. Then the abdominal wall and skin were closed with size 6-0 absorbable sutures (PDS II, Ethicon) and size 5-0 non-absorbable sutures (PROLENE, Ethicon), respectively. Surgical glue was applied to assure good apposition of skin. During and after surgery, animals were placed on the heating pad and were closely monitored until ambulatory and were then returned to a clean cage. Tumour burden of a whole mouse body was determined by bioluminescence radiance intensity using Lago optical imaging after mice were intraperitoneally injected with 150 mg/kg *D*-Luciferin (GoldBio, MO, USA). 11 days following cancer cells inoculation into pancreas, orthotopic KPC-Luc tumour-bearing mice (n = 6 mice; primary tumour weight: ∼400 mg) were randomly allocated into 6 groups. Mice were then intravenously administered with 5% dextrose (vehicle control), Onivyde or various IRI/Lipo at 40 mg IRI/kg every 3 days for a total of 3 doses. The whole body tumour burden was monitored by Lago optical imager on day 11, 18 and 25 and quantified as bioluminescence radiance intensity (p/sec/cm^2/sr) using Aura 3.2.0 imaging software. On day 25, following injection of *D*-Luciferin, mice were dissected, and gastrointestinal tract and other major organs (heart, liver, spleen, lung, kidneys, stomach, small and large intestines, caecum and rectum) were quickly obtained and then subject to photographing and *ex vivo* Lago imaging to investigate the tumour metastasis.

Pharmacokinetics and biodistribution of IRI/Lipo

IRI/Lipo (40 mg IRI/kg) were intravenously injected to orthotopic KPC-Luc PDAC tumour bearing mice (n = 3 mice, tumour weight: ~400 mg) via tail vein. At 0.083, 0.5, 2, 8, 24 and 48 h post drug administration, blood was withdrawn; and plasma was obtained using a plasma tube (BD Microtainer) and then digested in methanol prior to HPLC measurement for the IRI. At 48 h, tumour tissues and major organs (heart, liver, spleen, lung, kidney) were

collected and homogenized in acidified methanol (0.075 M HCl, 900 μL/100 mg tissue) before HPLC drug content analysis. The various pharmacokinetic parameters were assessed using PKSolver software (version 2.0)[6].

Evaluation of the rigidity of Lipo-SM-Chol

The rigidity of Lipo-SM-Chol was determined ed using atomic force microscopy (AFM). An aqueous solution of Lipo-SM-Chol was placed on mica surface modified with 3-aminopropyltriethoxysilane (A043925G, Fisher Scientific) and N, N-diisopropylethylamine (AC367840250, Fisher Scientific) for 30 min, and then washed with distilled water. Subsequently, the surface containing Lipo-SM-Chol was collected using AC Mode with Blue Drive using a Bruker DNPS probe (Santa Barbara, CA) on an Oxford Asylum Instruments Cypher scanning probe instrument (Santa Barbara, CA). Images were analyzed (flattened with Magic Mask) using AR 16.23.224 running inside Igor Pro 6.38B01.[9,10] The height value represents the histogram mode using this apparatus's software. Separately, the nanoparticle size (P) was measured using DLS before the Lipo were adsorbed onto the substrate surface. The height/diameter (H/D) ratio [equation (7)] was used to determine the rigidity of the liposomal membrane.[10]

$$\text{equation (7)} = \frac{\text{Height of Lipo(H)}}{\text{Diameter of Lipo (D)}}$$

Immunofluorescence staining to test lipid rafts on 4T1 cells

4T1 cells (1×10^6) were cultured on aseptic coverslip overnight. Then, different Lipo groups were added to the cells with 1mM total lipids. 72h later, the samples were washed, and then, CT-B-488, #C22841, 1/1000, from Thermo Fisher Scientific, was added to 4T1

cells for 10 minutes at 4 °C, and then washed again. Cells were fixed in chilled PBS containing 4% formaldehyde for 15 minutes at 4 °C, and then washed 3 times with PBS. Afterwards, Hoechst 33342, #2306347, 1/2000, from Life Technologies, was added to cells and incubated for 10 min, and then cells were washed3 times with PBS. The fluorescence signals were visualized under a Zeiss LSM880 inverted confocal microscope (Zen Black software (v. 14.022.021)) at the Innovation and Impact's Imaging Core-Optical Core Facility at the University of Arizona, and the fluorescence intensity of CT-B-488 was quantified by ImageJ software (Version.1.53q)

Immunofluorescence staining to test SM levels on 4T1 cells

4T1 cells (1×10^6) were cultured on aseptic coverslip overnight. Then, different Lipo groups were added to cells with 1mM total lipids.72 h later, the samples were washed, and then the cells were incubated with lysenin, #4802-v, 250 μg/mL stock, 1/1000, from Peptide International, in PBS for 1 h and then washed. After that, rabbit anti-lysenin antibody, #MBS406486, 1/1000 from MyBioSourc and Alexa Fluor® 488-anti-rabbit antibody, #ab150077, 1/1000, from Abcam were added to the cells. Afterwards, cells were fixed in chilled PBS containing 4% formaldehyde for 15 minutes at 4 °C, and then washed 3 times with PBS. Later, Hoechst 33342, #2306347, 1/2000, from Life Technologies, was added to cells and incubated for 10 min, and then cells were washed 3 times with PBS. The fluorescence signals were visualized under a Zeiss LSM880 inverted confocal microscope (Zen Black software (v. 14.022.021)) at the Innovation and Impact's Imaging Core-Optical Core Facility at the University of Arizona, and the fluorescence signal was quantified by ImageJ software (Version.1.53q)

Western Blot for SREBP-1, NPC1 and NPC2 in 4T1 cells

4T1 cells (1 × 10^6) were cultured overnight. Then, different Lipo groups were added to cells with 1mM total lipids. 72 h later, the samples were washed with chilled PBS and lysed with RIPA cell Lysis buffer (#06182116, GenDEPOT). The supernatants were collected by centrifugation and added to loading buffer (#AR1112, Boster) and boiled for 5 min. Then, aliquots of proteins were loaded onto the gels. After electrophoresis and transferring membrane, the membranes were blocked with 0.5% milk TBST buffer for 1h, and then added by anti-SREBP-1, #ab28481, 1/1500, from Abcamor anti-NPC1, #ab134113, 1/2000, from Abcam, or anti-NPC2, #ab218192, 1/2000, from Abcam, or β-actin, #ab213262, 1/2000, from Abcam, for overnight incubation at 4 °C. The membranes were then washed with TBST and added by anti-Rabbit IgG HRP Conjugate, #W401B, 1/2000, from Promega, or 1h at room temperature and washed with TBST. Finally, the images were taken by azure biosystem 600 (v.1.9.0.0406).

Immunofluorescence staining of β-tubulin in tumours

Briefly, the dissected tumour blocks were fixed in 4% paraformaldehyde overnight, processed and then embedded by paraffin. Tumour blocks were cut into sections of 4 μm thickness. The β-tubulin in the tumour were stained with β-Tubulin Rabbit mAb (#3623S, 9F3, Alexa Fluor® 488 Conjugate, 1/100, Cell Signaling). DAPI (4,6-diamidino-2-phenylindole) was used to localize the cellular nuclei. The fluorescence signals were visualized under a Zeiss LSM880 inverted confocal microscope (Zen Black software (v. 14.022.021)) at the Innovation and Impact's Imaging Core-Optical Core Facility at the University of Arizona.

Immunohistochemistry (IHC)

The procedure of IHC staining was performed according to our previous method[1]. Briefly, the dissected tumour blocks were fixed in 4% paraformaldehyde overnight, processed and then embedded by paraffin. Tumour blocks were cut into sections of 4 μm thickness, the slides were loaded onto the Leica Bond RXm Autostainer with covertiles to prevent dehydration between staining steps. Slides were deparaffinized by heating to 60 °C, then retrieved epitope by incubating in 1 mM sodium citrate (pH = 6) at 98 °C. After cooling down to room temperature, the slides were initially rinsed in TBS wash buffer, subsequently treated with 3% H_2O_2 for 5 min to block endogenous peroxidase activity, and then incubated with individual primary antibodies for 15 to 50 minutes (anti cleaved caspase-3, #9664S, 1/300, from Cell Signaling; anti-gamma H2A.X, ab22551, 1/400 and anti-Ki67 antibody, ab15580, 1/400, from Abcam). For the TUNEL staining, a TUNEL assay kit-HRP-DAB (ab206386, Abcam) was used according to the manufacturer's protocol. Afterwards, the slides were rinsed with wash buffer, followed by incubation with HRP-conjugated anti-rabbit polymer; The slides were incubated with DAB (3,3′-Diaminobenzidine) for 10 minutes for visualization after being rinsed with wash buffer. Whereafter, the slides were washed in distilled water, counterstained with hematoxylin at room temperature for 5 minutes. The reagents are part of the Bond Polymer Refine Detection Kit (DS9800, Leica). Slides were unloaded from the Autostainer, dehydrated in increasing concentrations of ethanol, three changes of xylene, mounted with media and a cover slipped. After drying, the slide sections were observed under whole slide scanning system by Aperio ImageScope software (version 12.4.3.5008).

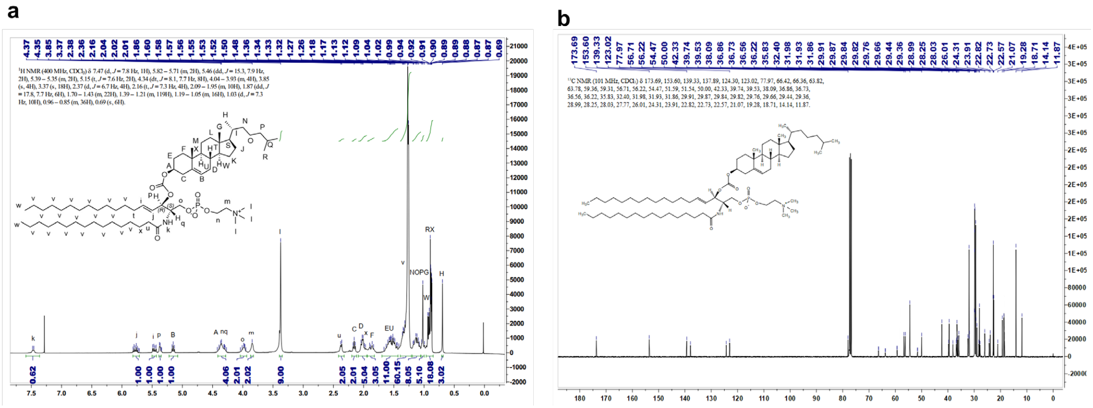

Supplementary Figure 12. ^{1}H NMR (**a**) and ^{13}C NMR (**b**) spectra for SM-C-Ester-Chol.

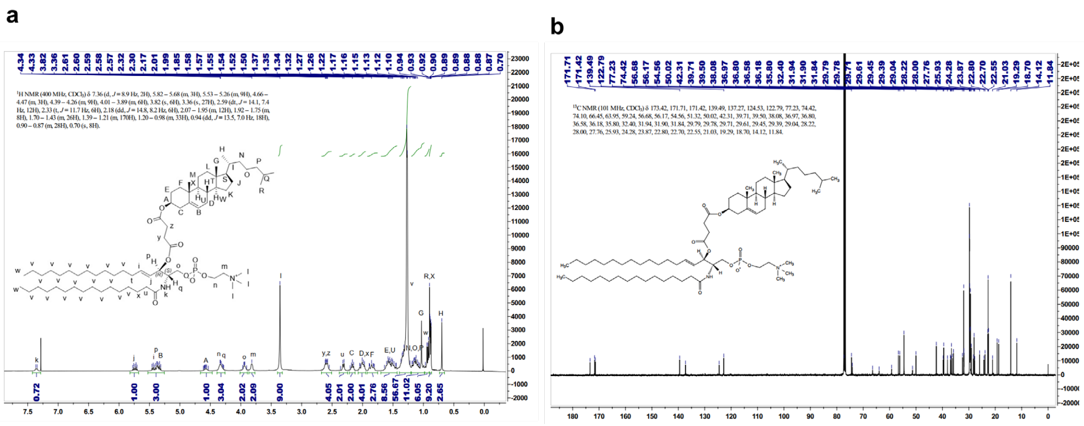

Supplementary Figure 13. ^{1}H NMR (**a**) and ^{13}C NMR (**b**) spectra for SM-Ester-Chol.

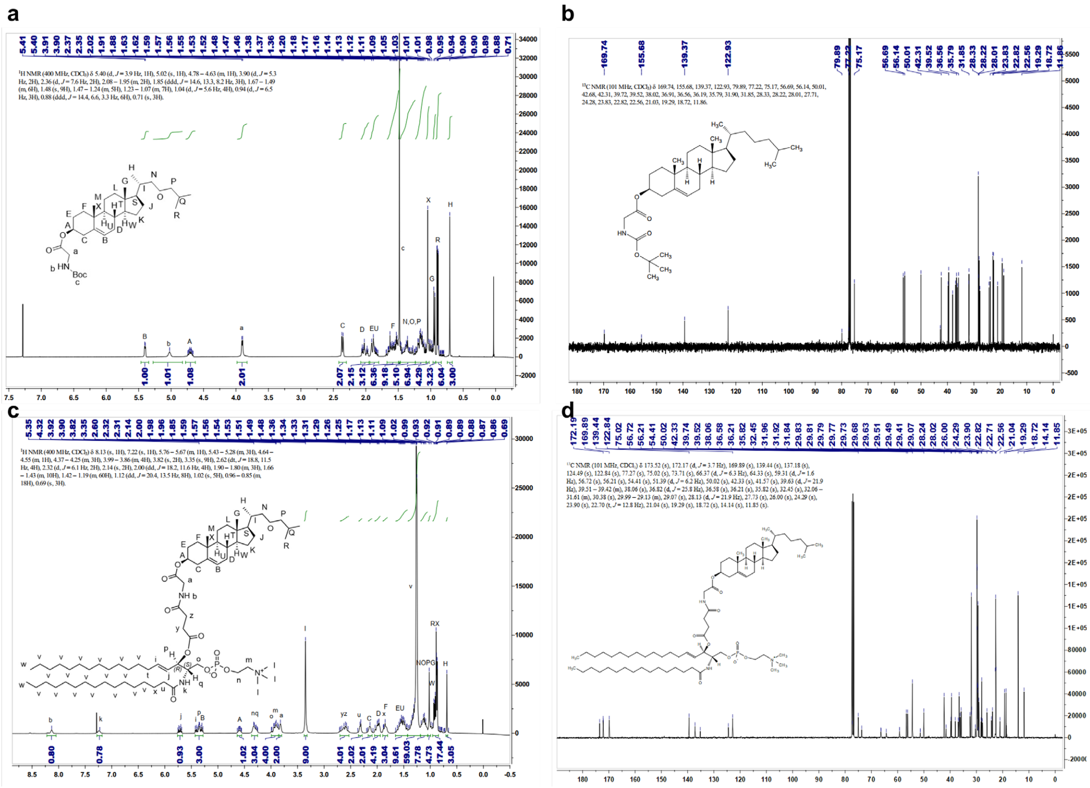

Supplementary Figure 14. ^{1}H NMR and ^{13}C NMR spectra for Chol-Glycine-Boc (**a, b**) and SM-Glycine-Chol (**c, d**).

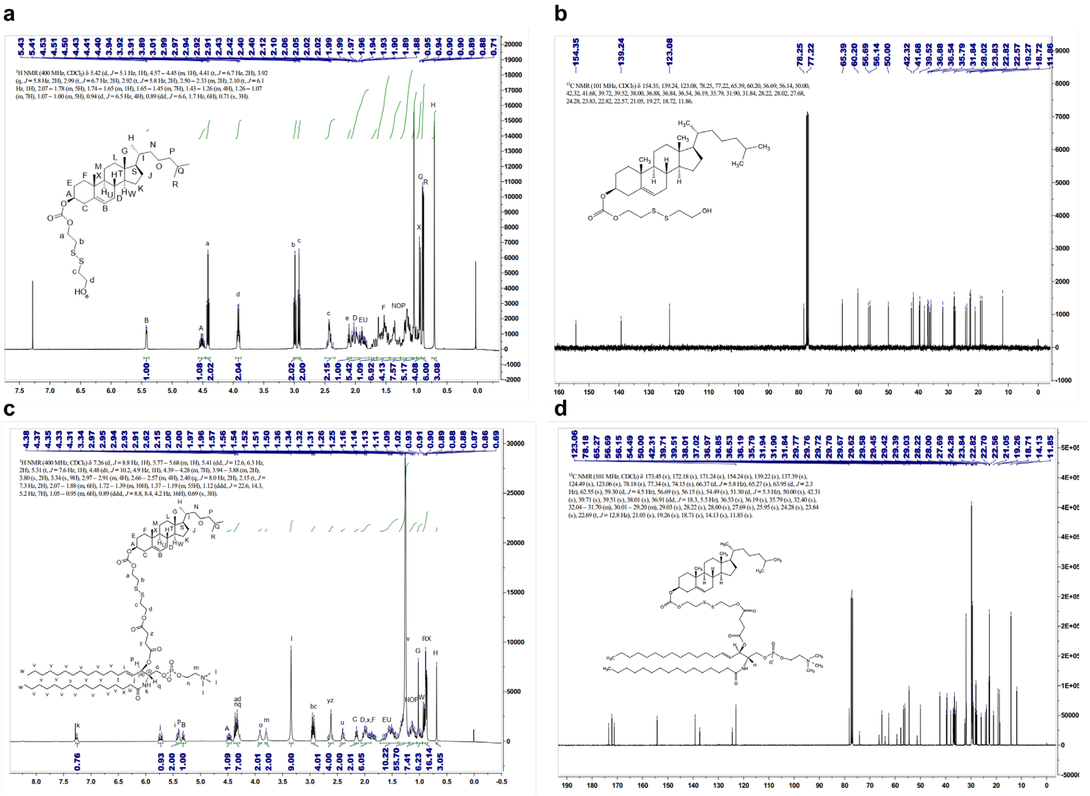

Supplementary Figure 15. ^{1}H NMR and ^{13}C NMR spectra for Chol-CSS-OH (**a, b**) and SM-CSS-Chol (**c, d**).

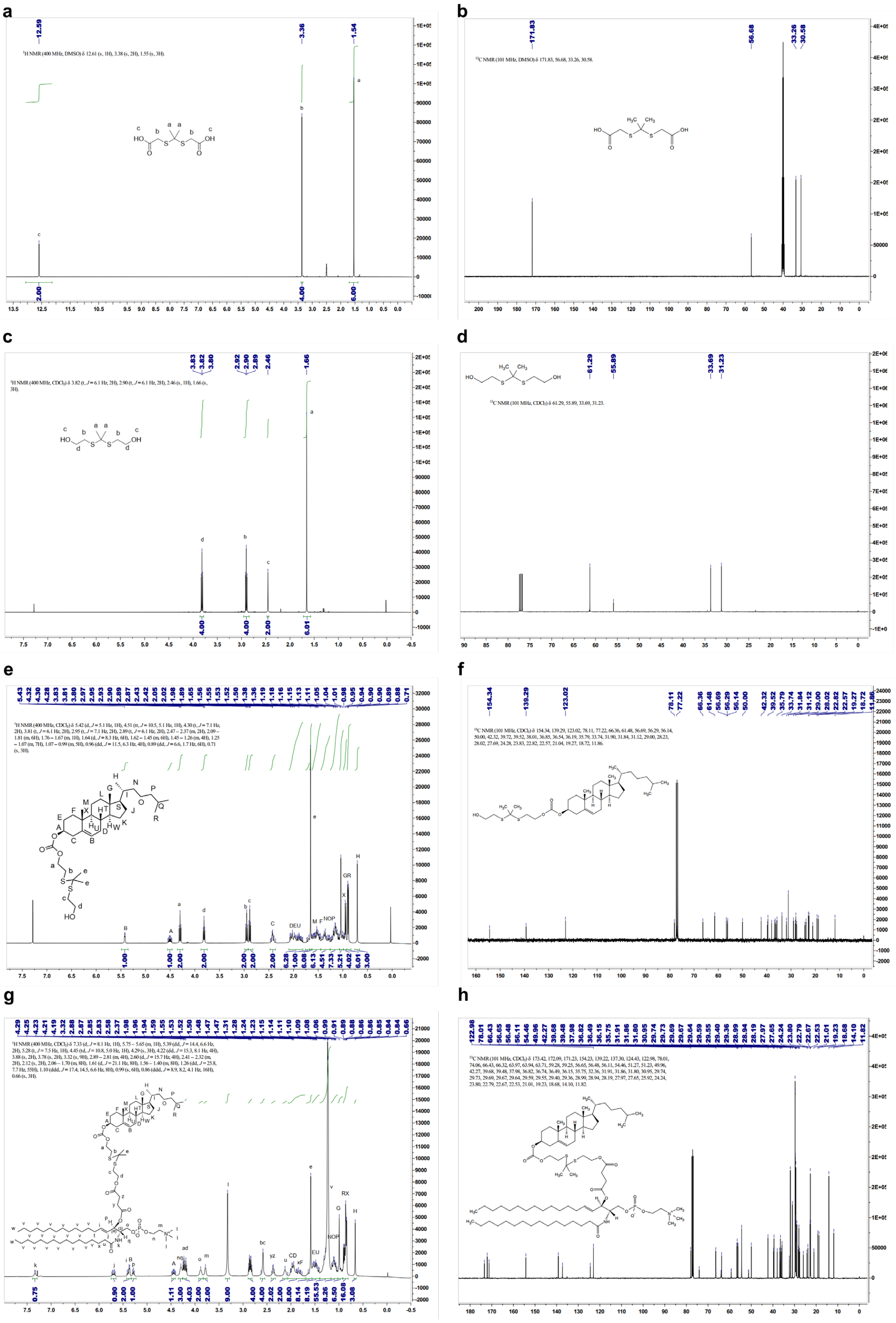

Supplementary Figure 16. ^{1}H NMR and ^{13}C NMR spectra for HOOC-SCS-COOH (**a, b**), HO-SCS-OH (**c, d**), Chol-SCS-OH (**e, f**) and SM-SCS-Chol (**g, h**).

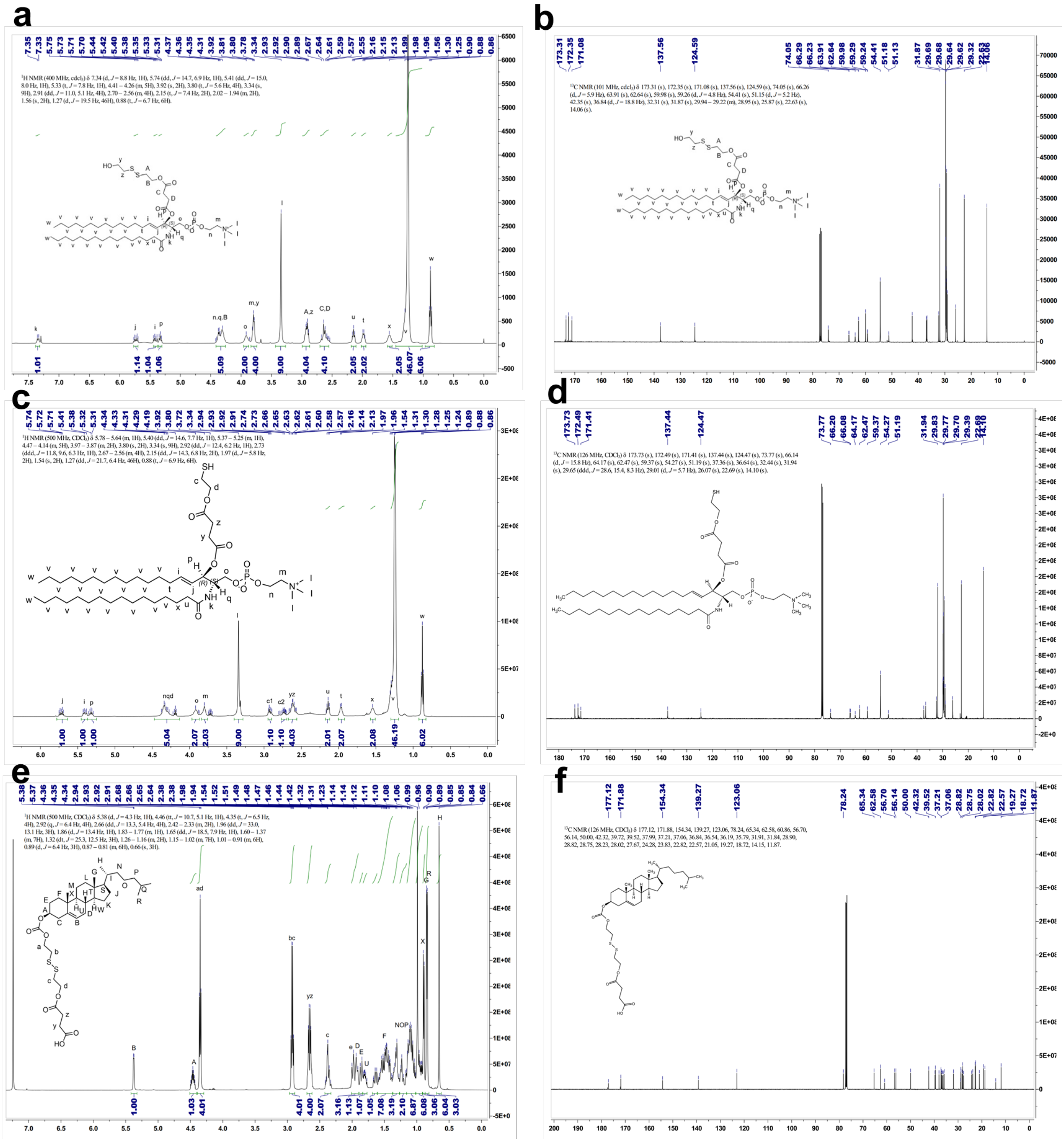

Supplementary Figure 17. ^{1}H NMR and ^{13}C NMR spectra for SM-CSS-OH (**a, b**), SM-SH (**c, d**) and Chol-CSS-COOH (**e, f**).

a

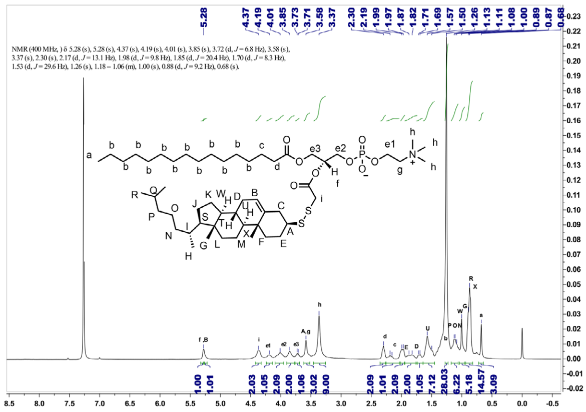

b

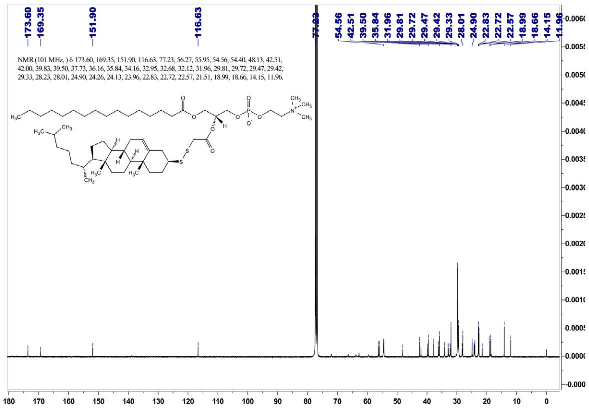

Supplementary Figure 18. ^{1}H NMR and ^{13}C NMR spectra for SML-SS (**a, b**).

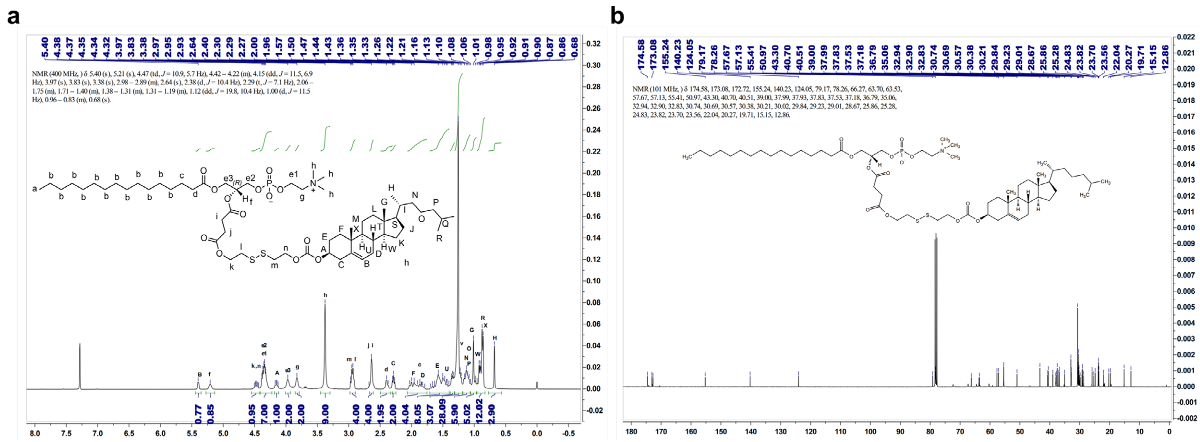

Supplementary Figure 19. ^{1}H NMR and ^{13}C NMR spectra for SML-CSS (**a, b**).

a

Liposome	DPPC (molar %)	Chol or SM-Chol or SMLs (molar %)	DLS Size by intensity (d.nm)	Zeta Potential (mV)	PDI
**Lipo-PChcPC**	57.58	42.42 (eq. 35% Chol)	112.6 ± 4.94	-7.59 ± 2.68	0.204 ± 0.056
**Lipo-PChemsPC**	57.58	42.42 (eq. 35% Chol)	117.9 ± 1.40	-4.26 ± 1.14	0.127 ± 0.003
**Lipo-OChemsPC**	57.58	42.42 (eq. 35% Chol)	105.5 ± 3.65	-3.95 ± 1.52	0.160 ± 0.031
**Lipo-DChemsPC**	78.79	21.21 (eq. 35% Chol)	128.2 ± 2.89	-2.40 ± 1.85	0.157 ± 0.041

b

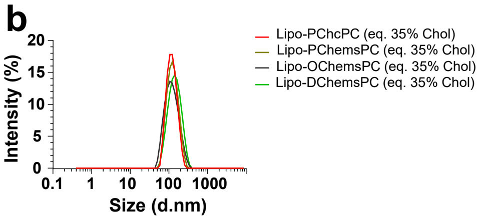

c

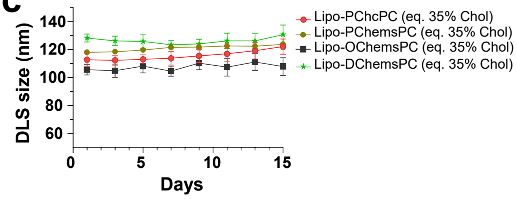

Supplementary Figure 20. **a**, A table depicting the physicochemical characterizations of various Lipo composed of SMLs (PChcPC, PChemsPC, OChemsPC, DCemsPC). d.nm: diameter values in nanometres. **b**, DLS size distribution by intensity. DLS: dynamic light scattering. **c**, The monitoring of the DLS size over time in 5% dextrose at 4 °C. Data in **a** (right portion), **c** are expressed as mean ± s.d. (n = 3 independent experiments). Source data are provided as a Source Data file.

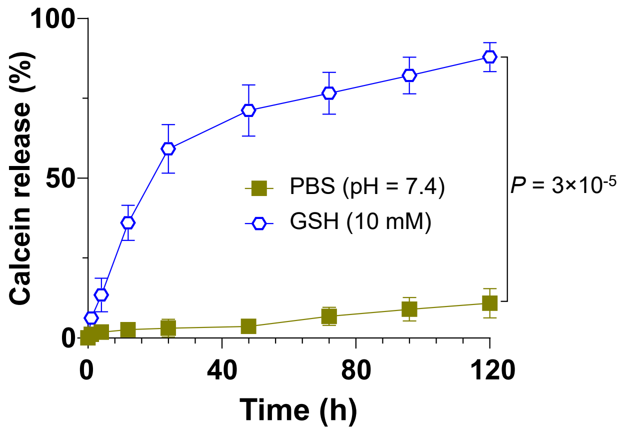

Supplementary Figure 21. The release kinetics of calcein from calcein/Lipo-SM-CSS-Chol under different conditions at 37 °C. Data are expressed as mean ± s.d. (n=3 independent experiments). Statistical significance was determined by two-tailed, unpaired Student's t-test. Source data are provided as a Source Data file.

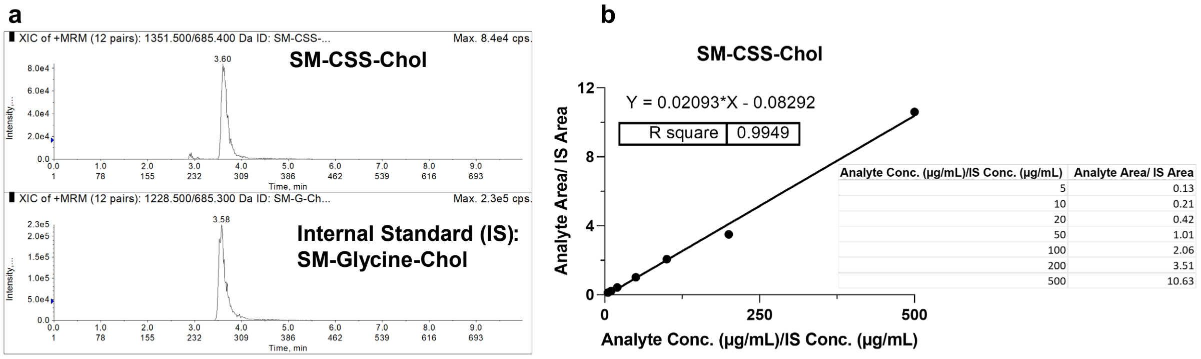

Analyte Conc. (µg/mL)/IS Conc. (µg/mL)	Analyte Area/ IS Area
5	0.13
10	0.21
20	0.42
50	1.01
100	2.06
200	3.51
500	10.63

Supplementary Figure 22. The LC-MS/MS method development for SM-CSS-Chol concentration measurement in stability studies. Representative LC-MS/MS chromatogram (**a**), and standard curve (**b**) for SM-CSS-Chol. SM-Glycine-Chol was used as the internal standard (IS). Source data are provided as a Source Data file.

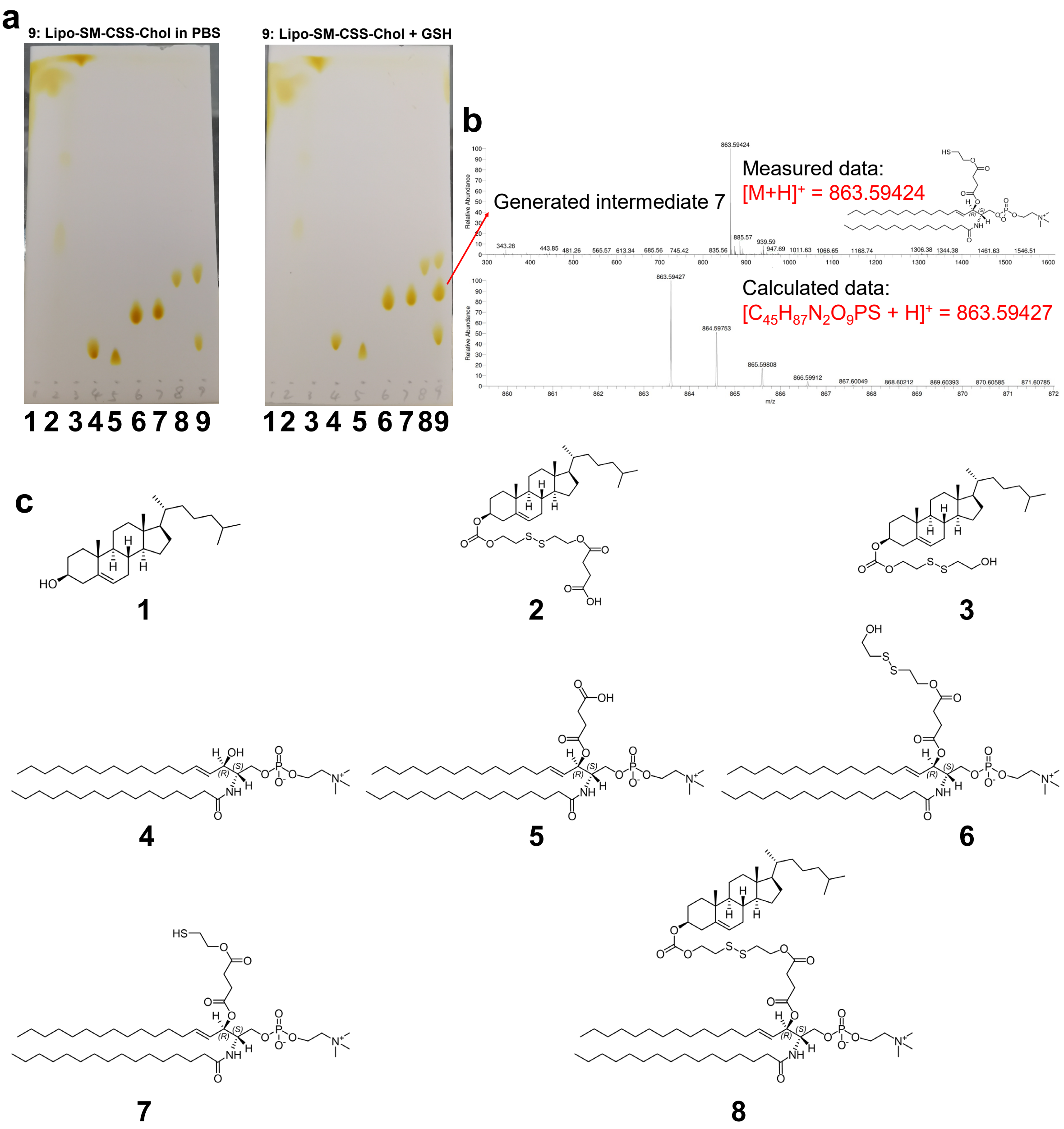

Supplementary Figure 23. TLC (**a**) and high-resolution LC-MS (HRMS) (**b**) to study the possible intermediates generated (**c**) after incubating Lipo-SM-CSS-Chol in PBS and GSH (10 mM) for 24 h under 37 °C.

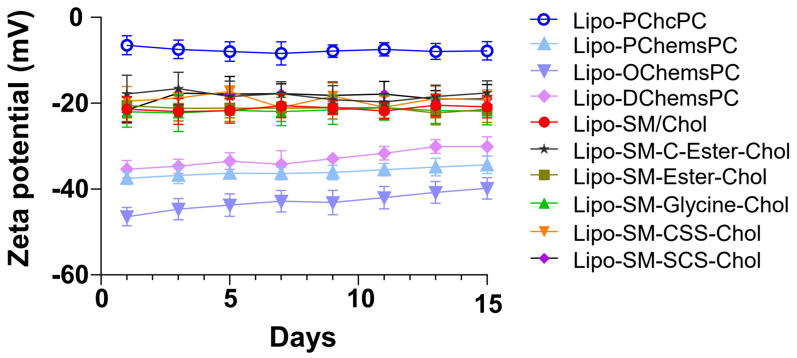

Supplementary Figure 24. Zeta potential monitoring for various Lipo over a 15-day period in 5% dextrose at 4 °C. Data are represented as mean ± s.d. (n = 3 independent experiments). Source data are provided as a Source Data file.

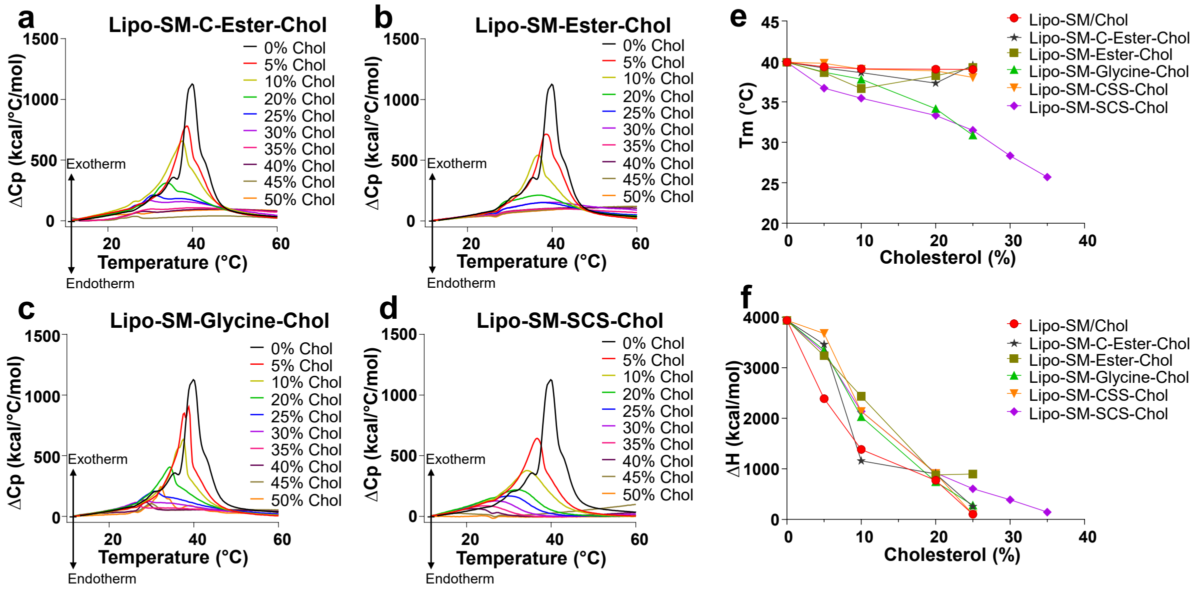

Supplementary Figure 25. Thermotropic phase transition behavior examined by differential scanning calorimetry (DSC). DSC thermograms of Lipo-SM-C-Ester-Chol (**a**), Lipo-SM-Ester-Chol (**b**), Lipo-SM-Glycine-Chol (**c**) and Lipo-SM-SCS-Chol (**d**) at various eq. mol % Chol. (**e**) The effects of SM-Chol on transition temperature (**e**) and enthalpy (**f**). Source data are provided as a Source Data file.

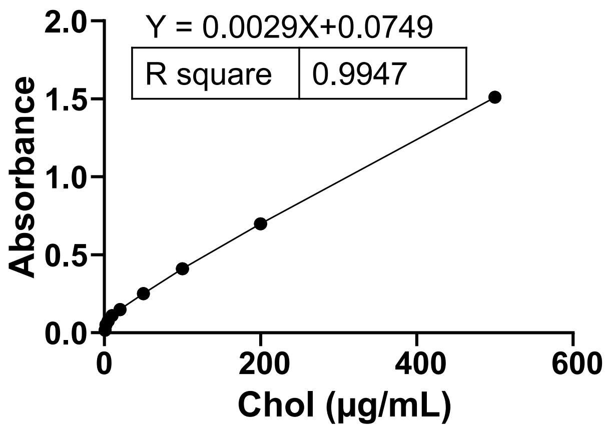

Supplementary Figure 26. The standard curve for Chol was determined by the Chol assay described in Chol exchange method section. Source data are provided as a Source Data file.

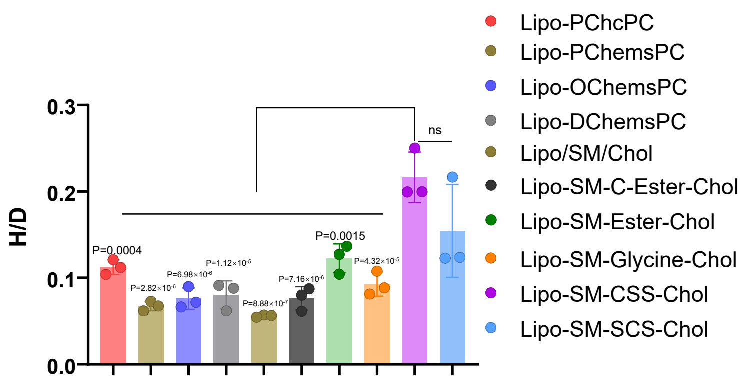

Supplementary Figure 27. The H/D (height/diameter) ratio for various Lipo measured by atomic force microscopy (AFM). Data are represented as mean ± s.d. (n = 3 independent experiments). Statistical significance was determined by one-way ANOVA followed by Tukey's multiple comparisons test. Source data are provided as a Source Data file.

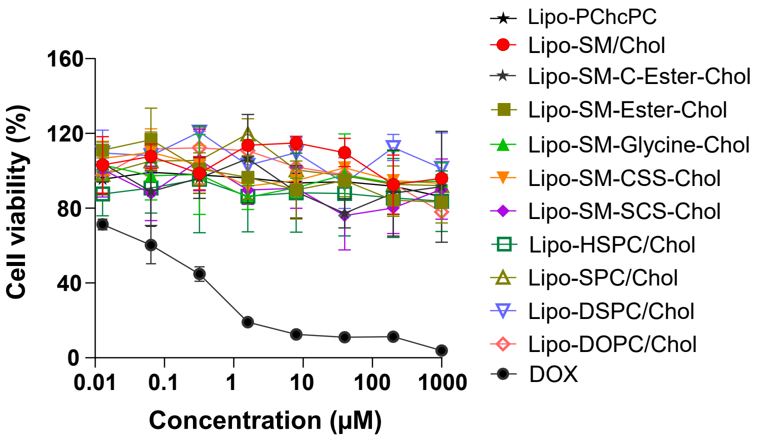

Supplementary Figure 28. The cytotoxicity of different Lipo-SM-Chol and Lipo composed of various conventional phospholipid/Chol mixtures in 4T1-Luc2 cells. Cells were treated for 72 h and cell viability was determined by MTT assay. DOX was used as the positive control. Data are represented as mean ± s.d. (n = 3 samples). Source data are provided as a Source Data file.

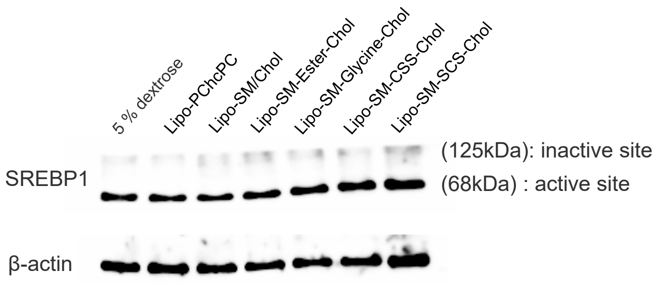

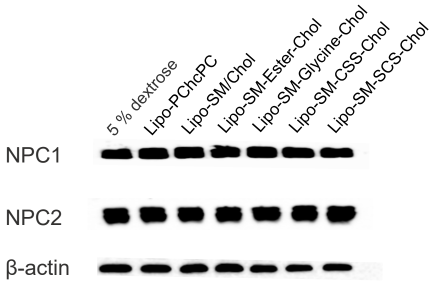

Supplementary Figure 29. The western blot to study the effects of various Lipo on sterol regulatory element-binding protein 1 (SREBP-1), and Niemann Pick C1 (NPC1) and Niemann Pick C2 (NPC2) levels in 4T1 cells. (n = 3 experiments, similar results were observed). Source data are provided as a Source Data file.

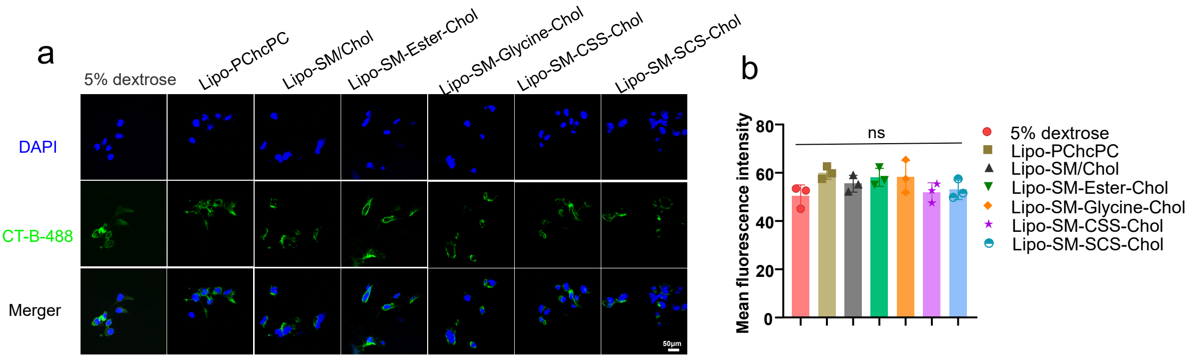

Supplementary Figure 30. Confocal laser scanning microscopy (CLSM) to study the effects of various Lipo on lipid rafts levels on 4T1 cells. (Scale bar 50 μm) (**a**, n = 3 independent experiments, similar results were observed). The fluorescence signal of CT-B-488 was quantified by ImageJ software (Version.1.53q), (**b**). Data in **b** are represented as mean ± s.d. (n = 3 independent experiments). Statistical significance was determined by one-way ANOVA followed by Tukey's multiple comparisons test. Source data are provided as a Source Data file.

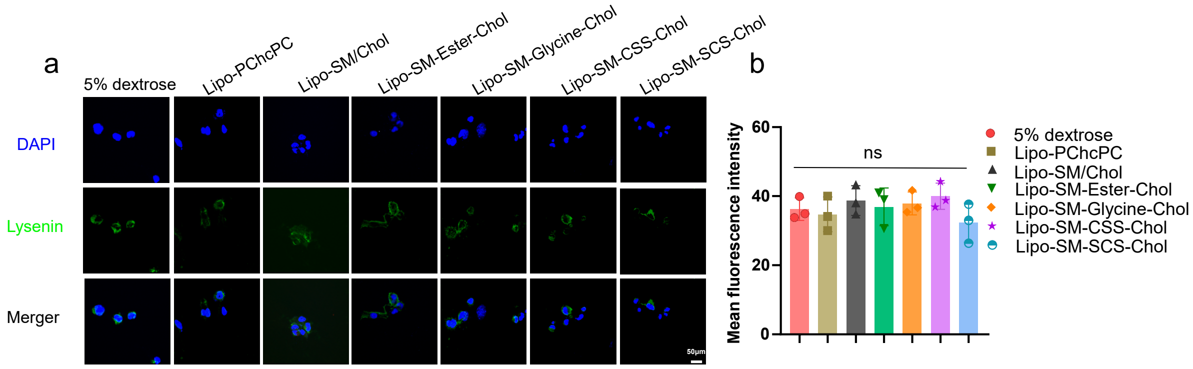

Supplementary Figure 31. CLSM to study the effects of various Lipo on SM levels on 4T1 cells. (Scale bar 50 μm) (**a**, n = 3 independent experiments, similar results were observed). The fluorescence signal was quantified by ImageJ software (Version.1.53q) (**b**). Data in **b** are represented as mean ± s.d. (n = 3 independent experiments). Statistical significance was determined by one-way ANOVA followed by Tukey's multiple comparisons test. Source data are provided as a Source Data file.

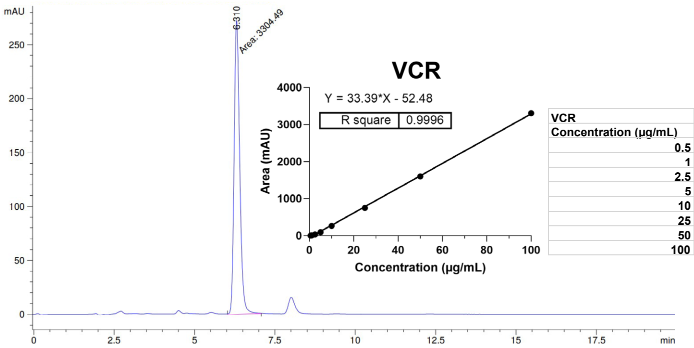

VCR Concentration (µg/mL)	Area
**0.5**	1.547
**1**	5.0467
**2.5**	33.29
**5**	96.221
**10**	266.35
**25**	749.4
**50**	1601.7
**100**	3304.5

**Instrumentation**	
**HPLC Unit**	Agilent 1100 (USA)
**Detector**	Agilent G1315B Diode-Array Detector
**Integrator**	Agilent ChemStation Software
**Chromatographic conditions**	
**Column**	Waters Symmetry ODS (250mm X 4 mm X 5 µm)
**Mobile Phase**	ACN : NH_4Ac (15 mM) (55:45 v/v)
**Flow Rate**	1 mL/min
**Injection Volume**	50 µL
**λ_{max}**	254 nm

Supplementary Figure 32. The Analytic Reverse-phase High Performance Liquid Chromatography (HPLC) method development for VCR concentration measurement in pharmacokinetics and biodistribution studies. Representative HPLC chromatogram, standard curve, and HPLC instrumentation and chromatographic conditions for VCR. Source data are provided as a Source Data file.

a

VCR-laden Lipo	VCR DLC (%)				VCR DLE (%)			
	#1	#2	#3	Mean ± SD	#1	#2	#3	Mean ± SD
VCR/Lipo-PChcPC	8.86	8.53	8.98	8.79 ± 0.23	88.61	85.32	89.8	87.91 ± 2.32
VCR/Lipo-SM/Chol	9.12	9.03	8.91	9.02 ± 0.11	95.2	86.27	89.01	90.16 ± 4.57
VCR/Lipo-SM-C-Ester-Chol	0.51	1.24	0.68	0.81 ± 0.38	5.61	12.89	7.42	8.64 ± 3.79
VCR/Lipo-SM-Ester-Chol	8.02	9.21	9.29	8.84 ± 0.71	80.3	92.24	92.9	88.48 ± 7.09
VCR/Lipo-SM-Glycine-Chol	1.02	0.79	2.69	1.50 ± 1.04	11.33	8.24	27.8	15.79 ± 10.52
VCR/Lipo-SM-CSS-Chol	9.03	9.01	8.99	9.01 ± 0.02	92.65	91.38	86.33	90.12 ± 3.34
VCR/Lipo-SM-SCS-Chol	7.89	8.84	7.93	8.22 ± 0.54	78.96	88.46	79.45	82.29 ± 5.35

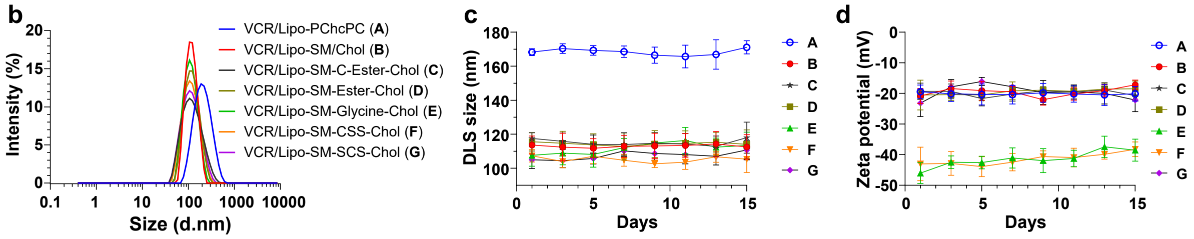

Supplementary Figure 33. (**a**) The DLC and DLE of various VCR/Lipo from 3 independent samples. The representative DLS size distribution by intensity (**b**) for VCR/Lipo-PChcPC, VCR/Lipo-SM/Chol and various VCR/Lipo-SM-Chol, and the DLS Size (**c**) and zeta potential (**d**) monitoring over a 15-day period at 4 °C. Data in **c, d** are represented as mean ± s.d. (n = 3 independent experiments). Source data are provided as a Source Data file.

	Body weight lose vs day 1																					P values		
Day	3 mg/kg						Mean	4 mg/kg						Mean	5 mg/kg						Mean	3 mg/kg vs 4 mg/kg	3 mg/kg vs 5 mg/kg	4 mg/kg vs 5 mg/kg
	#1	#2	#3	#4	#5	#6		#1	#2	#3	#4	#5	#6		#1	#2	#3	#4	#5	#6				
2	-9.00%	-8.59%	-5.70%	-8.59%	-1.90%	-0.52%	-5.72%	-7.27%	-8.62%	-10.85%	-8.18%	-13.85%	-3.79%	-8.76%	-9.13%	-6.22%	-6.86%	-16.51%	-11.90%	-10.19%	-10.14%	0.5149	0.2501	0.8724
4	-15.17%	-13.64%	-5.70%	-3.54%	0.95%	-1.04%	-6.35%	-4.09%	-5.60%	-12.74%	-9.55%	-14.72%	-13.27%	-9.99%	-11.87%	-10.53%	-9.31%	-21.56%	-20.95%	-14.08%	-14.72%	0.3333	0.0086	0.2478
6	-7.11%	-12.63%	-4.66%	-2.02%	0.95%	-1.04%	-4.42%	-7.73%	-12.50%	-12.74%	-13.64%	-13.42%	-12.80%	-12.14%	-20.09%	-11.00%	-18.14%	-30.28%	-27.14%	-25.24%	-21.98%	0.0477	0.00007	0.011
8	-3.79%	-4.55%	-3.11%	0.00%	6.19%	-0.52%	-0.96%	-8.64%	-3.45%	-10.38%	-14.09%	-8.66%	-13.74%	-9.83%	**Died**			**Died**	**Died**			0.0034		
10	2.84%	-5.05%	6.22%	0.00%	0.95%	-0.52%	0.74%	-6.36%	-4.31%	-5.19%	-13.18%	-8.66%	-13.27%	-8.50%								0.0021		
12	2.37%	-4.04%	2.59%	-1.01%	0.00%	-1.04%	-0.19%	-6.82%	-6.03%	-4.25%	-12.27%	-9.09%	-12.32%	-8.46%								0.0029		
14	4.27%	-6.06%	3.11%	0.00%	1.90%	-0.52%	0.45%	-1.36%	-12.93%	-4.72%	-11.82%	-8.23%	-11.85%	-8.48%								0.0012		

Supplementary Figure 34. The detailed body weight loses vs the day 1 after iv injection of VCR/Lipo-SM/Chol and the statistical analysis (n = 6 mice). Statistical significance was determined by one-way ANOVA followed by Tukey's multiple comparisons test on day 2, 4, and 6, and two-tailed, unpaired Student's t-test (for 3 and 4 mg/kg as there were 3 mice deaths in 5 mg/kg group on day 8) on day 8, 10, 12, and 14. Source data are provided as a Source Data file.

	P value by two-tailed, unpaired Student's t-test (vs VCR/Lipo-SM-CSS-Chol)			
	VCR/Lipo-PChcPC	VCR/Lipo-SM/Chol	VCR/Lipo-SM-Ester-Chol	VCR/Lipo-SM-SCS-Chol
$T_{1/2}$ (h)	10^{-8}	0.0171	2×10^{-7}	10^{-8}
V ((μg)/(μg/ml))	0.9999	0.9999	0.9999	0.9999
CL(μg)/(μg/ml)/h)	0.8587	0.9999	0.9410	0.9710
$AUC_{0\text{-}t}$ (μg/ml*h)	6×10^{-9}	0.0025	5×10^{-9}	7×10^{-9}
$AUC_{0\text{-}inf}$ (μg/ml*h)	6×10^{-9}	0.0024	6×10^{-9}	8×10^{-9}
AUMC (μg/ml*h^2)	4×10^{-7}	0.0026	4×10^{-7}	4×10^{-7}
MRT (h)	10^{-8}	0.0165	10^{-8}	10^{-8}
V_{ss} (μg/(μg/ml))	0.9999	0.9999	0.9999	0.9999

Supplementary Figure 35. Statistical significance comparison of the PK parameters (*VS* VCR/Lipo-SM-CSS-Chol, n = 3 mice) in **Fig. 3c**. Statistical significance was determined by two-tailed, unpaired Student's t-test. Source data are provided as a Source Data file.

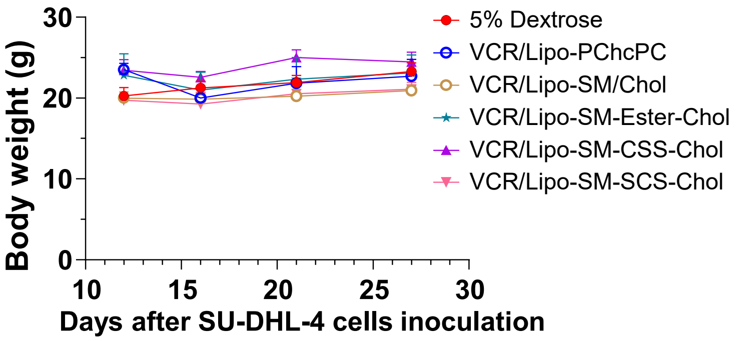

Supplementary Figure 36. Mice body weight monitoring in therapeutic efficacy study presented in **Fig. 3d-f.** Data are represented as mean ± s.d. (n = 5 mice). Source data are provided as a Source Data file.

a

MU-P/DiD laden Lipo	DiD (molar %)	MU-P DLC (%)	MU-P DLE (%)	DLS Size by intensity (d.nm)	Zeta Potential (mV)	PDI
**MU-P/DiD/Lipo-PChcPC** (eq. 35% Chol)	0.10	12.20 ± 0.41	18.57 ± 1.20	121.4 ± 8.6	-9.4 ± 4.8	0.198 ± 0.025
**MU-P/DiD/Lipo-PChcPC** (eq. 64.4% Chol)	0.10	12.17 ± 0.26	18.36 ± 0.91	119.1 ± 6.4	-7.2 ± 3.2	0.183 ± 0.034
**MU-P/DiD/Lipo-SM/Chol**	0.10	12.29 ± 0.37	18.80 ± 0.86	124.6 ± 5.3	-18.9 ± 3.5	0.157 ± 0.023
**MU-P/DiD/Lipo-SM-Ester-Chol**	0.10	12.33 ± 0.41	18.71 ± 0.95	116.7 ± 4.8	-8.1 ± 2.4	0.173 ± 0.028
**MU-P/DiD/Lipo-SM-CSS-Chol**	0.10	12.14 ± 0.29	18.45 ± 0.73	107.3 ± 6.5	-8.7 ± 2.8	0.159 ± 0.038
**MU-P/DiD/Lipo-SM-SCS-Chol**	0.10	12.17 ± 0.35	18.32 ± 0.69	118.3 ± 7.4	-6.5 ± 1.2	0.175 ± 0.029

b

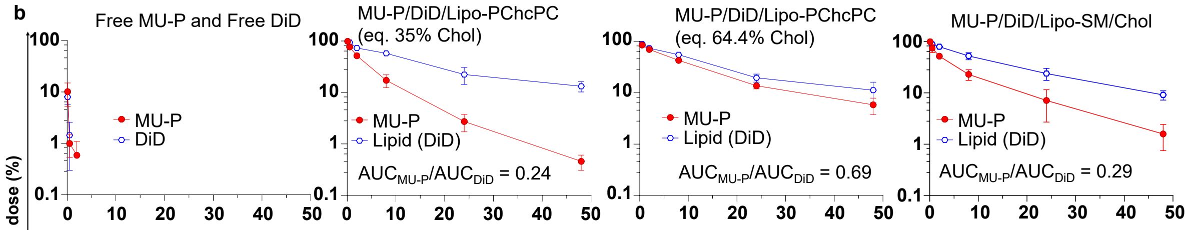

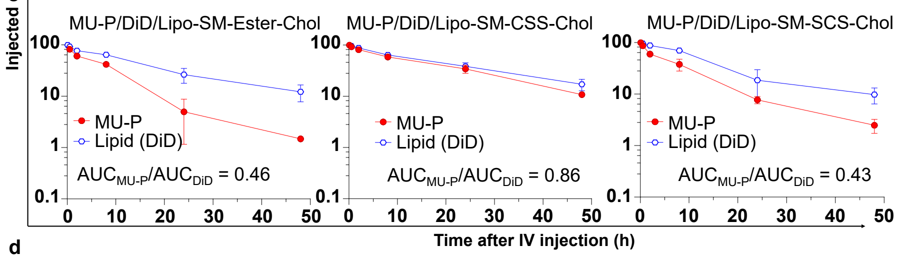

c

MU-P/DiD laden Lipo	AUC_{MU-P}/AUC_{DiD}
**MU-P/DiD/Lipo-PChcPC** (eq. 35% Chol)	0.24
**MU-P/DiD/Lipo-PChcPC** (eq. 64.4% Chol)	0.69
**MU-P/DiD/Lipo-SM/Chol**	0.29
**MU-P/DiD/Lipo-SM-Ester-Chol**	0.46
**MU-P/DiD/Lipo-SM-CSS-Chol**	0.86
**MU-P/DiD/Lipo-SM-SCS-Chol**	0.43

d

	**MU-P/DiD/Lipo-PChcPC (eq. 35% Chol)**		**MU-P/DiD/Lipo-PChcPC (eq. 64.4% Chol)**		**MU-P/DiD/ Lipo-SM/Chol**	
	**MU-P**	**Lipid (DiD)**	**MU-P**	**Lipid (DiD)**	**MU-P**	**Lipid (DiD)**
**$T_{1/2}$ (h)**	2.62±0.58	11.61±2.37	7.23±0.76	10.81±1.78	3.16±0.27	11.00±2.11
**V ((μg)/(μg/ml))**	1.06±0.04	1.06±0.02	1.07±0.01	1.07±0.02	1.10±0.07	1.06±0.03
**CL(μg)/(μg/ml)/h)**	0.29±0.05	0.06±0.01	0.10±0.01	0.07±0.01	0.24±0.02	0.07±0.01
**AUC_{0-t} (μg/ml*h)**	1,426.14±282.98	5,939.06±1,016.99	3,844.22±396.83	5,530.87±754.65	1,660.88±140.75	5,669.96±960.58
**AUC_{0-inf} (μg/ml*h)**	1,426.16±283.01	6,341.31±1,334.83	3,886.45±422.95	5,820.77±920.90	1,660.94±140.78	5,994.66±1,215.96
**AUMC (μg/ml*h^2)**	5,539.58±2,360.38	109,214.88±46,403.2	40,856.13±8,958.26	92,301.71±28,471.58	7,602.47±1,178.59	97,576.07±39,600.35
**MRT (h)**	3.77±0.84	16.74±3.43	10.43±1.10	15.59±2.57	4.56±0.39	15.87±3.04
**V_{ss} (μg/(μg/ml))**	1.06±0.04	1.06±0.02	1.07±0.01	1.07±0.02	1.10±0.07	1.06±0.03
	**MU-P/DiD/Lipo-SM-Ester-Chol**		**MU-P/DiD/Lipo-SM-CSS-Chol**		**MU-P/DiD/Lipo-SM-SCS-Chol**	
	**MU-P**	**Lipid (DiD)**	**MU-P**	**Lipid (DiD)**	**MU-P**	**Lipid (DiD)**
**$T_{1/2}$ (h)**	5.95±1.12	13.57±2.17	14.59±1.90	17.19±3.11	5.37±1.35	12.24±2.68
**V ((μg)/(μg/ml))**	1.10±0.04	1.06±0.02	1.06±0.03	1.03±0.02	1.07±0.04	1.00±0.01
**CL(μg)/(μg/ml)/h)**	0.13±0.03	0.05±0.01	0.05±0.01	0.04±0.01	0.14±0.03	0.06±0.01
**AUC_{0-t} (μg/ml*h)**	3,120.55±629.07	6,745.03±815.95	7,088.64±494.32	8,212.81±797.48	2,875.67±696.25	6,573.61±1,159.35
**AUC_{0-inf} (μg/ml*h)**	3,137.01±647.19	7,415.25±1,134.11	7,917.68±834.44	9,654.96±1,535.87	2,886.33±709.44	7,095.61±1,535.44
**AUMC (μg/ml*h^2)**	27,627.94±10,982.46	147,497.05±43,354.2	168,197.83±40,576.0	244,062.89±84,823.1	23,258.98±11,302.94	129,216.54±54,712.4
**MRT (h)**	8.59±1.62	19.57±3.12	21.05±2.75	24.80±4.49	7.74±1.95	17.65±3.87
**V_{ss} (μg/(μg/ml))**	1.10±0.04	1.06±0.02	1.06±0.03	1.03±0.02	1.07±0.04	1.00±0.01

e

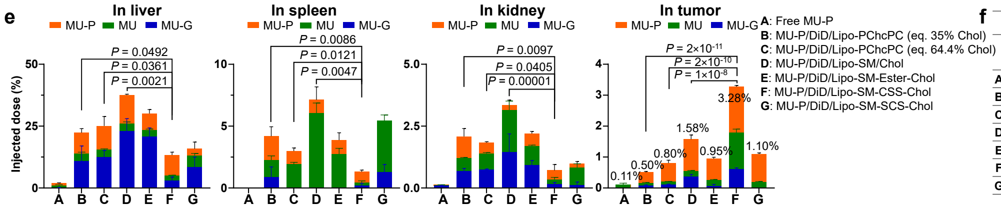

f

	**In tumors**	
	**MU + MU-G dose %**	**Release ratio %**
**A**	0.11 ± 0.08	100.00 ± 0.00
**B**	0.16 ± 0.09	31.16 ± 12.49
**C**	0.20 ± 0.05	24.58 ± 2.35
**D**	0.54 ± 0.10	34.85 ± 9.75
**E**	0.25 ± 0.11	26.19 ± 8.99
**F**	1.79 ± 0.13	54.49 ± 4.85
**G**	0.19 ± 0.03	17.16 ± 2.13

Supplementary Figure 37. Improved the stability in circulation, tumor delivery and drug release of MU-P/DiD/Lipo-SM-CSS-Chol. a, A table showing the physicochemical characterizations of various MU-P/DiD/Lipo (n = 3 independent samples). **b-f,** Blood kinetics (**b**), the AUC_{MU-P}/AUC_{DiD} ratio (**c**), pharmacokinetic parameters (**d**), biodistribution (**e**) and release ratio of MU-P in tumor (**f**) of free MU-P and MU-P/DiD/Lipo in orthotopic KPC-Luc pancreatic ductal adenocarcinoma (PDAC) mouse model (n=3; tumour: ~400 mg) following a single i.v. administration at 20 mg MU-P/kg, 0.25 mg DiD/kg. Data are represented as mean ± s.d. (n = 3 mice). Statistical significance in **e** was determined by two-tailed, unpaired Student's t-test. Source data are provided as a Source Data file.

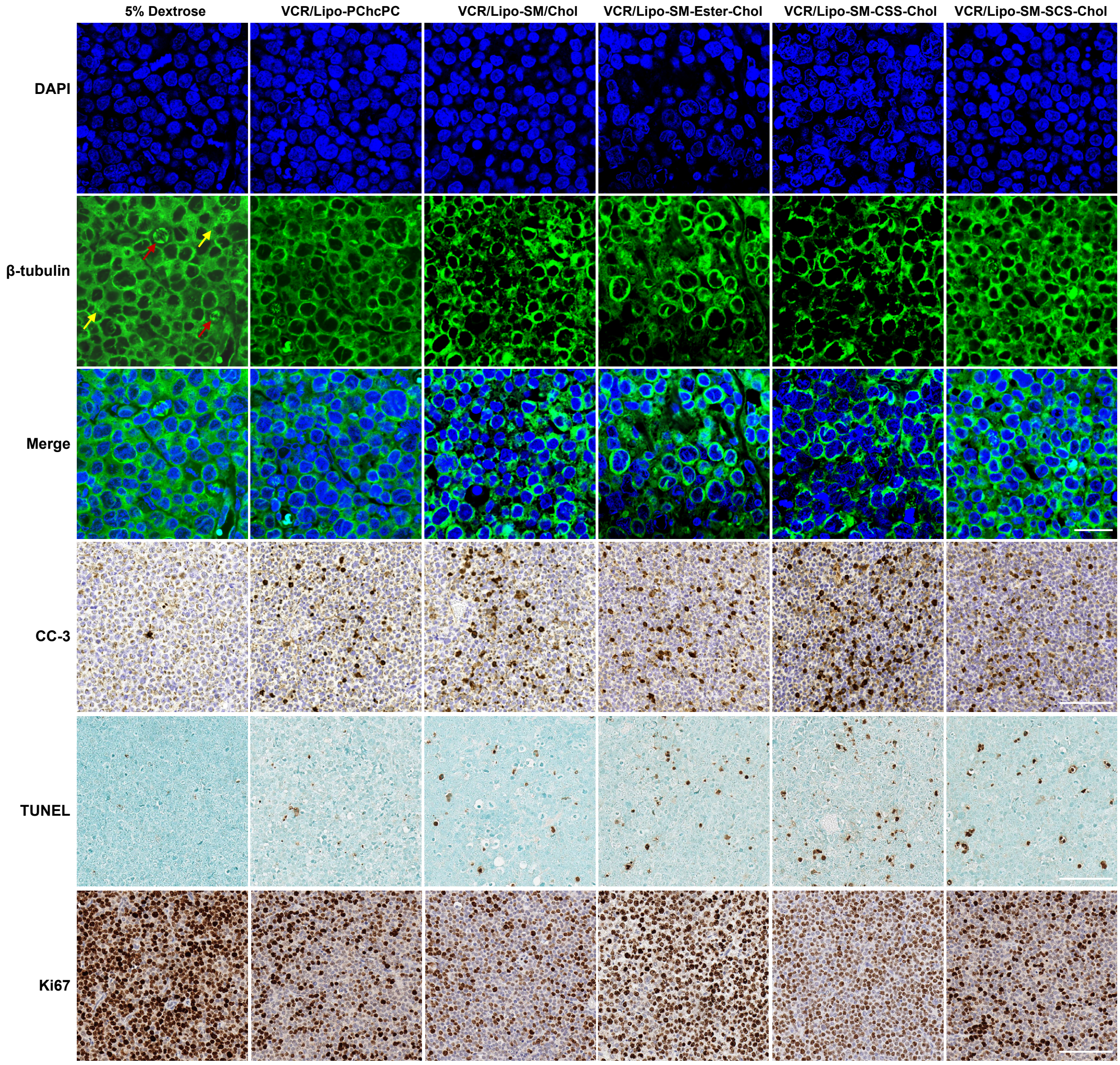

Supplementary Figure 38. Representative immunofluorescence staining for β-tubulin. (Yellow arrow: filiform microtubules; red arrow: bipolar mitotic divisions, scale bar: 50 μm) and IHC staining for CC-3, TUNEL and Ki67, scale bar: 100 μm of SU-DHL-4 diffuse large B-cell lymphoma from an independent efficacy study after receiving the same treatments as **Fig. 3e**. (n = 5 mice, similar results were observed).

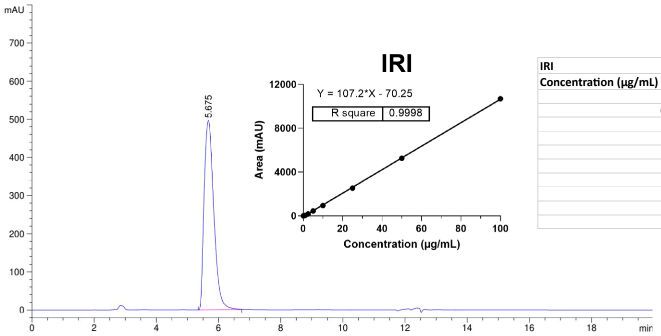

IRI	
**Concentration (µg/mL)**	**Area**
**0.1**	5.03
**0.25**	9.22
**0.5**	16.45
**1**	46.84
**2.5**	185.059
**5**	442.609
**10**	943.1439
**25**	2532.49
**50**	5263.65
**100**	10692.5

**Instrumentation**	
**HPLC Unit**	Agilent 1100 (USA)
**Detector**	Agilent G1315B Diode-Array Detector
**Integrator**	Agilent ChemStation Software
**Chromatographic conditions**	
**Column**	Waters Symmetry ODS (250mm X 4 mm X 5 µm)
**Mobile Phase**	ACN : NH_4Ac (15 mM) (70:30 v/v)
**Flow Rate**	1 mL/min
**Injection Volume**	50 µL
**λ_{max}**	370 nm

Supplementary Figure 39. The Analytic Reverse-phase High Performance Liquid Chromatography (HPLC) method development for IRI concentration measurement in pharmacokinetics and biodistribution studies. Representative HPLC chromatogram, standard curve, and HPLC instrumentation and chromatographic conditions for IRI. Source data are provided as a Source Data file.

IRI-laden Lipo	IRI DLC (%)				IRI DLE (%)			
	#1	#2	#3	Mean ± SD	#1	#2	#3	Mean ± SD
IRI/Lipo-PChcPC	5.98	5.02	5.5	5.50 ± 0.48	59.82	50.21	55	55.01 ± 4.81
IRI/Lipo-SM/Chol	6.02	5.26	5.58	5.62 ± 0.38	62.24	53.27	55.73	57.08 ± 4.63
IRI/Lipo-SM-C-Ester-Chol	5.68	4.86	4.43	4.99 ± 0.64	59.8	51.78	46.25	52.61 ± 6.81
IRI/Lipo-SM-Ester-Chol	5.04	6.17	5.02	5.41 ± 0.66	50.37	61.68	50.19	54.08 ± 6.58
IRI/Lipo-SM-Glycine-Chol	0.87	0.62	0.49	0.66 ± 0.19	8.79	6.25	5.03	6.69 ± 1.92
IRI/Lipo-SM-CSS-Chol	9.95	9.55	9.87	9.79 ± 0.21	96.81	91.16	97.12	95.03 ± 3.36
IRI/Lipo-SM-SCS-Chol	8.07	9.26	8.89	8.74 ± 0.61	80.71	92.59	88.9	87.40 ± 6.08

Supplementary Figure 40. The DLC and DLE of various IRI/Lipo (n =3 independent samples). Source data are provided as a Source Data file.

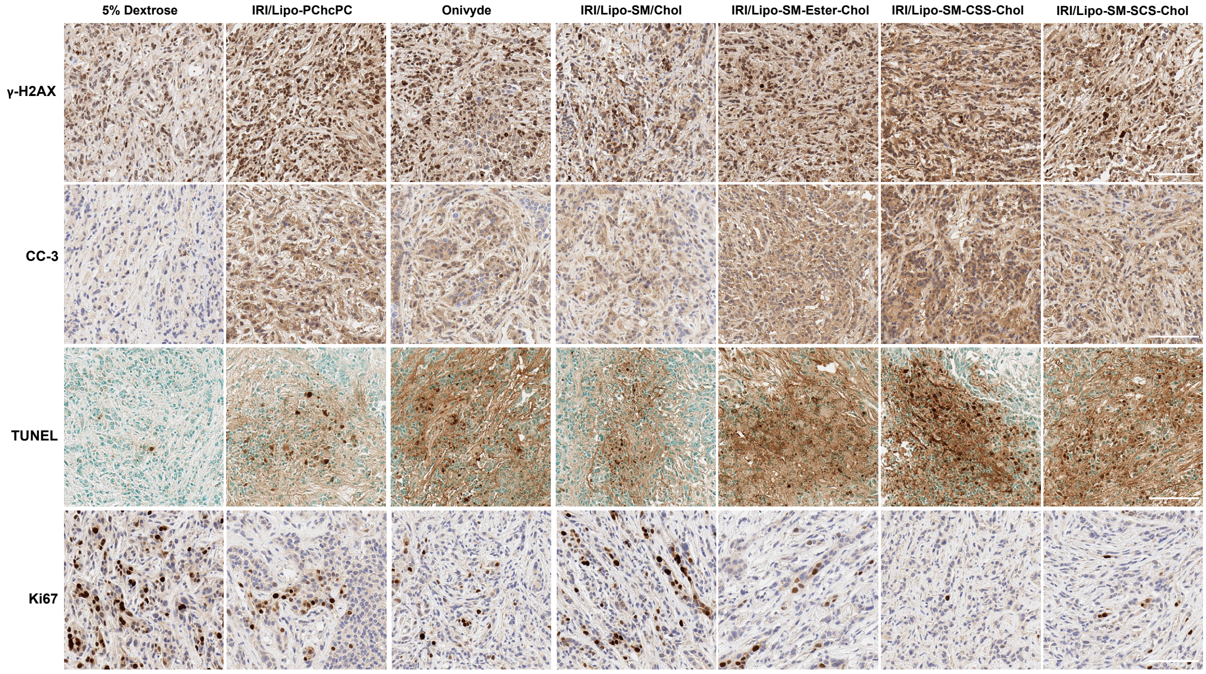

Supplementary Figure 41. Representative IHC staining for γ-H2AX, CC-3, TUNEL and Ki67 in orthotopic KPC-Luc tumors from **Fig. 4c-e**. (n = 6 mice, scale bar: 100 μm, similar results were observed).

a

IRI-laden Lipo	SM (molar %)	SML or SM-Chol (molar %)	DSPE-PEG$_{2K}$ (molar %)	IRI DLC (%)	IRI DLE (%)	DLS Size by intensity (d.nm)	Zeta Potential (mV)	PDI
IRI/Lipo-PChcPC	0	95 (eq. 64.4% Chol)	5	5.71	55.23	107.2 ± 5.3	-9.5 ± 2.4	0.189 ± 0.052
IRI/Lipo-PChemsPC	0	95 (eq. 64.4% Chol)	5	6.31	64.68	109.4 ± 4.2	-10.2 ± 3.2	0.204 ± 0.036
IRI/Lipo-OChemsPC	0	95 (eq. 64.4% Chol)	5	6.43	67.58	105.7 ± 6.1	-5.2 ± 1.2	0.216 ± 0.017
IRI/Lipo-DChemsPC	0	95 (eq. 97.4% Chol)	5	5.68	57.36	104.9 ± 4.8	-6.3 ± 2.3	0.193 ± 0.023
IRI/Lipo-SML-SS	0	95 (eq. 64.4% Chol)	5	6.87	71.22	108.3 ± 5.2	-5.2 ± 2.4	0.205 ± 0.024
IRI/Lipo-SML-CSS	0	95 (eq. 64.4% Chol)	5	6.95	72.39	115.1 ± 4.5	-3.5 ± 2.1	0.186 ± 0.012
IRI/Lipo-SM-CSS-Chol	41.15	53.85 (eq. 35% Chol)	5	9.82	98.01	113.5 ± 6.0	-4.7 ± 1.8	0.172 ± 0.011

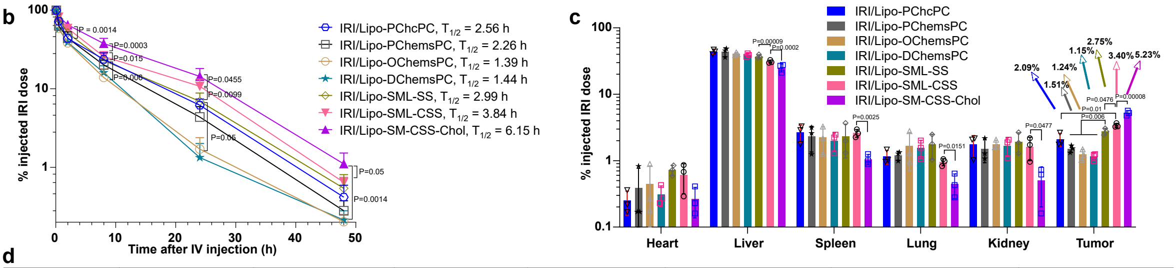

d

PK Parameter	IRI/Lipo-PChcPC	IRI/Lipo-PChemsPC	IRI/Lipo-OChemsPC	IRI/Lipo-DChemsPC	IRI/Lipo-SML-SS	IRI/Lipo-SML-CSS	IRI/Lipo-SM-CSS-Chol
$T_{1/2}$ (h)	2.56±0.85	2.26±0.99	1.39±0.17	1.44±0.05	2.99±0.17	3.84±0.69	6.15±1.05
V ((µg)/(µg/ml))	1.08±0.05	1.10±0.05	1.06±0.03	1.08±0.02	1.08±0.03	1.06±0.00	1.09±0.01
CL(µg)/(µg/ml)/h)	0.32±0.11	0.39±0.17	0.53±0.05	0.52±0.02	0.25±0.01	0.20±0.03	0.13±0.02
AUC_{0-t} (µg/ml*h)	2,699.29±802.22	2,339.17±928.07	1,508.59±141.72	1,539.77±72.97	3,214.25±182.26	4,169.16±764.84	6,477.01±1,015.14
AUC_{0-inf} (µg/ml*h)	2,699.33±802.25	2,339.21±928.13	1,508.59±141.72	1,539.77±72.97	3,214.30±182.29	4,170.58±766.75	6,513.92±1,046.73
AUMC (µg/ml*h^2)	10,618.95±5,707.13	8,499.81±6,324.90	3,057.54±674.43	3,194.13±269.53	13,914.01±1,534.97	23,633.52±8,695.09	58,804.73±18,963.57
MRT (h)	3.69±1.22	3.26±1.42	2.01±0.25	2.07±0.08	4.32±0.25	5.54±0.99	8.87±1.51
V_{ss} (µg/(µg/ml))	1.08±0.05	1.10±0.05	1.06±0.03	1.08±0.02	1.08±0.03	1.06±0.00	1.09±0.01

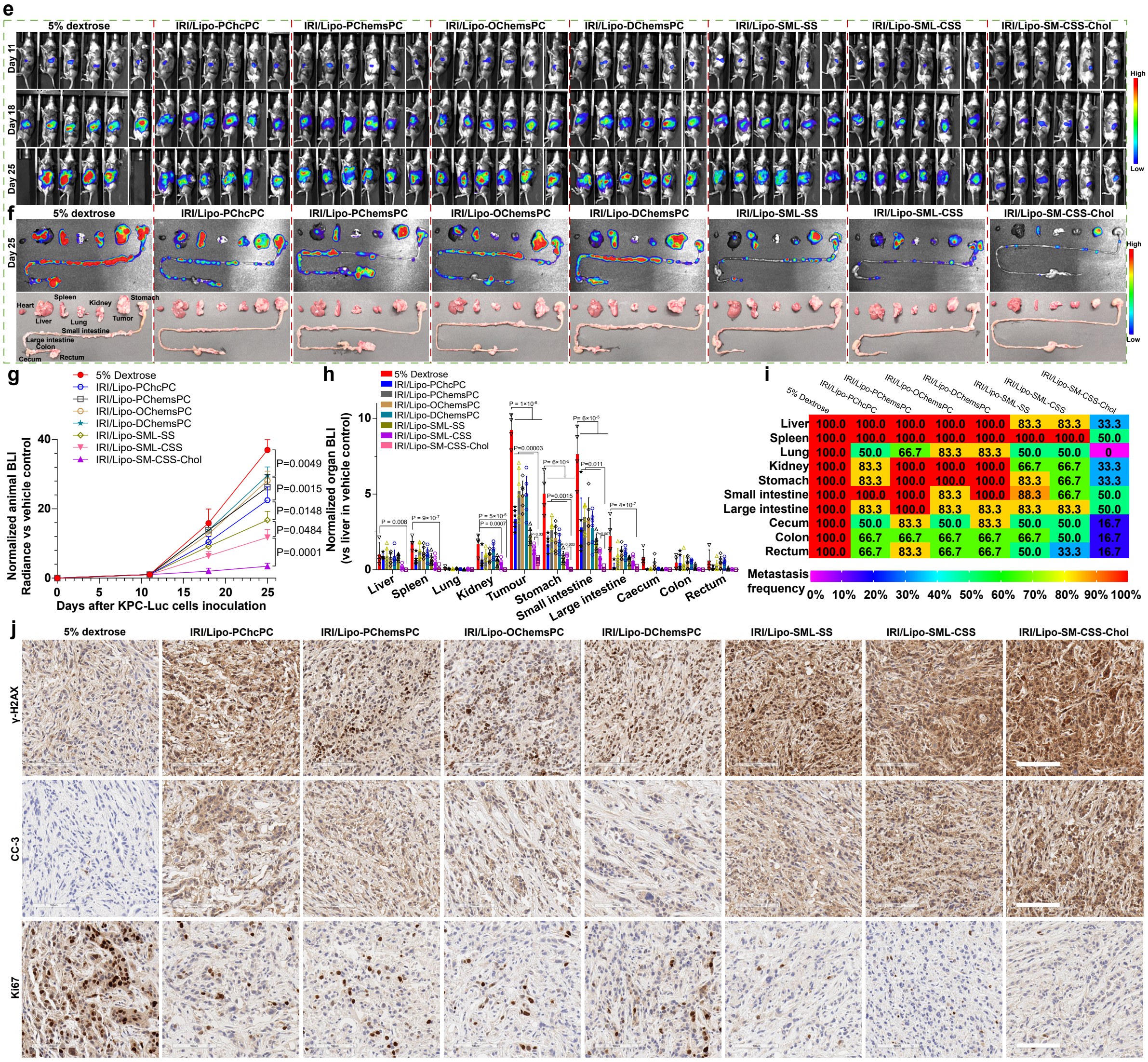

Supplementary Figure 42. IRI/Lipo-SM-CSS-Chol outperformed all IRI/Lipo-SMLs on therapeutic efficacy in late-stage metastatic orthotopic KPC-Luc PDAC mouse model. a, A table showing the physicochemical characterizations of various IRI/Lipo with 95 mol% SML and 5 mol% DSPE-PEG$_{2K}$ (eq. 64.4 mol% Chol

for IRI/Lipo-SML, except eq. 97.4 mol% Chol for IRI/Lipo-DChemsPC since two Chol molecules were consisted in DChemsPC) according to the previous report[4,7,11], to keep consistent, eq. 35 mol% Chol was used for IRI/Lipo-SM-CSS-Chol (n =3 independent samples). **b-d,** Blood kinetics (**b**), biodistribution (**c,** at 48 h post i.v. injection) and pharmacokinetic parameters (**d**) of IRI/Lipo in orthotopic KPC-Luc PDAC mouse model (n = 3 mice; tumour: ~400 mg) following a single i.v. administration at 40 mg IRI/kg. **e-i**, Therapeutic efficacy in metastatic orthotopic PDAC tumour mouse model. 2 × 10^6 cells were injected into the pancreas of B6129SF1/J mice (n = 6 mice). On day 11, the primary tumors reached ~400 mg with noticeable metastasis and mice were intravenously injected by various IRI/Lipo at 40 mg IRI/kg on day 11, 14, and 17. **e**, Mice Lago bioluminescence imaging (BLI) on day 11, 18 and 25. Two mice in group A died on day 24. **f**, Representative *ex vivo* BLI (upper panel) and photographs (lower panel) for various organs on day 25. **g**, Normalized BLI for whole mice tumour burden. Normalized BLI in various organs (**h**) and a heatmap summarizing tumour metastatic rate (**i**) on day 25. **j**, Representative IHC staining for γ-H2AX, CC-3, and Ki67 in orthotopic KPC-Luc tumors from **e**, scale bar = 100μm (n = 6 tumours, similar results were observed). Data in **a** (right portion), **b-d**,**g**,**h** are expressed as mean ± s.d. Statistical significance was determined by one-way ANOVA followed by Tukey's multiple comparisons test. Source data are provided as a Source Data file.

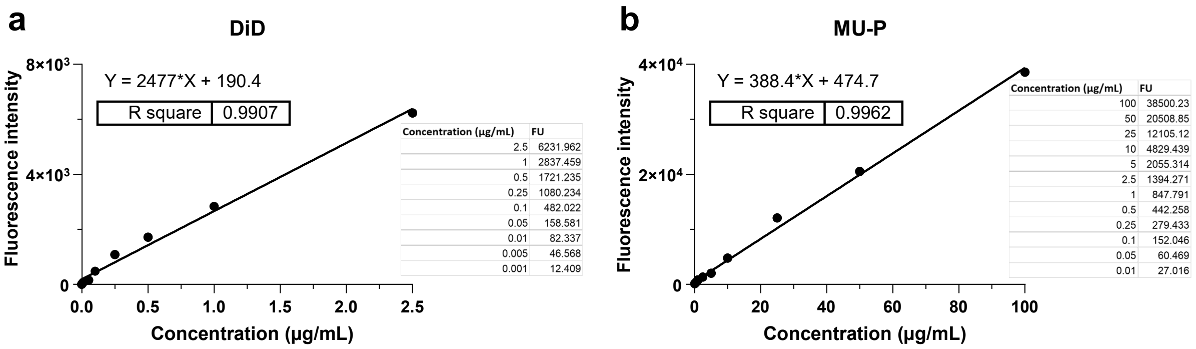

Supplementary Figure 43. Fluorescence intensity standard curves for DiD (**a**, excitation 644 nm, emission 664 nm) and MU-P (**b**, excitation 320 nm, emission 385 nm) concentration measurement in pharmacokinetics studies in **Supplementary Figure 37**. Source data are provided as a Source Data file.

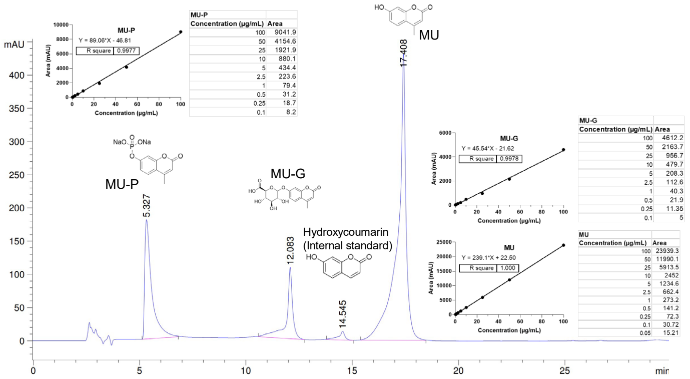

**Instrumentation**	
**HPLC Unit**	Agilent 1100 (USA)
**Detector**	Agilent G1315B Diode-Array Detector
**Integrator**	Agilent ChemStation Software
**Chromatographic conditions**	
**Column**	Waters Symmetry ODS (250mm X 4 mm X 5 μm)
**Mobile Phase**	ACN : NH_4Ac (15 mM) (70:30 v/v)
**Flow Rate**	1 mL/min
**Injection Volume**	50 μL
λ_{max}	320 nm

Supplementary Figure 44. The Analytic Reverse-phase High Performance Liquid Chromatography (HPLC) method development for MU-P, MU and MU-G concentration measurement in biodistribution studies. Representative HPLC chromatogram, standard curves, and HPLC instrumentation and chromatographic conditions for MU-P, MU and MU-G. Hydroxycoumarin was used as the internal standard[7]. Source data are provided as a Source Data file.

DOX-laden Lipo	DOX DLC (%)				DOX DLE (%)			
	#1	#2	#3	Mean ± SD	#1	#2	#3	Mean ± SD
DOX/Lipo-PChcPC	8.24	7.93	8.46	8.21 ± 0.27	73.46	72.56	78.47	74.83 ± 3.18
DOX/Lipo-SM/Chol	8.26	8.66	8.94	8.62 ± 0.34	82.69	86.66	89.49	86.28 ± 3.42
DOX/Lipo-SM-C-Ester-Chol	8.81	9.19	8.40	8.80 ± 0.40	89.69	92.01	82.34	88.01 ± 5.05
DOX/Lipo-SM-Ester-Chol	9.43	8.68	9.34	9.15 ± 0.41	94.32	86.91	93.39	91.54 ± 4.04
DOX/Lipo-SM-Glycine-Chol	6.89	7.40	7.46	7.25 ± 0.31	68.98	74.44	74.23	72.55 ± 3.09
DOX/Lipo-SM-CSS-Chol	8.98	9.19	8.86	9.01 ± 0.17	89.89	91.95	88.54	90.13 ± 1.72
DOX/Lipo-SM-SCS-Chol	9.26	8.46	8.50	8.74 ± 0.45	92.21	84.63	85.46	87.43 ± 4.16

Supplementary Figure 45. The DLC and DLE of various DOX/Lipo (n = 3 independent samples). Source data are provided as a Source Data file.

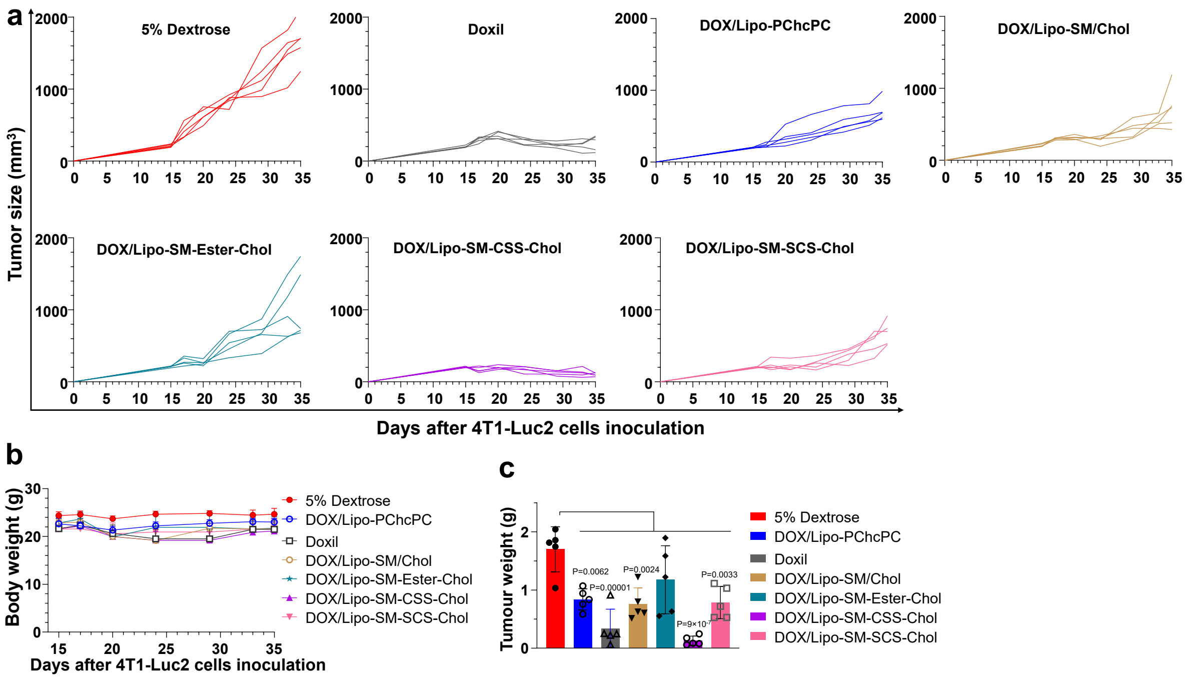

Supplementary Figure 46. Individual tumor growth curve (**a**), mice body weight (**b**) and tumour weight on day 35 (**c**) in therapeutic efficacy study presented in **Fig. 5**. Data in **b, c** are represented as mean ± s.d. (n = 5 mice). Statistical significance was determined by one-way ANOVA followed by Tukey's multiple comparisons test. Source data are provided as a Source Data file.

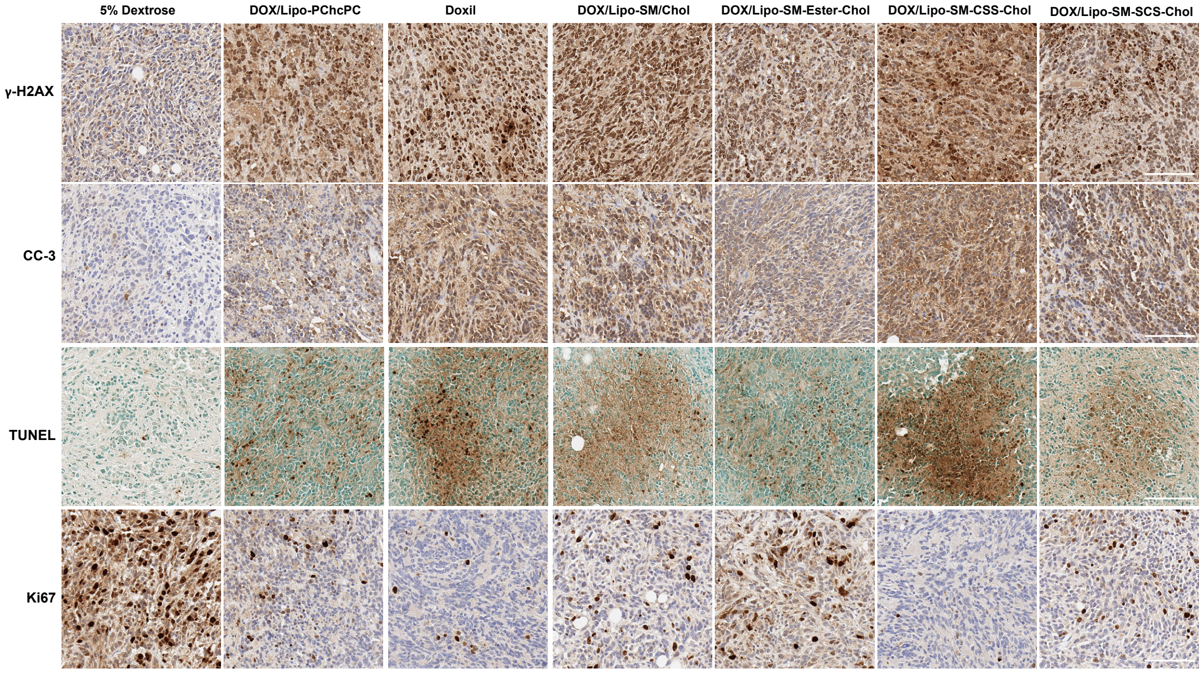

Supplementary Figure 47. Representative IHC staining for γ-H2AX, CC-3, TUNEL and Ki67 in orthotopic 4T1-Luc2 tumours from **Fig. 5c-f**. (n = 5 tumours, scale bar: 100 μm, similar results were observed).

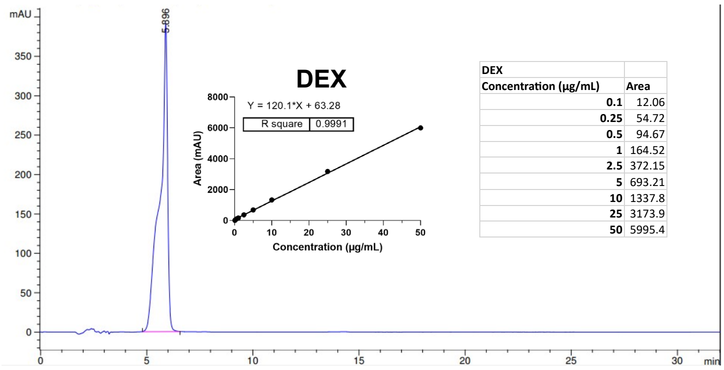

Instrumentation	
**HPLC Unit**	Agilent 1100 (USA)
**Detector**	Agilent G1315B Diode-Array Detector
**Integrator**	Agilent ChemStation Software
**Chromatographic conditions**	
**Column**	Waters Symmetry ODS (250mm X 4 mm X 5 µm)
**Mobile Phase**	ACN : NH_4Ac (15 mM) (60:40 v/v)
**Flow Rate**	1 mL/min
**Injection Volume**	50 µL
λ_{max}	240 nm

Supplementary Figure 48. The Analytic Reverse-phase High Performance Liquid Chromatography (HPLC) method development for DEX concentration measurement in drug loading studies. Representative HPLC chromatogram, standard curve, and HPLC instrumentation and chromatographic conditions for DEX. Source data are provided as a Source Data file.

a

DEX-laden Lipo	DEX DLC (%)				DEX DLE (%)			
	**#1**	**#2**	**#3**	**Mean ± SD**	**#1**	**#2**	**#3**	**Mean ± SD**
**DEX/Lipo-PChcPC**	0.066	0.12	0.081	0.09 ± 0.03	0.85	2.98	1.54	1.79 ± 1.09
**DEX/Lipo-SM/Chol**	0.18	0.05	0.07	0.10 ± 0.07	3.05	1.2	1.89	2.05 ± 0.93
**DEX/Lipo-SM-C-Ester-Chol**	0.26	0.14	0.17	0.19 ± 0.06	4.56	3.08	3.89	3.84 ± 0.74
**DEX/Lipo-SM-Ester-Chol**	0.46	0.86	0.21	0.51 ± 0.33	9.24	17.06	4.29	10.20 ± 6.44
**DEX/Lipo-SM-Glycine-Chol**	0.12	0.29	0.34	0.25 ± 0.12	2.48	5.86	6.87	5.07 ± 2.30
**DEX/Lipo-SM-CSS-Chol**	0.26	0.85	0.78	0.63 ± 0.32	5.28	17.04	15.68	12.67 ± 6.43
**DEX/Lipo-SM-SCS-Chol**	0.48	0.95	0.46	0.63 ± 0.28	9.61	19.03	9.2	12.61 ± 5.56

b **c**

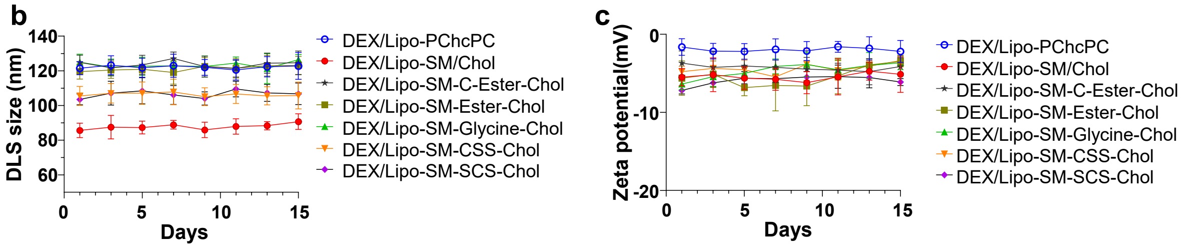

Supplementary Figure 49. (**a**) The DLC and DLE of various DEX/Lipo (n = 3 independent samples). DLS Size (**b**) and Zeta potential (**c**) monitoring for DEX/Lipo-PChcPC, DEX/Lipo-SM/Chol and various DEX/Lipo-SM-Chol over a 15-day period at 4 °C. Data are represented as mean ± s.d. (n = 3 independent experiments). Source data are provided as a Source Data file.

a

siRNA-laden LNP	DLin-MC3-DMA (molar %)	DSPC,SM (molar %)	Chol, SM-Chol or PChcPC (molar %)	PEG2000-C-DMG (molar %)	DLS size by intensity (d.nm)	Zeta potential (mV)	PDI
siRNA/LNP-DMA/DSPC/Chol (**A**)	49.3	10.2	39.0	1.5	183.80 ± 7.19	1.47 ± 0.68	0.20 ± 0.03
siRNA/LNP-DMA/PChcPC (**B**)	50.0	0	48.5 (eq. 39% Chol)	1.5	170.90 ± 6.25	3.80 ± 0.43	0.27 ± 0.05
siRNA/LNP-DMA/SM/Chol (**C**)	49.3	10.2	39.0	1.5	168.43 ± 6.04	1.52 ± 0.89	0.29 ± 0.04
siRNA/LNP-DMA/SM-C-Ester-Chol (**D**)	34.5	0	64.0 (eq. 39% Chol)	1.5	178.33 ±12.49	3.39 ± 0.19	0.29 ± 0.07
siRNA/LNP-DMA/SM-Ester-Chol (**E**)	34.5	0	64.0 (eq. 39% Chol)	1.5	173.53 ± 6.19	2.76 ± 1.05	0.18 ± 0.03
siRNA/LNP-DMA/SM-Glycine-Chol (**F**)	34.5	0	64.0 (eq. 39% Chol)	1.5	184.90 ± 6.24	1.18 ± 0.16	0.21 ± 0.04
siRNA/LNP-DMA/SM-CSS-Chol (**G**)	34.5	0	64.0 (eq. 39% Chol)	1.5	177.33 ± 4.99	1.39 ± 0.35	0.29 ± 0.03
siRNA/LNP-DMA/SM-SCS-Chol (**H**)	34.5	0	64.0 (eq. 39% Chol)	1.5	166.33 ± 3.41	4.25 ± 0.94	0.29 ± 0.04

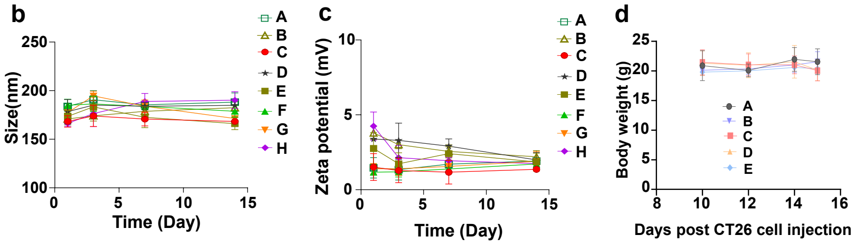

Supplementary Figure 50. Development, and physicochemical characterizations of siRNA-laden LNP. (**a**) A table showing the physicochemical characterizations of siRNA-laden LNP with regards to size, zeta potential and PDI. DLS size (**b**) and zeta potential (**c**) monitoring over a 15-day period at 4 °C. Mice body weight monitoring (**d**) in therapeutic efficacy study presented in **Fig. 6 g,h**. Data are represented as mean ± s.d. (n = 3 independent experiments for **a-c**; n = 5 mice for **d**). Source data are provided as a Source Data file.

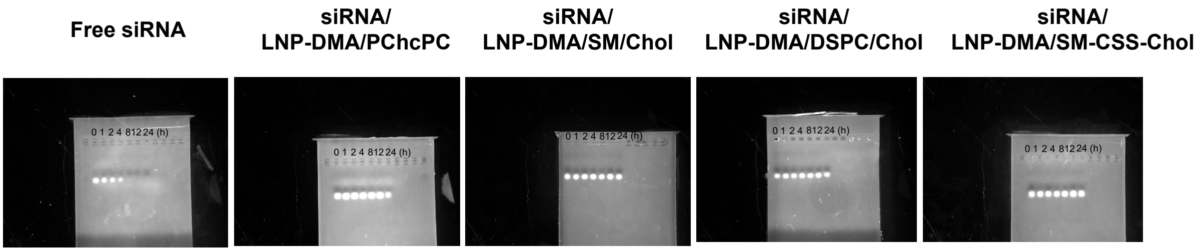

Supplementary Figure 51. The gel electrophoresis raw scans for **Fig. 6g**. Source data are provided as a Source Data file.

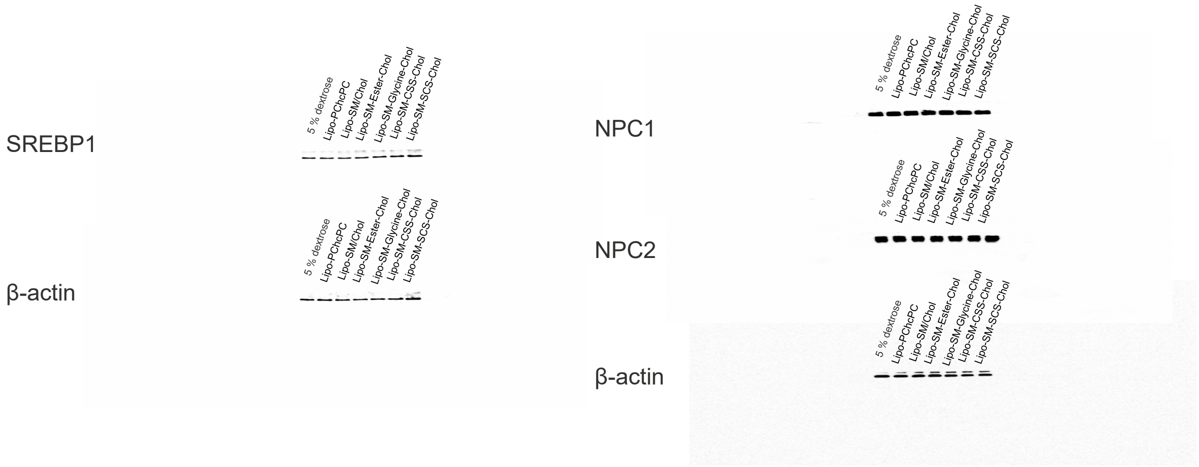

Supplementary Figure 52. The western blot raw scans for **Supplementary Figure 29.** Source data are provided as a Source Data file.

Supplementary References

1. Wang Z*, et al.* Immunogenic camptothesome nanovesicles comprising sphingomyelin-derived camptothecin bilayers for safe and synergistic cancer immunochemotherapy. *Nat Nanotechnol*, **16,** 1130-1140 (2021).
2. Yin W*, et al.* Integrated block copolymer prodrug nanoparticles for combination of tumor oxidative stress amplification and ROS-responsive drug release. *Biomaterials*, **195,** 63-74 (2019).
3. Matyash V, Liebisch G, Kurzchalia TV, Shevchenko A, Schwudke D. Lipid extraction by methyl-tert-butyl ether for high-throughput lipidomics. *J Lipid Res*, **49,** 1137-1146 (2008).
4. Huang Z, Szoka FC, Jr. Sterol-modified phospholipids: cholesterol and phospholipid chimeras with improved biomembrane properties. *J Am Chem Soc*, **130,** 15702-15712 (2008).
5. Wybenga DR, Pileggi VJ, Dirstine PH, Giorgio JD. Direct Manual Determination of Serum Total Cholesterol with a Single Stable Reagent. *Clinical Chemistry*, **16,** 980-984 (1970).
6. Zhang Y, Huo M, Zhou J, Xie S. PKSolver: An add-in program for pharmacokinetic and pharmacodynamic data analysis in Microsoft Excel. *Comput Methods Programs Biomed*, **99,** 306-314 (2010).
7. Kohli AG, Kieler-Ferguson HM, Chan D, Szoka FC. A robust and quantitative method for tracking liposome contents after intravenous administration. *J Control Release*, **176,** 86-93 (2014).
8. Lu J*, et al.* Nano-enabled pancreas cancer immunotherapy using immunogenic cell death and reversing immunosuppression. *Nat Commun*, **8,** 1811 (2017).
9. Thomson NH*, et al.* Atomic force microscopy of cationic liposomes. *Langmuir*, **16,** 4813-4818 (2000).
10. Nakano K, Tozuka Y, Yamamoto H, Kawashima Y, Takeuchi H. A novel method for measuring rigidity of submicron-size liposomes with atomic force microscopy. *International Journal of Pharmaceutics*, **355,** 203-209 (2008).
11. Huang Z, Jaafari MR, Szoka FC, Jr. Disterolphospholipids: nonexchangeable lipids and their application to liposomal drug delivery. *Angew Chem Int Ed Engl*, **48,** 4146-4149 (2009).
