# Supplementary material for: Cholesterol-modified sphingomyelin chimeric lipid bilayer for improved therapeutic delivery
Source: Nat Commun. 2024 Mar 7;15:2073. doi: 10.1038/s41467-024-46331-7 (PMC10920917; doi:10.1038/s41467-024-46331-7)
Supplement: Supplementary file 2 — Reporting Summary [file 41467_2024_46331_MOESM2_ESM.pdf]

nature portfolio

Corresponding author(s): Jianqin Lu

Last updated by author(s): Feb 20, 2024

Reporting Summary

Nature Portfolio wishes to improve the reproducibility of the work that we publish. This form provides structure for consistency and transparency in reporting. For further information on Nature Portfolio policies, see our Editorial Policies and the Editorial Policy Checklist.

Statistics

For all statistical analyses, confirm that the following items are present in the figure legend, table legend, main text, or Methods section.

n/a	Confirmed	
☐	☒	The exact sample size (*n*) for each experimental group/condition, given as a discrete number and unit of measurement
☐	☒	A statement on whether measurements were taken from distinct samples or whether the same sample was measured repeatedly
☐	☒	The statistical test(s) used AND whether they are one- or two-sided<br>*Only common tests should be described solely by name; describe more complex techniques in the Methods section.*
☐	☒	A description of all covariates tested
☐	☒	A description of any assumptions or corrections, such as tests of normality and adjustment for multiple comparisons
☐	☒	A full description of the statistical parameters including central tendency (e.g. means) or other basic estimates (e.g. regression coefficient) AND variation (e.g. standard deviation) or associated estimates of uncertainty (e.g. confidence intervals)
☐	☒	For null hypothesis testing, the test statistic (e.g. *F*, *t*, *r*) with confidence intervals, effect sizes, degrees of freedom and *P* value noted<br>*Give P values as exact values whenever suitable.*
☒	☐	For Bayesian analysis, information on the choice of priors and Markov chain Monte Carlo settings
☒	☐	For hierarchical and complex designs, identification of the appropriate level for tests and full reporting of outcomes
☒	☐	Estimates of effect sizes (e.g. Cohen's *d*, Pearson's *r*), indicating how they were calculated

Our web collection on statistics for biologists contains articles on many of the points above.

Software and code

Policy information about availability of computer code

Data collection	NMR spectra were acquired by Bruker topspin software (v. 2.1). HPLC spectra were acquired by ChemStation Rev.A. software (v. 10.01, Agilent Technology). DLS size and zeta potential data were acquired by Zetasizer software (v. 7.13). Cryo-EM image were acquired by Tecnai User Interface software (v. 3.1.5) and EMMenu. Fluorescence were acquired by SoftMax® Pro (v. 7.1.0). DSC data were acquired by VPViewer 2000 (v. 2.65). Histology images were acquired Leica DMI6000 microscope. Serum chemistry and hematological counts were acquired by Liasys 330 and Hemavet 950FS, respectively. Immunofluorescence images were acquired by Zeiss LSM880 inverted confocal microscope (Zen Black software (v. 14.022.021)). PCR data were acquired by Design & Analysis Software (v. 1.5.1, QuantStudio™). Pharmacokinetic parameters were acquired by PKSolver software (version 2.0). In vivo bioluminescence and fluorescence images were acquired by Aura 64 Bit Analysis software (v. 3.2.0). Histology images were acquired by Olympus VS200 slide scanner (version OlyVIA V4.1). H&E imaging were acquired by Leica DMI6000B microscope with a Leica DFC450 color camera and the Leica LAS X 3.7 software. Western blot images were taken by azure biosystem 600 (v.1.9.0.0406).
Data analysis	NMR data spectra were analyzed by MestReNova (v. 6.0.2). HPLC data and spectra were analyzed by ChemStation Rev.A. software (v. 10.01, Agilent Technology). In vivo bioluminescence and fluorescence images were analysed by Aura 64 Bit Analysis software (v. 3.2.0). All statistical analyses were performed with Graphpad Prism 8. Histology images were analyzed by the Leica microsystems, LAS X 3.x software (v. 3.7.3.23245). Fluorescence intensity was quantified by ImageJ software (Version.1.53q)

For manuscripts utilizing custom algorithms or software that are central to the research but not yet described in published literature, software must be made available to editors and reviewers. We strongly encourage code deposition in a community repository (e.g. GitHub). See the Nature Portfolio guidelines for submitting code & software for further information.

Data

Policy information about availability of data

All manuscripts must include a data availability statement. This statement should provide the following information, where applicable:

- Accession codes, unique identifiers, or web links for publicly available datasets
- A description of any restrictions on data availability
- For clinical datasets or third party data, please ensure that the statement adheres to our policy

All the data supporting the findings of this study are available within the article and its Supplementary Information. The full image dataset is available from the corresponding author upon request. Source data are provided with this paper.

Research involving human participants, their data, or biological material

Policy information about studies with human participants or human data. See also policy information about sex, gender (identity/presentation), and sexual orientation and race, ethnicity and racism.

Reporting on sex and gender	N/A
Reporting on race, ethnicity, or other socially relevant groupings	N/A
Population characteristics	N/A
Recruitment	N/A
Ethics oversight	N/A

Note that full information on the approval of the study protocol must also be provided in the manuscript.

Field-specific reporting

Please select the one below that is the best fit for your research. If you are not sure, read the appropriate sections before making your selection.

☒ Life sciences ☐ Behavioural & social sciences ☐ Ecological, evolutionary & environmental sciences

For a reference copy of the document with all sections, see nature.com/documents/nr-reporting-summary-flat.pdf

Life sciences study design

All studies must disclose on these points even when the disclosure is negative.

Sample size	The sample sizes were determined by the published literature (Nat. Nanotechnol. 2021,16(10):1130-1140)
Data exclusions	No data were excluded.
Replication	Most in vitro experiments were have 3 biologically independent samples or repeated independently for at least 3 times. All in vivo studies were repeated at least 5-6 mice per group. The detailed information is also described in the figure legends and methods section.
Randomization	The experimental groups were allocated randomly.
Blinding	No formal blinding was used. The serum chemistry, hematological counts, Cryo-EM was conducted by independent scientists, who were unaware of the treatment conditions, in respective core facilities. For other assays, the investigators were not blinded to the group allocation because the data analyses were based on objectively measurable data.

Reporting for specific materials, systems and methods

We require information from authors about some types of materials, experimental systems and methods used in many studies. Here, indicate whether each material, system or method listed is relevant to your study. If you are not sure if a list item applies to your research, read the appropriate section before selecting a response.

Materials & experimental systems

n/a	Involved in the study
	x Antibodies
	x Eukaryotic cell lines
x	Palaeontology and archaeology
	x Animals and other organisms
x	Clinical data
x	Dual use research of concern
x	Plants

Methods

n/a	Involved in the study
x	ChIP-seq
x	Flow cytometry
x	MRI-based neuroimaging

Antibodies

Antibodies used

Immunofluorescence Experiments:
β-Tubulin Rabbit mAb (Cell Signaling, Cat#: 3623S, 9F3, Alexa Fluor® 488 Conjugate, 1/100)
Cholera Toxin Subunit B (Thermo Fisher Scientific, Cat#: C22841, 9F3, Alexa Fluor® 488 Conjugate, 1/1000)
rabbit anti-lysenin antibody (MyBioSourc, Cat#: MBS406486, 1/1000) and Alexa Fluor® 488-anti-rabbit antibody (Abcam, Cat#: ab150077, 1/1000)

Immunohistochemistry Experiments:
anti cleaved caspase-3 (Cell Signaling, 5A1E, Cat#: 9664S, dilution: 1/300)
anti-gamma H2A.X (Abcam, 3F2, Cat#: ab22551, dilution: 1/400)
anti-Ki67 antibody (Abcam, Rabbit polyclonal, Cat#: ab15580, dilution: 1/400)
TUNEL assay kit-HRP-DAB (Abcam, Cat#: ab206386)

Western Blot Experiments:
anti-SREBP-1 (Abcam, Rabbit polyclonal, Cat#: ab28481, dilution: 1/1500)
anti-NPC1 (Abcam, EPR5209, Cat#: ab134113, dilution: 1/2000)
anti-NPC2 (Abcam, EPR19993-145-1, Cat#: ab218192, dilution: 1/2000)
β-actin (Abcam, EPR21241, Cat#: ab213262, dilution: 1/2000)

Validation

All antibodies were verified by the supplier's websites and/or results presented in the manuscript.
The detailed information are listed as below:
Immunofluorescence Experiments:
1) β-Tubulin Rabbit mAb; Rabbit; suitable for IF; reacts with Human, Mouse, Rat, Monkey, Zebrafish, Bovine.
2) Cholera Toxin Subunit B for studying Lipid Rafts; suitable for IF.
3) rabbit anti-lysenin antibody, rabbit, suitable for IF; reacts with Human, Mouse.

Immunohistochemistry Experiments:
1) anti cleaved caspase-3, rabbit; suitable for IHC-P; reacts with mouse, rat, monkey.
2) anti-gamma H2A.X, mouse; suitable for IHC-P; reacts with mouse, human.
3) anti-Ki67 antibody, rabbit; suitable for: IHC-P, ICC/IF; reacts with: mouse, human.
4) TUNEL assay kit-HRP-DAB, suitable for: IHC; reacts with: mouse, human.

Western Blot Experiments:
1) anti-SREBP-1, Rabbit, suitable for: ICC/IF, WB, reacts with: mouse, rat, human.
2) anti-NPC1, Rabbit, suitable for: WB, IHC-P, ICC/IF, reacts with: mouse, rat, human.
3) anti-NPC2, Rabbit, suitable for: WB, IHC-P, ICC/IF, reacts with: mouse, rat, human.
4) β-actin, Rabbit, suitable for: WB, IHC-P, ICC/IF, reacts with: mouse, rat, human.

Eukaryotic cell lines

Policy information about cell lines and Sex and Gender in Research

Cell line source(s)

Mouse colon carcinoma cell line CT26 (Cat. ATCC CRL-2638) and mouse breast cancer cell line 4T1 (Cat. CRL-2539, female) were obtained from UArizona Cancer Center. Mouse breast cancer cells 4T1-Luc2 (Cat. CRL-2539-LUC2, female) was obtained from American Type Culture Collection (ATCC). MC38 (Cat. ENH204-FP) was purchased from Kerafast. Mouse pancreatic cancer cells KPC-Luc (Cat. 153474) was provided by Professor Gregory Beatty at University of Pennsylvania. Human diffuse

	large B-cell lymphoma cell line SU-DHL-4 (Cat. CRL-2957) was provided by Professor Catharine Smith at The University of Arizona
Authentication	Cell lines were used without any modification once received from respective suppliers and therefore were not authenticated
Mycoplasma contamination	All cell lines were regularly tested for mycoplasma contamination and no mycoplasma contamination was observed.
Commonly misidentified lines (See ICLAC register)	None of the cell lines used are listed in the ICLAC list

Animals and other research organisms

Policy information about studies involving animals; ARRIVE guidelines recommended for reporting animal research, and Sex and Gender in Research

Laboratory animals	CB17/Icr-Prkdcscid/IcrIcoCrl (Charles Rivers, 6 weeks old, female), and BALB/c, C57BL/6 and B6129SF1/J mice (Jackson laboratory, ~6 weeks old, female) were used. Mice were housed in Standard Individually Ventilated Caging (IVC). The Light cycle is 12/12 – 12 hours light/12 hours dark with 7am on-7pm off. The Temperature is maintained between 68°-72°F and the humidity is between 30-70% per the NIH Guide.
Wild animals	The study did not involve wild animals.
Reporting on sex	To ensure gender uniformity, the mice used in this study were female due to risk of more fighting in male colonies. Still, here analysis is compared with or without treatment, so the sex of the host is considered less important.
Field-collected samples	No Field-collected sample were used in this study
Ethics oversight	All animal experiments were approved by The University of Arizona Institutional Animal Care and Use Committee (IACUC)

Note that full information on the approval of the study protocol must also be provided in the manuscript.

Plants

Seed stocks	N/A
Novel plant genotypes	N/A
Authentication	N/A
